# Photodegradation Driven by Visible Light Exceeds Biodegradation across a Range of Environmental Conditions during the Initial Hours after an Oil Spill

**DOI:** 10.1021/acs.est.5c06941

**Published:** 2025-09-26

**Authors:** Alice C. Ortmann, Brian Robinson, Ho-Yin Poon, Thomas L. King

**Affiliations:** Bedford Institute of Oceanography, Fisheries and Oceans Canada, 1 Challenger Drive, Dartmouth, NS, Canada, B2Y 4A2

**Keywords:** oil spill, photodegradation, biodegradation, natural attenuation, photooxidation, organic matter

## Abstract

Degradation of oil compounds, mediated by microbes or abiotic factors, after a spill into aquatic environments is important for ecosystem recovery; however, factors controlling these processes in natural waters remain poorly characterized. Six experiments representing a range of environmental conditions measured the loss of *n*-alkanes and polycyclic aromatic compounds (PACs) from a crude oil over 72 h due to biodegradation and abiotic processes when exposed to visible light. On average, 22.8% (±12.6) of *n*-alkanes and 32.7% (±14.0) of PACs were removed in the live incubations compared to 19.1% (±19.3) of *n*-alkanes and 31.7% (±12.3) of PACs when microbial activity was inhibited by the addition of mercuric chloride. Substantial biodegradation was only observed in the warm, freshwater incubation. Additional experiments determined that most of the abiotic degradation could be attributed to photodegradation mediated by visible light. No clear link was observed between the environmental conditions and abiotic losses of oil compounds, indicating complex interactions between light, photosensitizers, and organisms. These experiments demonstrate that a significant proportion of the *n*-alkanes and PACs may be removed from a surface oil spill through photodegradation mediated by both visible and UV light, along with biodegradation, within a few days.

## Graphical abstract

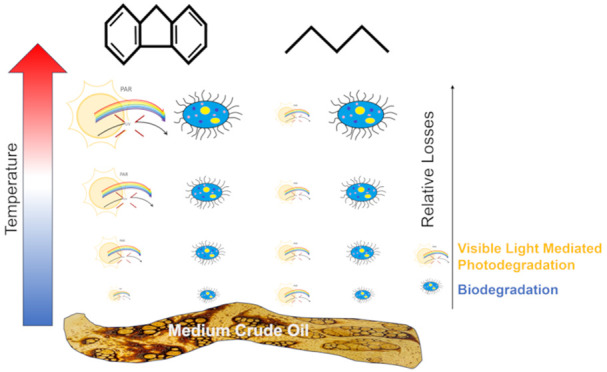

## Introduction

Immediately following a spill into seawater, oil begins to undergo multiple weathering processes.^1,2^ Many of these processes result in the redistribution of all or part of the oil throughout the environment, affecting concentrations and location of the oil. Biodegradation and photodegradation alter the chemical composition of the oil,^3–6^ which changes its physical and chemical properties, including its potential toxicity.^4,7–9^ These changes also affect the ability of responders to monitor and track the oil.

While oil is a complex mixture of potentially thousands of compounds, most monitoring of oil focuses on a small number of compounds. This is partly a logistical issue; monitoring the multiple types of compounds in oil requires multiple analytical approaches, few of which have been translated into standard protocols that can be conducted by certified laboratories. It is also an issue of focusing on those compounds thought to represent the largest risks to human and environmental health. The 16 priority polycyclic aromatic hydrocarbons (PAHs) identified by the Environmental Protection Agency of the United States represent compounds with high potential to cause acute toxicity that are also readily quantified by standard analyses.^10^ No single approach is mandated to monitor oil during a spill; however, gas chromatography coupled to mass spectrometry (GC-MS) is commonly used.^11^ GC-MS analyses are capable of providing estimates for specific compounds, including short *n*-alkanes, the 16 priority PAHs, and many of their alkylated homologues. The compounds identified through these techniques are also likely the most bioavailable to the oil degrading microbial community.^12,13^

Biodegradation of oil compounds is carried out by consortia of microbes, mainly bacteria with some fungi and algae that differ based on location.^14,15^ Following an oil spill, a lag between the spill and observable biodegradation occurs as the oil degraders increase in number. The lag time depends on the microbial community structure, previous exposures to oil, as well as the temperature and nutrient availability.^16^ As biodegradation also requires access to oil compounds, the lag time also reflects the time needed for oil compounds and microbes to connect at oil–water interfaces^2^ through dissolution or dispersion of the oil, colonization of the interface at the surface, or through increased solubility through photodegradation. Photodegradation can support increased microbial activity^17^ and may contribute to shifts in the microbial community composition through increased bioavailability of many of the photoproducts.^7,16,18,19^

Photodegradation of oil compounds is driven mainly by UV radiation, with direct degradation acting on the aromatic rings of the polycyclic aromatic compounds (PACs = PAHs + N and S containing aromatics) and indirect degradation of other oil compounds, including *n*-alkanes, branched alkanes, and cycloalkanes, mediated through the production of reactive oxygen species.^20^ While larger PACs can absorb light in the visible range, photodegradation with visible light often depends on photocatalysts.^20^ In natural waters, photocatalysts may include organic matter, nitrite, nitrate, trace nutrients, and inorganic particles or living organisms.^21–23^ The contribution of photodegradation due to visible light has not been directly investigated, with previous studies suggesting that it is a minor contributor relative to the degradation mediated through UV light.^20^ Some recent studies have suggested changes due to visible light may be as large as those driven by full sunlight or UV.^24,25^

Controlled studies can provide insights into the mechanisms underlying both the biodegradation and photodegradation of oil; however, the interaction among different factors under real-world conditions makes it challenging to predict specific rates. Increasing temperature has been shown to increase photodegradation rates for PACs^26–28^ but also increases biodegradation with higher microbial growth rates and enzyme activity.^29^ Higher temperatures also increase dissolution into the water column and losses through volatilization. Photodegradation increases with increasing light intensity and exposure time,^27^ which may also increase phototrophic microbial activity. Salinity, important in structuring the microbial community,^30,31^ which can affect biodegradation,^32,33^ has not been identified as a factor affecting the photodegradation of oil.^27,34^ Understanding how these factors interact to affect photodegradation and biodegradation can contribute to improving models for oil spill fate and transport during spill response.^16,35^

An initial series of experiments were conducted to estimate the potential for biodegradation to transform *n*-alkanes and 2–5-ring PACs using natural surface water collected from six different sites in eastern Canada. These experiments were designed to support phototrophic activity by utilizing lights that provided photosynthetically active radiation (PAR, 400–700 nm) to determine how biodegradation varied under different environmental conditions. The focus was on the responses in the first 72 h post oil exposure, when oil would have the highest concentration and microbial responses would be initiated. A series of additional experiments were conducted to quantify which processes could be responsible for the abiotic losses of *n*-alkanes and PACs observed in the absence of biodegradation.

## Methods

### Chemicals

Heidrun medium crude oil was decanted from a 55 gallon drum stored at the Bedford Institute of Oceanography (BIO) in August 2020. The barrel was obtained from the Canadian Coast Guard in Matane, QC, Canada, in March 2008. Dichloromethane (DCM) distilled in glass grade was purchased from Caledon Laboratories (Georgetown, ON, Canada). Saturated mercuric chloride (HgCl_2_) was obtained from Ricca Chemical Company (Arlington, TX, USA). Calibration and surrogate standards for GC-MS were prepared using certified reference materials (Absolute Standards, Hamden, CT, USA) and neat standards (Chiron, Trondheim, Norway; Sigma-Aldrich, Oakville, ON, Canada).

### Field Experiments

Surface water (2 m) was collected with a SBE911 conductivity, temperature, and depth (CTD)/rosette system (Sea-Bird Scientific, Bellevue, WA, USA) during two different missions in August 2020 on the R/V *Coriolis II* and in August 2021 on the CCGS *Hudson*. In 2020, water was collected from three stations within the St. Lawrence River estuary, while the 2021 samples were collected from Placentia Bay, NL, Canada, the Cabot Strait, and Chedabucto Bay, NS, Canada (Figure 1). The CTD was equipped with an SBE43 O_2_ sensor (Sea-Bird Scientific). In 2019, turbidity was measured with a Sea-Bird Scientific ECO-NTU turbidity meter, whereas in 2020 a Sea-Bird Scientific ECO-BB meter was used. The ECO-BB measures the beam attenuation at 650 nm with a resolution of 3.62 × 10^–6^ m^–1^ sr^–1^, which was used to calculate the backscatter coefficient based on the configuration of the instrument.

**Figure 1:**
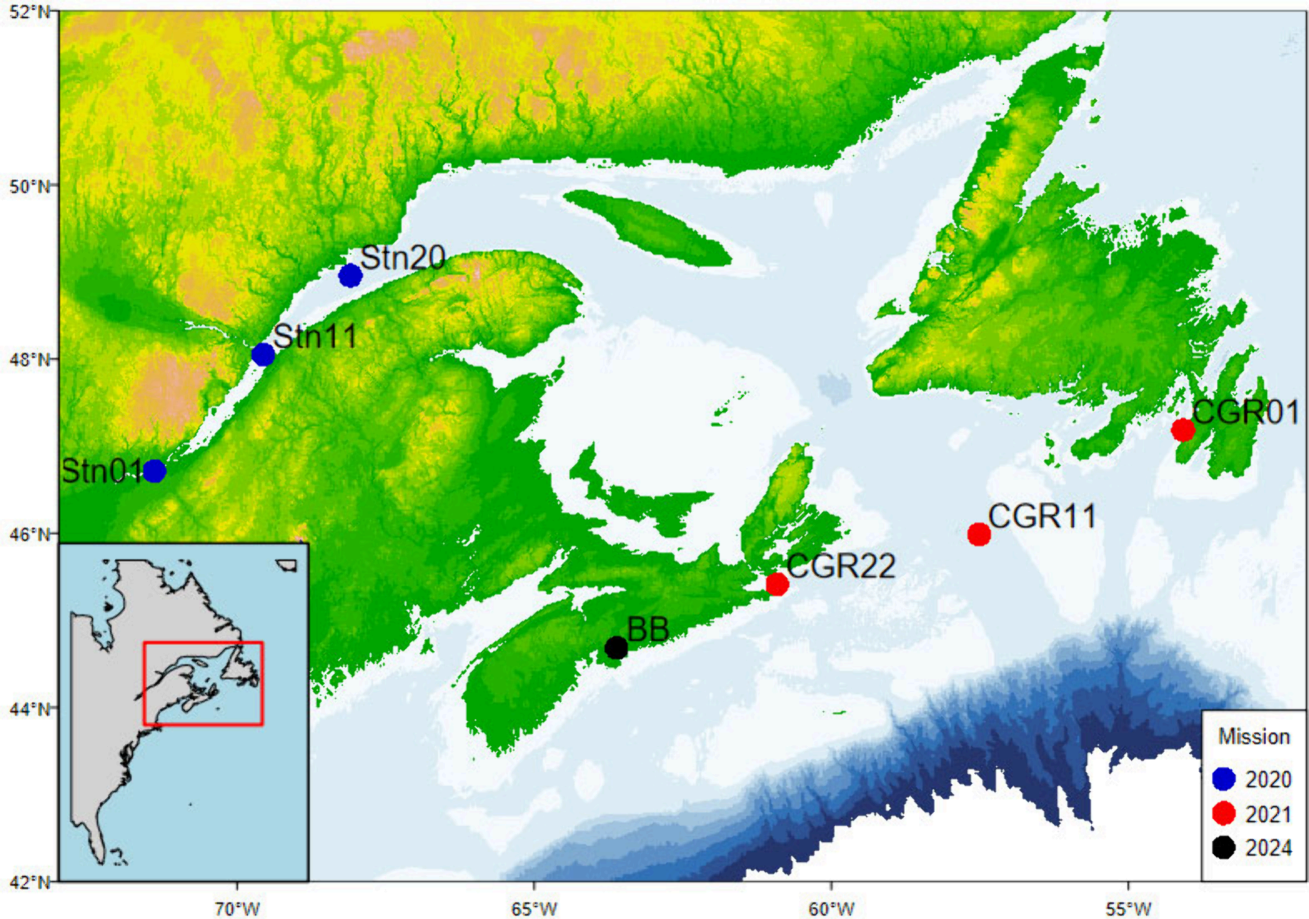
Map of eastern Canada showing six locations colored by year where experiments were conducted for biodegradation and photodegradation estimates and the Bedford Basin where water for the additional experiment was collected. Inset shows the east coast of North America with the box indicating the sample area.

Immediately following collection, water (120 mL) was distributed into 250 mL Type II soda lime glass jars with Teflon-lined plastic lids, leaving >1/2 of the volume as air. Jars were assigned to one of two treatments: live incubations, which received ∼20 μL of Heidrun, a medium crude oil, via a positive displacement pipet, or killed controls, which received the oil as well as 100 μL of a HgCl_2_ solution (180 μM final concentration). Triplicate jars from each treatment immediately received 15 mL of DCM followed by shaking and venting to partially extract the organic material. Jars were then stored at 4 °C until they were analyzed at BIO.

The remaining jars were placed on an orbital shaker set at 150 rpm in an incubator set at the temperature of the water, as measured by the CTD. Above the jars was a broad-spectrum LED light (Shenzhen Phlizon Science and Technology Co., Ltd., China) on a timer. Lights were set to turn on and off to provide 15 h of light, which was the average day length where and when the experiments were being conducted. A Spherical Quantum Sensor (LI-COR, Lincoln, NE, USA) measured an average of 400 μmol s^–1^ m^–2^ PAR near the surface of the shaker. Output of the LED lights was measured using an SRI-2000UV Spectrophotometer (Optimum Optoelectronics Corp., Chubei, Tiawan). Light ranged from 411 to 761 nm, with peaks at 448, 601, and 658 nm, which is within the visible range (380–780 nm). A PAR fixture was selected to support phototrophic activity (photosynthesis and photoheterotrophy) in the live incubations while limiting the photodegradation of oil compounds due to UV irradiation. The glass reduced the intensity of the light by approximately 20% throughout most of the spectra, with greater attenuation at the extremes. When the lights were on, the temperature in the incubations increased by ∼3 °C. After 36 and 72 h, 15 mL of DCM was added to 3 jars from each treatment to stop degradation as described above.

Additional water from each station was analyzed to determine the concentration of inorganic nutrients, total organic carbon (TOC), chlorophyll *a* (chl *a*), and the abundance of prokaryote cells. Briefly, nitrate, ammonium, phosphate, and silicate were measured on a SEAL Analytical AS3 continuous segmented flow autoanalyzer (SEAL Analytical Inc., Mequon, WI, USA),^36^ while TOC was measured as the difference between total carbon and total inorganic carbon on a VARIO Select TOC analyzer (Elementar Americas Inc., Ronkonkoma, NY, USA).^37^ Chl *a* was measured using the snap-in chl *a* acidification module on a Turner Trilogy fluorometer (Turner Designs, San Jose, CA, USA) after extraction from 25 mm GF/F filters, which had collected phytoplankton from 100 mL of water.^38^ Prokaryote cells were quantified using a BD FACSLyric flow cytometer (BD Biosciences, Franklin Lakes, NJ, USA) after staining with SYBR Green.^37^

### Laboratory Experiments

Following the field experiments, subsequent experiments were conducted in 2024 to quantify the degradation in killed samples under light and dark conditions, determine if HgCl_2_ impacted the detection and degradation of oil compounds, and test the effectiveness of HgCl_2_ in inhibiting microbial growth. Experiments used either surface water collected from Bedford Basin, Halifax, NS, Canada (BB) (Figure 1) or 18.2 MΩ·cm water (Milli-Q) and were conducted at BIO. Surface water experiments were conducted immediately after water collection. All experiments used the same oil as before, and jars were incubated under either light or dark conditions, with or without HgCl_2_ (Table 1). Incubation temperatures were 14 °C with a day length of 13 h in April 2024. Milli-Q experiments were conducted at 15 °C with a day length of 15 h. Jars for dark incubations were wrapped in foil. At 0 and 72 h, 15 mL of DCM was added to three jars per treatment, which were stored at 4 °C until processing.

**Table 1:** Summary of the Experiments Conducted to Test the Effects of Biodegradation and Abiotic Degradation, Including Photodegradation and HgCl_2_

Experiment	Water Source	HgCl_2_ Added^a^	Light or Dark^a^	Times Sampled	Analysis	Purpose
Missions						
2020	2 m surface water	9, yes	18, light	0, 36, 72 h	GC-MS	Abiotic degradation
2021		9, no				Biodegradation
2024	BB	9, yes	3, light	0, 72 h	GC-MS	Abiotic degradation
			3, dark		Absorbance	Photodegradation
	Milli-Q water	9, yes	6, light	0, 72 h	GC-MS	Abiotic degradation
		9, no	6, dark		Absorbance	Photodegradation
						HgCl_2_ effects

a Values indicate the number of jars assigned to each treatment per experiment.

To determine if the jars were likely to have become anoxic over the 72 h, an additional set of jars with Bedford Basin water were incubated at 22 °C under continuous light with oil added. These conditions should maximize growth and O_2_ consumption. A hand-held oxygen probe (Professional Plus 2030, YSI Inc., Yellowsprings, OH, USA) was used to measure the O_2_ in the seawater at 0 and 72 h. The effectiveness of 180 μM HgCl_2_ to inhibit microbial growth was confirmed by spreading 50 μL of seawater and oil, with and without HgCl_2_, on Marine Broth 2216 agar plates (Difco, BD, Sparks, MD, USA) and incubating them at 22 °C for 72 h. Colonies were counted to determine the number of culturable cells (colony forming units = CFU mL^–1^).

### Chemical Analysis

Jars from both the field and lab experiments to which DCM had been added were extracted using a liquid–liquid extraction method, purified using a solid-phase extraction technique, and analyzed by GC-MS.^39^ Deuterated surrogates were added prior to extraction to calculate the recovery efficiencies. Quantified analytes included C_10_–C_35_ *n*-alkanes, PACs from 2 to 5 rings, including parent and alkylated compounds, as well as 17α­(H),21β­(H)-hopane. Due to low recovery of the deuterated C_12_ surrogate, only *n*-alkanes >C_13_ were included in the analysis. After analysis of the Bedford Basin and Milli-Q samples by GC-MS, 0.5 mL of each extract was diluted to 3.0 mL, and the absorbance of the extracts was measured from 240 to 500 nm on an Aqualog spectrophotofluorometer (HORIBA Ltd., Kyoto, Japan).

### Initial Degradation Rate Constants

For each sample, *n*-alkanes and PACs were normalized using 17α­(H),21β­(H)-hopane (hopane normalized).^40^ One sample with HgCl_2_ from 36 h from CGR11 was excluded from further analyses, as it was missing data for several analytes present in all other samples. Additionally, one 0 h sample was excluded from the calculations of degradation rate constants for the BB experiment, as the hopane normalized ratios differed greatly from those of the other two samples and an extract of the pure oil. Initial degradation rate constants were calculated as the slope of the linear regression of the natural logarithm of hopane normalized concentrations over time for each analyte in which sufficient data were available (e.g., values > reporting limit (RL): *n*-alkanes = 416.7 ng L^–1^ and PACs = 208.3 ng L^–1^). Regressions included 3 replicates for each time point (*n* = 9 for the 6 field experiments, *n* = 6 for the lab experiments), except for a small number of analytes where the natural log of the ratio could not be calculated for one sample (*n* = 8 or 5).

For the field experiments, regressions were conducted separately for live and killed treatments. When regression slopes were determined to be significantly different from zero, a *t* test was used to determine whether the slope for the live incubation was significantly different than the slope from the killed control. If this test was significant, then the difference in the slopes was considered to be the initial biodegradation rate constant. The regression slope based on the killed control incubations was considered to be the initial abiotic degradation rate constant. When slopes were not significantly different, the slope of the killed control incubations was considered to be the initial abiotic degradation rate constant, and no biodegradation occurred.

For the light and dark experiments, regressions were conducted for each of the light treatments, and slopes were compared between light and dark incubations. Where there was a significant difference, the difference in the slopes between the light and dark incubations was considered to be the initial photodegradation rate constant, while the dark incubation represented all other abiotic degradation processes. Regressions were conducted using MS Excel and the linest function.

### Percent Losses

Losses of compounds were calculated as the % difference between the average hopane normalized concentrations at the beginning and end of the experiments. Losses due to biodegradation were estimated as the difference between losses in the live incubations and the killed controls. For the light–dark experiments, photodegradation losses were calculated by the difference between light and dark incubations. Comparisons of initial degradation rate constants and losses among stations were conducted using the Kruskal–Wallis test in R.^41^ Post hoc analysis used the Dunn’s test in the dunn.test package^42^ with the Holm adjustment.

## Results and Discussion

The six field experiments represented a range of different environmental conditions (Table S1). Stn01 was warm and fresh, with high nitrate, ammonium, and silicate concentrations but low phosphate concentrations. The abundance of prokaryotes was highest at Stn01, with lower concentrations of chl *a* compared to some other stations. Stn11 was collected where deepwater upwells from the Laurentian Trench near the confluence of the St. Lawrence and Saguenay Rivers and was cold with high nutrients and low biomass. Located in the middle of the St. Lawrence River near Rimouski, QC, Canada, Stn20 had high chl *a* concentrations and lower nutrients due to a phytoplankton bloom. Water was cooler at Stn20 than that at the north Atlantic stations but warmer than that at Stn11 with slightly lower salinity. The three north Atlantic stations were similar in terms of temperature and salinity, with all three have low nutrient concentrations. Biomass was lowest at CGR11 and higher at the two coastal stations. All samples, regardless of location, had similar concentrations of TOC, with concentrations of 10–12 mg mL^–1^.

Heidrun is a medium crude oil with a density of 0.9064 g mL^–1^ and a viscosity of 40.5533 cSt at 25 °C. Analysis of the oil via thin-layer liquid chromatography and FID^43^ determined that saturates contributed to 37.84% (±1.05) of the oil, with aromatics representing 40.22% (±1.54) and the remainder composed of resins and asphaltenes at 20.08% (±1.88) and 1.85% (±0.26), respectively. Most of the oil compounds were not measured by the GC-MS technique used in this study, with the *n*-alkanes from C_13_–C_35_ representing 1.2% of the oil by mass and the quantified PACs representing 1.3%. The addition of 20 μL of Heidrun to the 120 mL of seawater in these experiments resulted in concentrations of approximately 150 ppm. Because of the error associated with pipetting small volumes of viscous fluids, slightly different amounts of oil were added to each jar, necessitating the use of hopane normalized ratios during analysis.

The amount of abiotic degradation and biodegradation observed varied among the stations. In total, 23 *n*-alkanes and 40 PACs were analyzed for each station (Tables S2–S7). Biodegradation at stations other than Stn01 was rarely observed (Table S8). At Stn01, initial biodegradation rate constants could be calculated for 17 *n*-alkanes and 30 PACs. The only other station where initial biodegradation rate constants could be calculated for more than one or two compounds was Stn20, for 2 *n*-alkanes and 8 PACs. Stn01 was the warmest station, with temperatures >20 °C, which have been shown to increase biodegradation rates.^44,45^ Stn01 also had high abundances of prokaryotes, the main drivers of biodegradation.^46,47^ The station was situated near Quebec City, QC, Canada, and downstream from Montreal, QC, Canada, both of which have large ports. The St. Lawrence Seaway is an active transportation corridor, and it might be expected that microbial communities in this region would be previously exposed to oil and may be primed to respond when oil is spilled.^48^ However, this was not observed for other stations, including CGR01 in Placentia Bay, NL, Canada, near the Port of Argentia, which includes a ferry terminal, or CGR22 in Chedabucto Bay, NS, Canada, at the entrance to the Strait of Canso, which provides access to the St. Lawrence Seaway. Chedabucto Bay is also where the SS *Arrow* sank in 1970, releasing over 100,000 barrels of Bunker C fuel. Additional oil was removed from the wreck in 2015; however, small releases of oil are still occasionally observed.^49^ The effectiveness of priming may be restricted by other environmental factors. O_2_ was not limiting in these experiments (Table S9), but temperature and nutrients may have reduced the microbial response. CGR01 and CGR22 were cooler with low concentrations of inorganic nutrients and high concentrations of chl *a* compared to Stn01. High losses due to biodegradation at Stn01 suggest that temperature and nutrient availability, rather than previous exposure to oil, may be driving the rapid response. Longer lag times for biodegradation may occur at the other stations with lower temperatures,^50–52^ and these experiments may not have been long enough to detect biodegradation at these stations. One possible example of priming may be Stn20, where sequencing data (not shown) suggest that cyanobacteria from the family Nostocaceae were in high abundance. One of the *n*-alkanes for which an initial biodegradation rate constant could be calculated at Stn20 was C_17_, which is an alkane produced by cyanobacteria.^53^ The initial biodegradation rate constants calculated across the six experiments were similar to those reported in studies covering a range of geographical locations and temperatures for both *n*-alkanes and PACs.^16,45,50^ While estimates are similar, an important caveat of the initial rate constants reported here is that they represent an initial estimate based only on 3 time points. This has limitations since it is difficult to determine and assess the correct reaction order from simple kinetics. Additional time points with longer incubations could result in different degradation rate constants.

Although initial biodegradation rate constants could not be calculated for most of the compounds from most stations, losses due to biodegradation could be estimated by the difference between 0 and 72 h (Figure 2). Losses were detected for all compounds at Stn01 and for all but 5 compounds at Stn20, although the % losses were lower at Stn20, possibly due to lower temperatures compared to those at Stn01. Few compounds had detectable losses due to biodegradation at Stn11 and the Atlantic coast stations. While 7–9 *n*-alkanes had losses due to biodegradation at these 4 stations, fewer PACs showed losses. Low temperatures at Stn11 might be expected to slow biodegradation and the microbial response; however, the Atlantic stations were much warmer, and a shorter lag period might be expected. The slower response at these stations could be due to low nutrient availability or an initial microbial community that lacked the ability to degrade complex organic compounds.^2,32,33^

**Figure 2:**
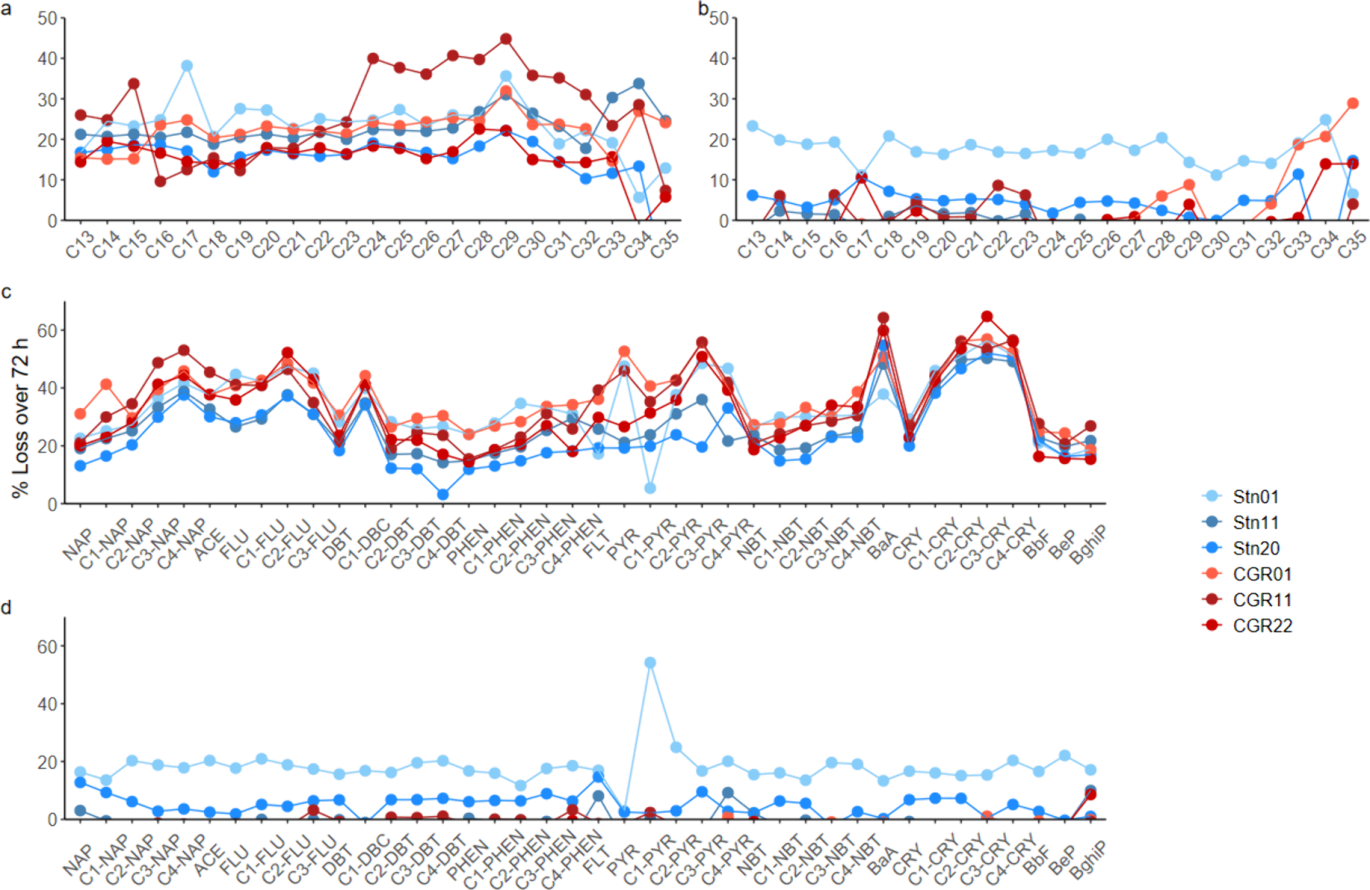
Estimated average % loss for the 23 *n*-alkanes and 40 PACs due to abiotic processes or biodegradation in the six experiments: (a) abiotic degradation and (b) biodegradation of *n*-alkanes by chain length and (c) abiotic degradation and (d) biodegradation of PACs. Abbreviations for PACs: NAP, naphthalene; ACE, acenaphthene; FLU, fluorene; DBT, dibenzothiophene; PHEN, phenanthrene; FLT, fluoranthene; PYR, pyrene; NBT, naphthobenzothiophene; BaA, benz­[a]­anthracene; CRY, chrysene; BbF, benzo­[b]­fluoranthene; BeP, benzo­[e]­pyrene; and BghiP, benzo­[ghi]­perylene. C1, C2, C3, and C4 indicate methyl, dimethyl, trimethyl, and tetramethyl alkylated homologues, respectively.

Somewhat surprisingly, abiotic degradation and losses in the killed control incubations were relatively high in these experiments. Abiotic losses were detected for almost all PACs, and a large number of *n*-alkanes were detected for all stations (Figure 2). Abiotic degradation rate constants for individual analytes were within a small range, with more variation in rate constants for PACs compared to those for *n*-alkanes (Table 2, Table S10). Few degradation rate constants could be calculated for *n*-alkanes at CGR01 and CGR22, while rate constants could be calculated for most *n*-alkanes at the other stations. Nitrate was low at these stations, which could have limited indirect oxidation,^21–23^ but it was also low at CGR11, where the mean rate constant was highest. Overall abiotic degradation rate constants for PACs were higher than those calculated for *n*-alkanes. There was no relationship between high *n*-alkane rate constants and high PAC rate constants. Abiotic degradation rate constants for PACs were highest at CGR01 but lowest at Stn11. This disconnect suggests that different factors affect the degradation of PACs compared to *n*-alkanes. Overall, there was no obvious pattern that indicated which environmental factors might have the largest influence on the rate of abiotic losses observed at each station.

**Table 2:** Initial Abiotic Degradation Rate Constants Calculated for the Six Individual Stations^a^

	*n*-Alkanes			PACs		
Experiment	Range (d^–1^)	Mean (d^–1^)	*n*	Range (d^–1^)	Mean (d^–1^)	*n*
Stn01	0.06–0.16	0.10 (0.02)	20	0.08–0.28	0.15 (0.05)	37
Stn11	0.05–0.14	0.09 (0.02)	23	0.05–0.23	0.11 (0.05)	39
Stn20	0.04–0.08	0.06 (0.01)	17	0.05–0.26	0.11 (0.06)	31
CGR01		0.09	1	0.10–0.30	0.19 (0.06)	27
CGR11	0.04–0.19	0.12 (0.04)	17	0.05–0.34	0.15 (0.07)	39
CGR22	0.06–0.09	0.07 (0.01)	5	0.07–0.35	0.16 (0.07)	31

a Means and standard deviations are shown, with *n* indicating the number of analytes included. Rate constants for individual compounds are given in Table S10.

Although only visible light was included in these incubations, photodegradation was identified as a potential contributor to abiotic degradation in these experiments. To quantify potential photodegradation, additional experiments compared degradation rates over 72 h in light incubations to those in the dark in the presence of HgCl_2_ using both seawater and Milli-Q. Milli-Q in the absence of HgCl_2_ tested the potential interaction between light and HgCl_2_. After extraction of samples for GC-MS analysis, differences in color extracts were observed between the light and dark incubations. This was quantified by measuring the absorbance of the extracts, which showed a clear decrease in absorbance between 300 and 425 nm in samples incubated in the light compared to those incubated in the dark (Figure 3). The clear loss of absorbance in the UVA range in the light incubations indicates a loss of PACs, corroborating the GC-MS analyses.

**Figure 3:**
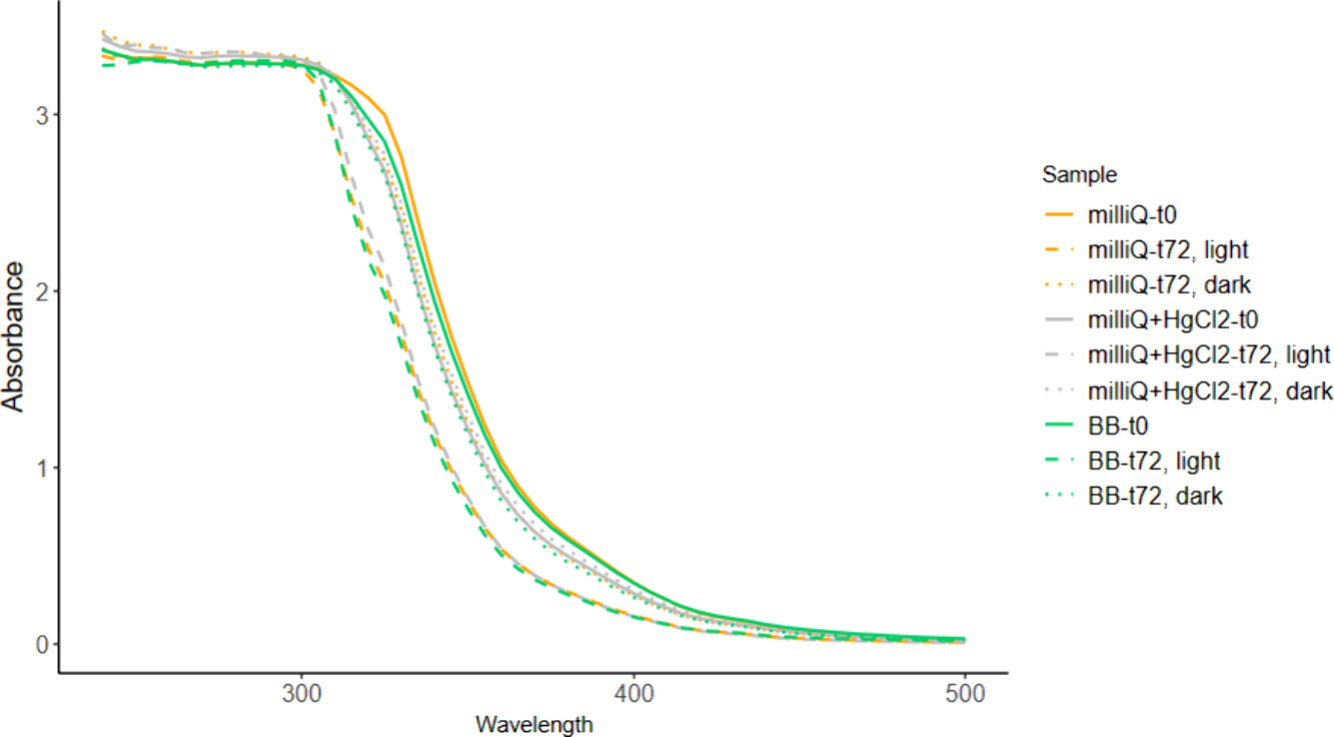
Absorbance of the extracts from the Bedford Basin and Milli-Q experiments from 240 to 500 nm. Each line represents the mean of 3 extracts at 0 h (solid lines), after incubation in the light for 72 h (dashed lines), and after incubation in the dark for 72 h (dotted lines).

Few *n*-alkanes or PACs were found to degrade in the dark in seawater with HgCl_2_ (Table S11), while initial degradation rate constants could be calculated for 5 *n*-alkanes and 25 PACs when incubated in the light (Table 3 and Table S14). In the Milli-Q experiments, the patterns with and without HgCl_2_ were similar (Tables S12 and S13), with degradation rate constants able to be calculated for most of the compounds after incubation in the light (Table 3 and Table S14). In the dark incubations with Milli-Q, the few calculated degradation rate constants were negative, likely reflecting variability across replicates where there was little difference in the hopane normalized concentrations at 0 and 72 h.

**Table 3:** Initial Degradation Rate Constants for Photodegradation and All Other Abiotic Degradation Processes for *n*-Alkanes and PACs^a^

	Photodegradation				Other Abiotic Degradation			
	*n*-Alkanes (d^–1^)	*n*	PACs (d^–1^)	*n*	*n*-Alkanes (d^–1^)	*n*	PACs (d^–1^)	*n*
BB + HgCl_2_	0.12 (0.03)	5	0.16 (0.05)	25	0.06	1	0.04	1
Milli-Q	0.11 (0.02)	21	0.12 (0.03)	40	–0.02 (0.00)	2	–0.08	1
Milli-Q + HgCl_2_	0.08 (0.02)	19	0.10 (0.05)	40			–0.04 (0.11)	2

a Means are given with standard deviations, and *n* represents the number of analytes included in the calculation. Individual rate constants are given in Table S14.

Based on the average difference in hopane normalized concentrations between 0 and 72 h with the Bedford Basin water, *n*-alkane losses due to other abiotic processes were approximately half the losses attributed to photodegradation (Figure 4). There was a larger difference between PAC losses in the dark and light, with photodegradation removing ∼3 times the PACs as other abiotic factors. In Milli-Q, losses of both *n*-alkanes and PACs in the dark varied by compound, with overall averages suggesting no losses over the 72 h with or without HgCl_2_.

**Figure 4:**
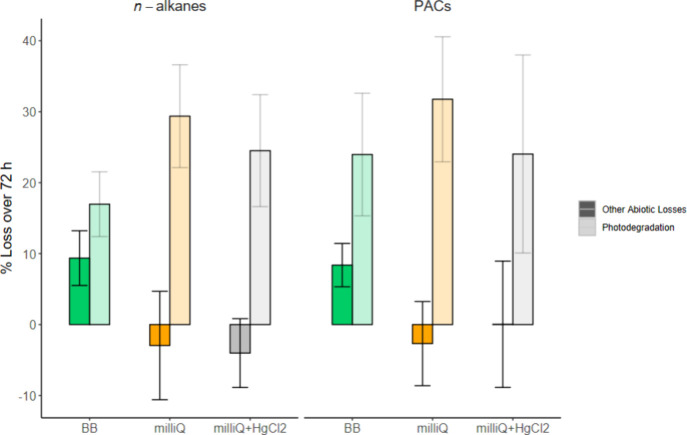
Average percent losses over 72 h due to photodegradation only compared to all other abiotic losses during the Bedford Basin and Milli-Q experiments. Losses due to photodegradation are shown in the lighter bars, with all other abiotic losses in the darker bars and colors indicating the experiment. Error bars are standard deviations of *n* = 23 for *n*-alkanes and *n* = 43 for PACs.

One cause for the abiotic losses in the dark could be due to volatilization of the oil when the jars were uncapped to add DCM.^55^ This would be expected to affect the shorter chain *n*-alkanes and 2-ring PACs more than the other compounds,^56^ which was not observed in these experiments. This, along with the lack of losses in Milli-Q, suggests volatilization was not responsible for the abiotic losses.

Another mechanism for abiotic losses in the dark incubations could be due to the presence of humic acids, NO_3_^–^, SO_4_^2–^, cations such as Fe^2+^ or Mn^2+^, and minerals, all of which are present in rivers and seawater and all of which can participate in redox reactions resulting in the degradation of hydrocarbons.^17,57–61^ This mechanism is supported by the lack of detectable losses in the dark Milli-Q incubations, but the current data are not definitive. Similar losses with HgCl_2_ have previously been observed in natural samples. In an experiment in which crude oil was added to soil and incubated at room temperature, 4.7% of the quantified *n*-alkanes were degraded in a HgCl_2_ control,^54^ similar to the loss observed in this study.

Increased losses in the dark for BB could be due to differences in extraction efficiencies in the seawater compared to the pure water rather than due to degradation. This could be due to the binding of oil to organic matter in the samples during the incubation and decreased extraction efficiencies at 72 h compared to the pure water. The relatively low TOC concentrations in the seawater should not have interfered with the DCM extraction of hydrocarbons. Comparisons of extraction recoveries for the deuterated standards between 0 and 72 h within an experiment (data not shown) revealed no significant differences, which indicated that the targeted compounds have been degraded.

In contrast to the observations in this study, Bacosa et al.^16^ observed essentially no losses in their dark control incubations of seawater with oil or chemically dispersed oil. Unlike this experiment, where HgCl_2_ was used to kill the organisms in the water, Bacosa et al. filtered their control samples, removing the microbes responsible for biodegradation but also likely removing some of the organic matter that may contribute to the oxidation of oil compounds.^22,23^ Some organisms, including some known to have oil degrading potential, such as *Marinomonas*, *Pseudomonas*, and *Alteromonas*, may survive high concentrations of mercury;^62^ however, essentially no degradation of volatile hydrocarbons was observed after incubation at 30 °C for several days after the addition of HgCl_2_ to ∼4 mM, indicating this is an effective biocide.^63,64^ As this concentration was much higher than that used in this study, plating of cells was used to test the effectiveness of HgCl_2_ in inhibiting microbial growth. Cells in the presence of 180 μM HgCl_2_ did not grow during incubation at 22 °C for 72 h (Table S15), suggesting that biological activity in the killed incubations was effectively inhibited in this study and that biodegradation is unlikely responsible for losses in the dark.

There were slightly higher losses of *n*-alkanes and PACs due to photodegradation in the untreated Milli-Q compared to Milli-Q with HgCl_2_ (Figure 4). This would suggest that HgCl_2_ could inhibit photodegradation, resulting in an underestimation of photodegradation and an overestimation of biodegradation in the field samples. Losses due to photodegradation in Milli-Q also exceeded those in Bedford Basin seawater for *n*-alkanes, while losses of PACs due to photodegradation were similar between the Bedford Basin and Milli-Q incubations. This would suggest that the organic matter and other chemicals in seawater may inhibit indirect photodegradation of *n*-alkanes, while direct photodegradation of PACs is not affected.^65,66^

Initial abiotic degradation rate constants for all experiments were compared using the Kruskal–Wallis test followed by a Dunn’s test (Figure 5). These were generally higher for PACs compared to *n*-alkanes, although the difference was small for the Milli-Q experiments. Compared to the initial rate constants measured in Milli-Q with HgCl_2_, all stations had similar or higher degradation rate constants, indicating a potential positive effect of photocatalysts.^21–23^ The highest initial rate constants for abiotic degradation of *n*-alkanes were observed for Milli-Q and stations representing a range of temperatures, salinities, nutrient concentrations, and biomass, suggesting none of these factors are the main drivers of abiotic degradation of *n*-alkanes. While TOC concentrations were relatively consistent across all samples, the composition of organic matter (e.g., humic acids vs proteins) is likely important in mediating the indirect oxidation of *n*-alkanes.^67,68^

**Figure 5:**
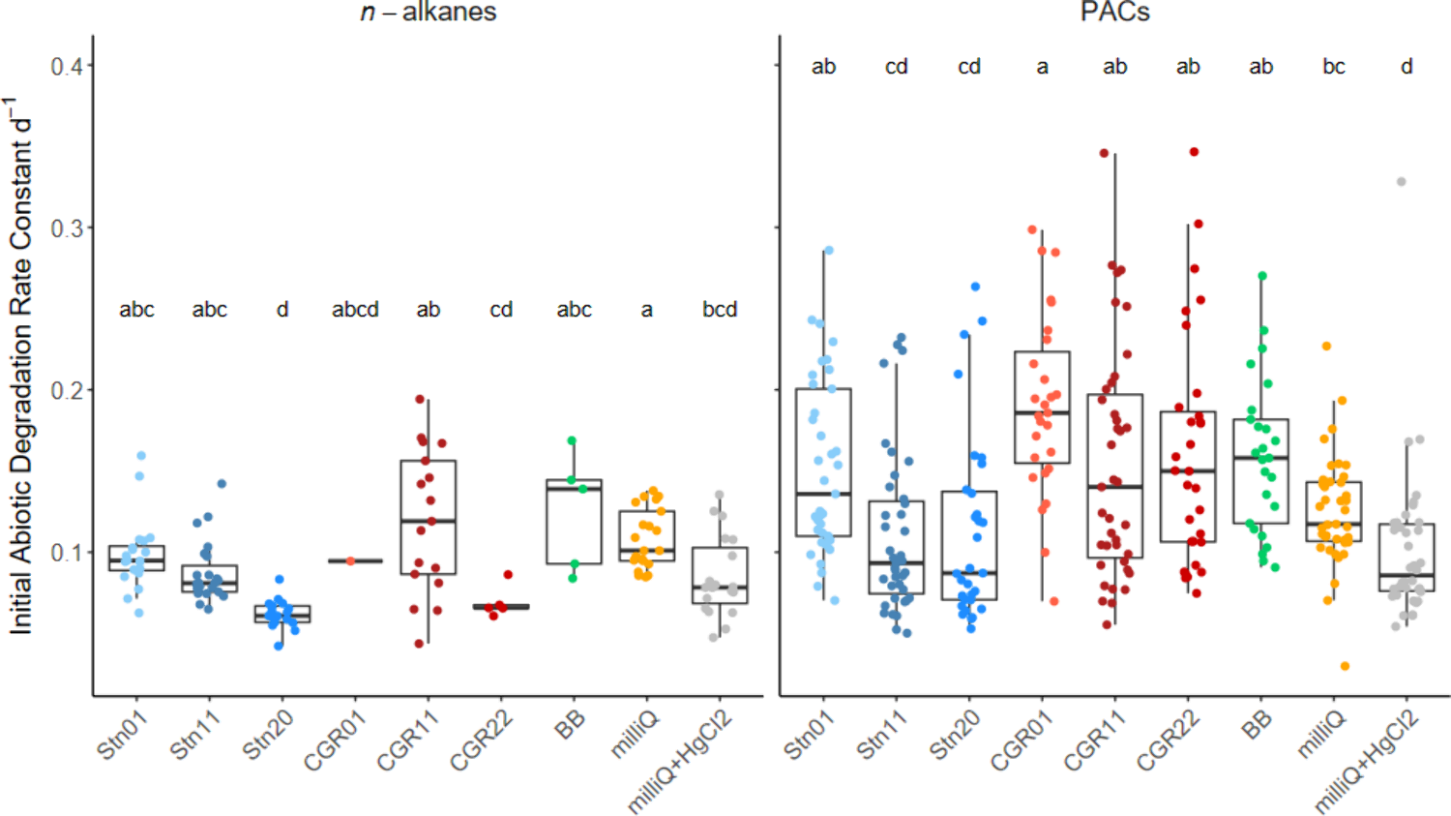
Comparison of the estimated abiotic degradation rates for each station, including the Bedford Basin and Milli-Q experiments. Rates include mainly photodegradation, as well as additional abiotic losses. Stations that share the same letter are not significantly different from each other based on the Dunn’s test (*p*_adjusted_ < 0.05).

Most stations had significantly higher initial rate constants for abiotic degradation of PACs compared to the rates in Milli-Q with HgCl_2_. The exceptions were for Stn11 and Stn20, the coldest stations (<10 °C) with relatively low turbidity, although they differed in inorganic nutrient concentrations, salinity, and biomass. In contrast to the observations for the *n*-alkanes, the initial abiotic degradation rates of the PACs appear to be affected by temperature to some extent.^28,59^ As Stn01 did not have the highest initial rate constants, it is likely that interactions between factors affect the abiotic degradation of oil compounds. Initial abiotic degradation rate constants calculated at CGR01, which had low inorganic nutrient concentrations but relatively high biomass, were greater than those measured in Milli-Q with similar incubation temperatures. This could indicate some indirect degradation of the PACs. While not directly comparable due to differences in the methods used and limitations of the initial rate constants calculated here, the initial abiotic degradation rate constants in this study are similar to the photooxidation rate constants calculated for the Gulf of Mexico, where temperatures were generally more similar to those of Stn01 than those of the other stations in this study.^16^

It is generally accepted that ring number and the presence of alkyl groups will affect photodegradation rates of PACs.^65,66^ Pooling the data from all experiments, differences in initial abiotic degradation rate constants were observed after separating PACs into parent and alkylated compounds and grouping them by ring number (Figure 6). For a given ring number, the alkylated compounds tended to degrade abiotically faster than the unsubstituted PACs, although there was variability in the measured rate constants within each station. Abiotic degradation of 3-ring PACs was slower than that of 4-ring PACs as has previously been observed,^65,66^ although faster photooxidation of smaller ring PACs compared to PACs with more rings has also been documented.^16^ Lower ring number is generally associated with higher acute toxicity,^10^ but photooxidized products may have even higher toxicities.^7,9,18^

**Figure 6:**
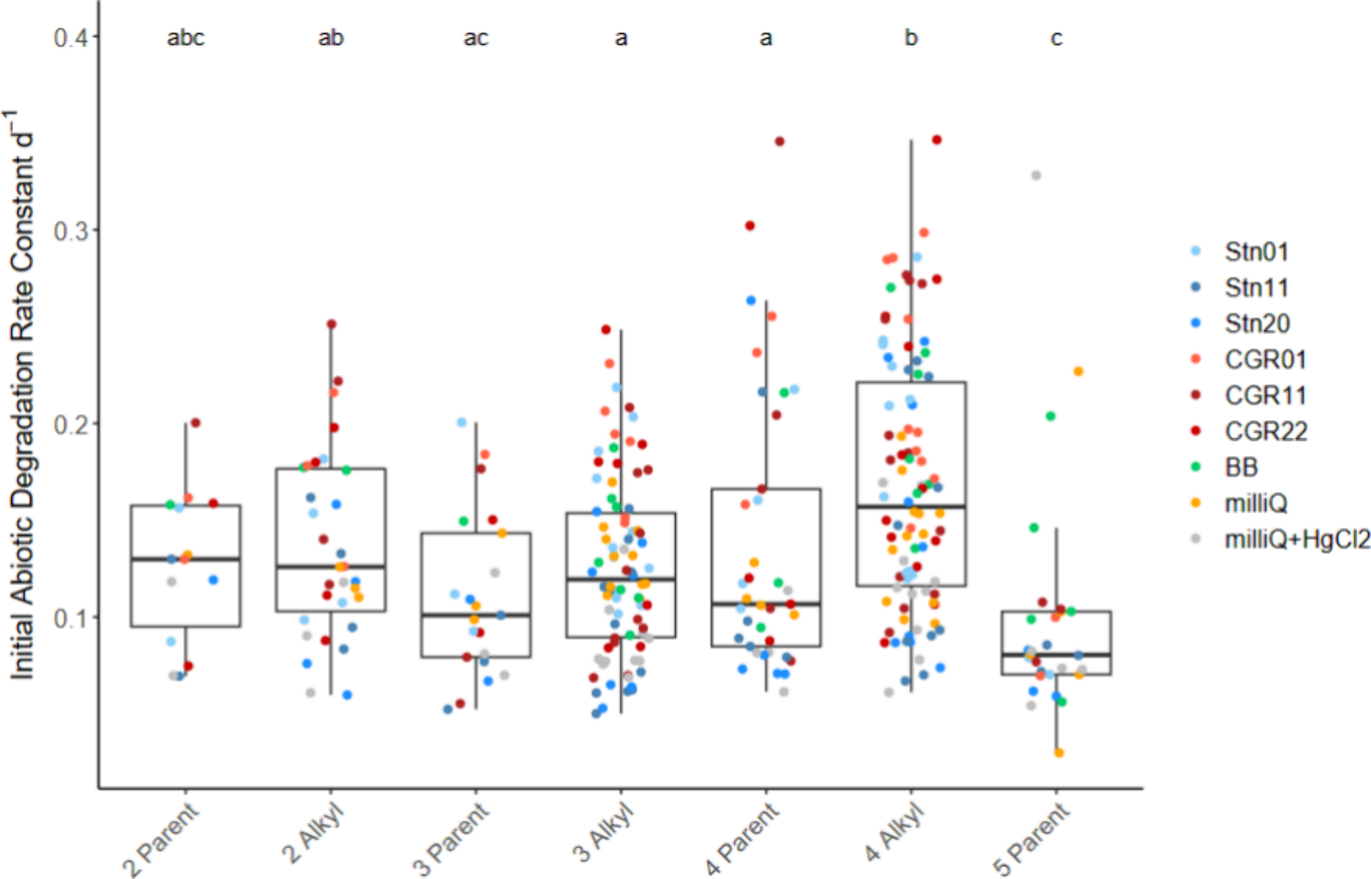
Comparison of PAC abiotic degradation rates pooling all experiments separated by ring number and the presence of alkyl groups. PAC groups that share the same letter are not significantly different from each other based on the Dunn’s test (*p*_adjusted_ < 0.05). Individual data points are colored by station or experiment.

While experiments conducted with pure water or irradiation of films of oil can provide insights into the mechanisms of photodegradation of oil,^69–71^ this study demonstrates that environmental conditions will affect the rates and impacts of photodegradation in the field.^20^ Interactions among factors may result in higher or lower abiotic losses in the field compared to those in pure water experiments. While preliminary, these experiments clearly demonstrate that photodegradation is an important process in the early stages of a spill of a medium crude oil, whether mediated by UV or visible light. Further investigation is needed to elucidate the mechanisms underlying visible light mediated photodegradation. Biodegradation can be significant under the right environmental conditions but may have a longer lag period. Photodegradation early in a spill may be critical in driving the development of the microbial community and could explain why diverse communities develop in surface waters in response to oil.^19,67^ During the *Deepwater Horizon* incident, photodegradation was not a factor in the deepwater dispersed oil plume, and the microbial community became dominated by a few strains of microbes.^72,73^ In contrast, in surface spills, diverse communities develop, with various relative abundances and identities of biodegrading microbes.^32,74,75^ These communities reflect the distinct community present before the introduction of oil; however, different extents of photodegradation of the oil early in the spill and the broad range of oxidized products produced could select for a diverse oil degrading community. This study highlights the need to quantify abiotic degradation rates under environmentally relevant conditions in order to incorporate these rates into models and better predict the fate and behavior of oil following a spill.

## Supplementary Material



## Notes

Initial physical and chemical conditions of the sampled water from the six stations, regression statistics for the six experiments, associated biodegradation rate constants and abiotic degradation rate constants, O_2_ concentrations, regression statistics for the Bedford Basin and Milli-Q experiments for individual analytes, estimated abiotic and photodegradation rate constants, and colony plate counts (PDF)
