# Supplementary material for: Photodegradation Driven by Visible Light Exceeds Biodegradation across a Range of Environmental Conditions during the Initial Hours after an Oil Spill
Source: Environ Sci Technol. 2025 Sep 26;59(39):21312–23. doi: 10.1021/acs.est.5c06941 (PMC12509326; doi:10.1021/acs.est.5c06941)
Supplement: Supplementary file 1 [file es5c06941_si_001.pdf]

Photodegradation driven by visible light exceeds biodegradation across a range of environmental conditions during the initial hours after an oil spill

Alice C. Ortmann, Brian Robinson, Ho-Yin Poon and Thomas L. King*

Bedford Institute of Oceanography, Fisheries and Oceans Canada, 1 Challenger Drive, Dartmouth, NS, Canada B2Y 4A2

*Corresponding author:

Alice.Ortmann@dfo-mpo.gc.ca

902-943-9531

Number of Pages: 20

Number of Supplemental Tables: 15

Table S1. Physical, chemical and biological parameters associated with the water used for the six experiments. Values are shown as means with standard deviations in parentheses (n=9), except where derived from the CTD data where the value represents the measurement collected when the samples were collected and do not have associated errors.

**Station**	**Stn01**	**Stn11**	**Stn20**	**CGR01**	**CGR11**	**CGR22**
**Temperature (°C)**	23.8	2.98	8.67	14.55	16.72	17.22
**Salinity**	0.12	30.83	27.93	31.82	31.6	29.62
**Dissolved O_2 (mL L^{-1})**	5.94	5.58	7.07	5.01	5.9	6.05
**Turbidity (NTU)***	6.45	1.52	1.58	—	—	—
**Backscatter coefficient (m^{-1})***	—	—	—	0.0039	0.0018	0.0036
**TOC (mg L^{-1})**	11.47 (0.57)	15.17 (6.84)	14.73 (5.01)	10.95 (0.16)	11.29 (0.25)	10.82 (0.14)
**NO_3^- (µM)**	11.07 (0.23)	13.62 (0.03)	1.84 (0.57)	0.12 (0.04)	0.13 (0.06)	0.11 (0.00)
**NH_4^+ (µM)**	0.84 (0.14)	0.77 (0.04)	0.12 (0.02)	0.12 (0.05)	0.36 (0.11)	0.27 (0.09)
**PO_4^{-3} (µM)**	0.17 (0.03)	1.14 (0.07)	0.45 (0.06)	0.26 (0.04)	0.19 (0.04)	0.08 (0.01)
**Silicate (µM)**	16.46 (2.17)	15.2 (0.17)	5.1 (0.52)	0.57 (0.10)	0.5 (0.03)	1.24 (0.05)
**Chl a (µg L^{-1})**	1.92 (0.66)	0.13 (0.02)	7.98 (0.16)	3.62 (0.43)	0.41 (0.72)	6.06 (0.49)
**Prokaryotes (10^6 cells mL^{-1})**	5.75 (0.50)	0.79 (0.16)	2.49 (0.40)	2.38 (0.06)	1.00 (0.15)	3.92 (0.32)

*The turbidity meter in 2019 reported data as NTU, while the instrument in 2020 reported data in m^{-1} sr^{-1}, which was corrected to backscatter coefficients. The two values are not directly comparable.

Table S2 Linear regression statistics for the live and killed control incubations from Stn01. For each treatment, a t-test was conducted to determine if the slope was significantly different from zero. A second t-test compared the slope from the killed control incubations to those of the live incubations. The estimated rate constant is calculated for abiotic degradation or biodegradation.

Stn01	Linear Regressions																		t-test for Significant difference between slopes					Rate constant (h-1)				Rate constant (d-1)	
	Live									Killed Control																			
Analyte	Intercept	Slope	SlopeSE	df	R2	Fstat	t-value	p-value		Intercept	Slope	SlopeSE	df	R2	Fstat	t-value	p-value		Error	t statistic	df	p-value		Abiotic	Live	Biodegradation		Abiotic	Biodegrada
C13	2.76	-0.01	0.00	7	0.94	108.63	-10.42	0.00		2.61	0.00	0.00	7	0.50	6.95	-2.64	0.03		0.00	3.75	10	0.00		0.00	-0.01	0.00		0.06	0.11
C14	2.93	-0.01	0.00	7	0.92	84.70	-9.20	0.00		2.79	0.00	0.00	7	0.50	7.11	-2.67	0.03		0.00	2.42	10	0.04		0.00	-0.01	0.00		0.09	0.10
C15	2.93	-0.01	0.00	7	0.94	107.97	-10.39	0.00		2.82	0.00	0.00	7	0.48	6.44	-2.54	0.04		0.00	2.34	10	0.04		0.00	-0.01	0.00		0.09	0.09
C16	2.73	-0.01	0.00	7	0.95	134.01	-11.58	0.00		2.59	0.00	0.00	7	0.56	8.96	-2.99	0.02		0.00	2.75	10	0.02		0.00	-0.01	0.00		0.09	0.10
C17	2.82	-0.01	0.00	7	0.75	20.48	-4.53	0.00		2.80	-0.01	0.00	7	0.63	11.72	-3.42	0.01		0.00	0.99	10	0.35		-0.01	-0.01	na		0.16	
C18	2.49	-0.01	0.00	7	0.95	130.29	-11.41	0.00		2.33	0.00	0.00	7	0.50	6.96	-2.64	0.03		0.00	3.04	10	0.01		0.00	-0.01	0.00		0.08	0.10
C19	2.42	-0.01	0.00	7	0.93	100.46	-10.02	0.00		2.31	0.00	0.00	7	0.62	11.49	-3.39	0.01		0.00	2.29	10	0.04		0.00	-0.01	0.00		0.11	0.09
C20	2.04	-0.01	0.00	7	0.97	200.89	-14.17	0.00		1.92	0.00	0.00	7	0.58	9.64	-3.10	0.02		0.00	2.25	10	0.05		0.00	-0.01	0.00		0.11	0.08
C21	2.01	-0.01	0.00	7	0.94	108.59	-10.42	0.00		1.88	0.00	0.00	7	0.58	9.72	-3.12	0.02		0.00	2.76	10	0.02		0.00	-0.01	0.00		0.09	0.09
C22	2.00	-0.01	0.00	7	0.95	129.64	-11.39	0.00		1.89	0.00	0.00	7	0.66	13.73	-3.71	0.01		0.00	2.69	10	0.02		0.00	-0.01	0.00		0.10	0.08
C23	1.96	-0.01	0.00	7	0.95	122.45	-11.07	0.00		1.86	0.00	0.00	7	0.65	13.19	-3.63	0.01		0.00	2.65	10	0.02		0.00	-0.01	0.00		0.09	0.08
C24	2.00	-0.01	0.00	7	0.95	121.24	-11.01	0.00		1.89	0.00	0.00	7	0.59	10.15	-3.19	0.02		0.00	2.53	10	0.03		0.00	-0.01	0.00		0.10	0.09
C25	2.01	-0.01	0.00	7	0.96	170.33	-13.05	0.00		1.91	0.00	0.00	7	0.69	15.72	-3.97	0.01		0.00	2.73	10	0.02		0.00	-0.01	0.00		0.11	0.08
C26	2.56	-0.01	0.00	7	0.94	103.14	-10.16	0.00		2.43	0.00	0.00	7	0.58	9.87	-3.14	0.02		0.00	2.90	10	0.02		0.00	-0.01	0.00		0.09	0.10
C27	3.05	-0.01	0.00	7	0.94	118.06	-10.87	0.00		2.94	0.00	0.00	7	0.66	13.81	-3.72	0.01		0.00	2.64	10	0.02		0.00	-0.01	0.00		0.10	0.09
C28	2.55	-0.01	0.00	7	0.94	113.79	-10.67	0.00		2.42	0.00	0.00	7	0.59	9.98	-3.16	0.02		0.00	2.84	10	0.02		0.00	-0.01	0.00		0.10	0.11
C29	2.48	-0.01	0.00	7	0.93	97.72	-9.89	0.00		2.40	-0.01	0.00	7	0.75	20.49	-4.53	0.00		0.00	2.05	10	0.07		-0.01	-0.01	na		0.15	
C30	0.84	-0.01	0.00	7	0.97	234.96	-15.33	0.00		0.76	0.00	0.00	7	0.71	16.76	-4.09	0.00		0.00	1.99	10	0.08		0.00	-0.01	na		0.10	
C31	0.83	-0.01	0.00	7	0.91	73.72	-8.59	0.00		0.73	0.00	0.00	7	0.46	6.02	-2.45	0.04		0.00	1.95	10	0.08		0.00	-0.01	na		0.07	
C32	0.60	-0.01	0.00	7	0.92	85.40	-9.24	0.00		0.49	0.00	0.00	7	0.58	9.69	-3.11	0.02		0.00	2.02	10	0.07		0.00	-0.01	na		0.08	
C33	0.55	-0.01	0.00	7	0.95	131.67	-11.47	0.00		0.40	0.00	0.00	7	0.40	4.60	-2.14	0.07							na	-0.01	-0.01			0.16
C34	0.31	-0.01	0.00	7	0.89	59.24	-7.70	0.00		0.13	0.00	0.00	7	0.06	0.47	-0.69	0.51							na	-0.01	-0.01			0.12
C35	-0.06	0.00	0.00	7	0.38	4.28	-2.07	0.08		-0.09	0.00	0.00	6	0.18	1.30	-1.14	0.30							na	na	na			
naphthalene	2.28	-0.01	0.00	7	0.87	48.93	-7.00	0.00		2.19	0.00	0.00	7	0.66	13.43	-3.66	0.01		0.00	2.31	10	0.04		0.00	-0.01	0.00		0.09	0.08
methylnaphthalene	3.14	-0.01	0.00	7	0.83	34.05	-5.83	0.00		3.07	0.00	0.00	7	0.50	7.13	-2.67	0.03		0.00	1.38	10	0.20		0.00	-0.01	na		0.10	
dimethylnaphthalene	3.92	-0.01	0.00	7	0.94	115.58	-10.75	0.00		3.77	0.00	0.00	7	0.68	14.97	-3.87	0.01		0.00	3.12	10	0.01		0.00	-0.01	0.00		0.11	0.11
trimethylnaphthalene	3.90	-0.01	0.00	7	0.97	217.58	-14.75	0.00		3.74	-0.01	0.00	7	0.76	22.14	-4.71	0.00		0.00	3.08	10	0.01		-0.01	-0.01	0.00		0.15	0.12
tetramethylnaphthalene	3.48	-0.01	0.00	7	0.97	207.10	-14.39	0.00		3.32	-0.01	0.00	7	0.78	25.20	-5.02	0.00		0.00	2.85	10	0.02		-0.01	-0.01	0.00		0.18	0.12
acenaphthene	-0.51	-0.01	0.00	7	0.97	270.91	-16.46	0.00		-0.64	-0.01	0.00	7	0.69	15.22	-3.90	0.01		0.00	3.00	10	0.01		-0.01	-0.01	-0.01		0.16	0.13
fluorene	1.43	-0.01	0.00	7	0.96	191.64	-13.84	0.00		1.29	-0.01	0.00	7	0.74	20.29	-4.50	0.00		0.00	2.47	10	0.03		-0.01	-0.01	-0.01		0.20	0.12
methylfluorene	2.01	-0.01	0.00	7	0.98	310.53	-17.62	0.00		1.88	-0.01	0.00	7	0.78	25.50	-5.05	0.00		0.00	3.53	10	0.01		-0.01	-0.01	-0.01		0.19	0.15
dimethylfluorene	2.20	-0.02	0.00	7	0.98	302.06	-17.38	0.00		2.08	-0.01	0.00	7	0.79	26.77	-5.17	0.00		0.00	3.03	10	0.01		-0.01	-0.02	-0.01		0.22	0.14
trimethylfluorene	2.22	-0.01	0.00	7	0.96	174.32	-13.20	0.00		2.10	-0.01	0.00	7	0.79	25.99	-5.10	0.00		0.00	2.63	10	0.03		-0.01	-0.01	-0.01		0.20	0.12
dibenzothiophene	0.93	-0.01	0.00	7	0.95	135.92	-11.66	0.00		0.80	0.00	0.00	7	0.64	12.32	-3.51	0.01		0.00	2.23	10	0.05		0.00	-0.01	0.00		0.11	0.08
methyldibenzothiophene	2.76	-0.01	0.00	7	0.97	198.96	-14.11	0.00		2.59	-0.01	0.00	7	0.76	22.43	-4.74	0.00		0.00	2.59	10	0.03		-0.01	-0.01	0.00		0.17	0.11
dimethyldibenzothiophene	2.39	-0.01	0.00	7	0.96	173.36	-13.17	0.00		2.26	0.00	0.00	7	0.60	10.64	-3.26	0.01		0.00	2.16	10	0.06		0.00	-0.01	na		0.11	
trimethyldibenzothiophene	2.29	-0.01	0.00	7	0.94	111.29	-10.55	0.00		2.12	0.00	0.00	7	0.55	8.60	-2.93	0.02		0.00	2.51	10	0.03		0.00	-0.01	0.00		0.10	0.10
tetramethyldibenzothiophene	1.92	-0.01	0.00	7	0.95	131.06	-11.45	0.00		1.75	0.00	0.00	7	0.61	10.89	-3.30	0.01		0.00	2.82	10	0.02		0.00	-0.01	0.00		0.11	0.10
phenanthrene	1.97	-0.01	0.00	7	0.94	114.22	-10.69	0.00		1.84	0.00	0.00	7	0.54	8.35	-2.89	0.02		0.00	2.26	10	0.05		0.00	-0.01	0.00		0.09	0.08
methylphenanthrene	2.73	-0.01	0.00	7	0.96	165.77	-12.88	0.00		2.61	0.00	0.00	7	0.62	11.23	-3.35	0.01		0.00	2.27	10	0.05		0.00	-0.01	0.00		0.11	0.08
dimethylphenanthrene	3.01	-0.01	0.00	7	0.93	94.75	-9.73	0.00		2.92	-0.01	0.00	7	0.66	13.39	-3.66	0.01		0.00	1.41	10	0.19		-0.01	-0.01	na		0.14	
trimethylphenanthrene	2.86	-0.01	0.00	7	0.97	226.47	-15.05	0.00		2.72	-0.01	0.00	7	0.70	16.26	-4.03	0.00		0.00	2.68	10	0.02		-0.01	-0.01	0.00		0.14	0.10
tetramethylphenanthrene	2.40	-0.01	0.00	7	0.91	74.34	-8.62	0.00		2.25	-0.01	0.00	7	0.73	18.83	-4.34	0.00		0.00	2.57	10	0.03		-0.01	-0.01	0.00		0.13	0.10
fluoranthene	-0.67	-0.01	0.00	7	0.39	4.55	-2.13	0.07		-0.77	0.00	0.00	7	0.04	0.30	-0.55	0.60							na	na	na			
pyrene	0.01	-0.01	0.00	7	0.92	81.34	-9.02	0.00		0.03	-0.01	0.00	7	0.79	26.08	-5.11	0.00		0.00	0.34	10	0.74		-0.01	-0.01	na		0.22	
methylpyrene	1.41	-0.01	0.00	7	0.95	126.93	-11.27	0.00		0.67	0.00	0.01	7	0.03	0.20	0.44	0.67							na	-0.01	-0.01			0.30
dimethylpyrene	1.37	-0.01	0.00	7	0.95	146.09	-12.09	0.00		1.19	-0.01	0.00	7	0.66	13.37	-3.66	0.01		0.00	3.14	10	0.01		-0.01	-0.01	-0.01		0.16	0.16
trimethylpyrene	1.28	-0.01	0.00	7	0.97	201.24	-14.19	0.00		1.17	-0.01	0.00	7	0.74	19.72	-4.44	0.00		0.00	2.13	10	0.06		-0.01	-0.01	na		0.23	
tetramethylpyrene	2.04	-0.02	0.00	7	0.86	41.75	-6.46	0.00		1.89	-0.01	0.00	7	0.77	23.53	-4.85	0.00		0.00	2.29	10	0.04		-0.01	-0.02	-0.01		0.21	0.17
naphthobenzothiophene	-0.48	-0.01	0.00	7	0.96	152.82	-12.36	0.00		-0.60	0.00	0.00	7	0.63	11.94	-3.46	0.01		0.00	2.27	10	0.05		0.00	-0.01	0.00		0.10	0.08
methylnaphthobenzothiophene	1.00	-0.01	0.00	7	0.95	135.21	-11.63	0.00		0.87	0.00	0.00	7	0.62	11.44	-3.38	0.01		0.00	2.15	10	0.06		0.00	-0.01	na		0.12	
dimethylNBenzothiophene	1.28	-0.01	0.00	7	0.97	241.91	-15.55	0.00		1.15	-0.01	0.00	7	0.67	14.51	-3.81	0.01		0.00	2.03	10	0.07		-0.01	-0.01	na		0.12	
trimethylNbenzothiophene	1.14	-0.01	0.00	7	0.97	232.02	-15.23	0.00		0.97	-0.01	0.00	7	0.63	11.69	-3.42	0.01		0.00	2.79	10	0.02		-0.01	-0.01	0.00		0.12	0.11
tetramethylNbenzothiophene	0.67	-0.01	0.00	7	0.94	109.95	-10.49	0.00		0.53	-0.01	0.00	7	0.57	9.23	-3.04	0.02		0.00	2.27	10	0.05		-0.01	-0.01	0.00		0.12	0.10
benz[a]anthracene	-1.82	-0.01	0.00	7	0.92	83.06	-9.11	0.00		-1.87	-0.01	0.00	7	0.80	27.18	-5.21	0.00		0.00	1.92	10	0.08		-0.01	-0.01	na		0.16	
chrysene	-0.21	-0.01	0.00	7	0.96	176.93	-13.30	0.00		-0.32	0.00	0.00	7	0.63	12.15	-3.49	0.01		0.00	2.36	10	0.04		0.00	-0.01	0.00		0.12	0.09
methylchrysene	0.68	-0.01	0.00	7	0.97	225.82	-15.03	0.00		0.54	-0.01	0.00	7	0.78	24.94	-4.99	0.00		0.00	2.42	10	0.04		-0.01	-0.01	0.00		0.21	0.11
dimethylchrysene	1.09	-0.01	0.00	7	0.97	259.83	-16.12	0.00		0.95	-0.01	0.00	7	0.79	26.64	-5.16	0.00		0.00	2.29	10	0.05		-0.01	-0.01	0.00		0.24	0.12
trimethylchrysene	0.80	-0.02	0.00	7	0.98	450.25	-21.22	0.00		0.66	-0.01	0.00	7	0.78	24.21	-4.92	0.00		0.00	2.20	10	0.05		-0.01	-0.02	na		0.29	
tetramethylchrysene	0.51	-0.02	0.00	7	0.97	265.29	-16.29	0.00		0.30	-0.01	0.00	7	0.76	22.32	-4.72	0.00		0.00	3.04	10	0.01		-0.01	-0.02	-0.01		0.24	0.17
benzo[b]fluoranthene	-0.58	-0.01	0.00	7	0.96	177.77	-13.33	0.00		-0.67	0.00	0.00	7	0.79	26.77	-5.17	0.00		0.00	4.03	10	0.00		0.00	-0.01	0.00		0.08	0.08
benzo[e]pyrene	-0.96	-0.01	0.00	7	0.92	80.63	-8.98	0.00		-1.14	0.00	0.00	7	0.42	5.11	-2.26	0.06							na	-0.01	-0.01			0.16
benzo[ghi]perylene	-1.47	-0.01	0.00	7	0.96	152.03	-12.33	0.00		-1.57	0.00	0.00	7	0.70	16.20	-4.03	0.01		0.00	3.68	10	0.00		0.00	-0.01	0.00		0.07	0.08

Table S3 Linear regression statistics for the live and killed control incubations from Stn11. For each treatment, a t-test was conducted to determine if the slope was significantly different from zero. A second t-test compared the slope from the killed control incubations to those of the live incubations. The estimated rate constant is calculated for abiotic degradation or biodegradation.

Stn11	Linear Regressions																		t-test for Significant difference between slopes					Rate constant (h-1)				Rate constant (d-1)	
	Live									Killed Control																			
Analyte	Intercept	Slope	SlopeSE	df	R2	Fstat	t-value	p-value		Intercept	Slope	SlopeSE	df	R2	Fstat	t-value	p-value		Error	t statistic	df	p-value		Abiotic	Live	Biodegradation		Abiotic	Biodegrada
C13	2.31	0.00	0.00	7	0.57	9.19	-3.03	0.02		2.28	0.00	0.00	7	0.73	18.70	-4.32	0.00		0.00	-0.36	10	0.72		0.00	0.00	na		0.08	
C14	2.36	0.00	0.00	7	0.51	7.29	-2.70	0.03		2.29	0.00	0.00	7	0.68	14.60	-3.82	0.01		0.00	0.20	10	0.84		0.00	0.00	na		0.08	
C15	2.35	0.00	0.00	7	0.50	6.91	-2.63	0.03		2.29	0.00	0.00	7	0.68	15.18	-3.90	0.01		0.00	0.12	10	0.91		0.00	0.00	na		0.08	
C16	2.42	0.00	0.00	7	0.31	3.12	-1.77	0.12		2.37	0.00	0.00	7	0.65	13.19	-3.63	0.01							0.00	na	0.00		0.07	
C17	2.48	0.00	0.00	7	0.14	1.12	-1.06	0.33		2.47	0.00	0.00	7	0.69	15.32	-3.91	0.01							0.00	na	0.00		0.08	
C18	2.16	0.00	0.00	7	0.28	2.78	-1.67	0.14		2.12	0.00	0.00	7	0.64	12.46	-3.53	0.01							0.00	na	0.00		0.07	
C19	2.05	0.00	0.00	7	0.36	3.92	-1.98	0.09		1.98	0.00	0.00	7	0.82	31.46	-5.61	0.00							0.00	na	0.00		0.08	
C20	1.90	0.00	0.00	7	0.33	3.50	-1.87	0.10		1.86	0.00	0.00	7	0.81	29.16	-5.40	0.00							0.00	na	0.00		0.08	
C21	1.82	0.00	0.00	7	0.34	3.61	-1.90	0.10		1.78	0.00	0.00	7	0.74	19.73	-4.44	0.00							0.00	na	0.00		0.07	
C22	1.77	0.00	0.00	7	0.33	3.39	-1.84	0.11		1.73	0.00	0.00	7	0.78	24.32	-4.93	0.00							0.00	na	0.00		0.08	
C23	1.67	0.00	0.00	7	0.33	3.37	-1.84	0.11		1.61	0.00	0.00	7	0.71	16.76	-4.09	0.00							0.00	na	0.00		0.07	
C24	1.68	0.00	0.00	7	0.23	2.05	-1.43	0.20		1.67	0.00	0.00	7	0.80	28.81	-5.37	0.00							0.00	na	0.00		0.08	
C25	1.56	0.00	0.00	7	0.37	4.12	-2.03	0.08		1.51	0.00	0.00	7	0.78	24.74	-4.97	0.00							0.00	na	0.00		0.08	
C26	1.47	0.00	0.00	7	0.22	1.92	-1.39	0.21		1.44	0.00	0.00	7	0.68	14.62	-3.82	0.01							0.00	na	0.00		0.08	
C27	1.35	0.00	0.00	7	0.30	2.98	-1.73	0.13		1.31	0.00	0.00	7	0.73	18.66	-4.32	0.00							0.00	na	0.00		0.09	
C28	1.35	0.00	0.00	7	0.35	3.82	-1.95	0.09		1.29	0.00	0.00	7	0.77	22.81	-4.78	0.00							0.00	na	0.00		0.10	
C29	1.21	0.00	0.00	7	0.39	4.39	-2.10	0.07		1.12	-0.01	0.00	7	0.68	15.18	-3.90	0.01							-0.01	na	0.00		0.12	
C30	0.95	0.00	0.00	7	0.35	3.81	-1.95	0.09		0.96	0.00	0.00	6	0.75	17.88	-4.23	0.01							0.00	na	0.00		0.10	
C31	0.65	0.00	0.00	7	0.24	2.22	-1.49	0.18		0.61	0.00	0.00	7	0.71	17.41	-4.17	0.00							0.00	na	0.00		0.09	
C32	0.31	0.00	0.00	7	0.08	0.65	-0.80	0.45		0.29	0.00	0.00	7	0.79	25.95	-5.09	0.00							0.00	na	0.00		0.06	
C33	0.11	0.00	0.00	7	0.15	1.20	-1.10	0.31		0.11	0.00	0.00	7	0.74	20.29	-4.50	0.00							0.00	na	0.00		0.12	
C34	-0.22	0.00	0.00	7	0.00	0.01	-0.08	0.94		-0.19	-0.01	0.00	7	0.62	11.28	-3.36	0.01							-0.01	na	0.00		0.14	
C35	-0.60	0.00	0.00	6	0.01	0.07	0.27	0.80		-0.52	0.00	0.00	7	0.63	10.01	-3.16	0.02							0.00	na	0.00		0.10	
naphthalene	2.00	0.00	0.00	7	0.48	6.52	-2.55	0.04		1.96	0.00	0.00	7	0.64	12.33	-3.51	0.01		0.00	0.29	10	0.78		0.00	0.00	na		0.07	
methylnaphthalene	2.84	0.00	0.00	7	0.46	5.98	-2.44	0.04		2.81	0.00	0.00	7	0.67	14.16	-3.76	0.01		0.00	-0.11	10	0.91		0.00	0.00	na		0.08	
dimethylnaphthalene	3.45	0.00	0.00	7	0.40	4.63	-2.15	0.07		3.42	0.00	0.00	7	0.70	16.70	-4.09	0.00							0.00	na	na		0.09	
trimethylnaphthalene	3.33	0.00	0.00	7	0.52	7.58	-2.75	0.03		3.30	-0.01	0.00	7	0.82	31.07	-5.57	0.00		0.00	-0.36	10	0.72		-0.01	0.00	na		0.13	
tetramethylnaphthalene	2.89	-0.01	0.00	7	0.62	11.34	-3.37	0.01		2.87	-0.01	0.00	7	0.87	46.46	-6.82	0.00		0.00	-0.33	10	0.75		-0.01	-0.01	na		0.16	
acenaphthene	-1.01	0.00	0.00	7	0.48	6.57	-2.56	0.04		-1.03	-0.01	0.00	7	0.81	30.67	-5.54	0.00		0.00	-0.38	10	0.71		-0.01	0.00	na		0.13	
fluorene	1.02	0.00	0.00	7	0.42	4.97	-2.23	0.06		1.00	0.00	0.00	7	0.76	22.20	-4.71	0.00							0.00	na	na		0.10	
methylfluorene	1.65	0.00	0.00	7	0.51	7.29	-2.70	0.03		1.60	0.00	0.00	7	0.75	20.97	-4.58	0.00		0.00	-0.10	10	0.92		0.00	0.00	na		0.11	
dimethylfluorene	1.82	-0.01	0.00	7	0.64	12.23	-3.50	0.01		1.82	-0.01	0.00	7	0.89	56.66	-7.53	0.00		0.00	-0.41	10	0.69		-0.01	-0.01	na		0.16	
trimethylfluorene	1.76	0.00	0.00	7	0.44	5.57	-2.36	0.05		1.78	-0.01	0.00	7	0.81	29.80	-5.46	0.00							-0.01	na	na		0.12	
dibenzothiophene	0.62	0.00	0.00	7	0.24	2.24	-1.50	0.18		0.58	0.00	0.00	7	0.64	12.71	-3.56	0.01							0.00	na	na		0.08	
methyldibenzothiophene	2.43	-0.01	0.00	7	0.58	9.76	-3.12	0.02		2.39	-0.01	0.00	7	0.84	35.58	-5.97	0.00		0.00	-0.24	10	0.82		-0.01	-0.01	na		0.14	
dimethyldibenzothiophene	2.05	0.00	0.00	7	0.16	1.38	-1.18	0.28		2.02	0.00	0.00	7	0.67	14.33	-3.79	0.01							0.00	na	na		0.06	
trimethyldibenzothiophene	1.93	0.00	0.00	7	0.15	1.28	-1.13	0.30		1.92	0.00	0.00	7	0.64	12.44	-3.53	0.01							0.00	na	na		0.06	
tetramethyldibenzothiophene	1.42	0.00	0.00	7	0.04	0.32	-0.57	0.59		1.43	0.00	0.00	7	0.62	11.32	-3.36	0.01							0.00	na	na		0.05	
phenanthrene	1.70	0.00	0.00	7	0.16	1.30	-1.14	0.29		1.66	0.00	0.00	7	0.56	8.92	-2.99	0.02							0.00	na	na		0.05	
methylphenanthrene	2.43	0.00	0.00	7	0.20	1.71	-1.31	0.23		2.40	0.00	0.00	7	0.66	13.50	-3.67	0.01							0.00	na	na		0.06	
dimethylphenanthrene	2.63	0.00	0.00	7	0.25	2.30	-1.52	0.17		2.60	0.00	0.00	7	0.73	19.04	-4.36	0.00							0.00	na	na		0.07	
trimethylphenanthrene	2.49	0.00	0.00	7	0.34	3.59	-1.89	0.10		2.46	0.00	0.00	7	0.85	41.13	-6.41	0.00							0.00	na	na		0.10	
tetramethylphenanthrene	2.00	0.00	0.00	7	0.21	1.87	-1.37	0.21		2.04	0.00	0.00	7	0.83	35.05	-5.92	0.00							0.00	na	na		0.12	
fluoranthene	-0.81	0.00	0.00	7	0.34	3.59	-1.89	0.10		-0.94	0.00	0.00	7	0.76	21.78	-4.67	0.00							0.00	na	na		0.10	
pyrene	-0.44	0.00	0.00	7	0.13	1.06	-1.03	0.34		-0.44	0.00	0.00	7	0.86	42.08	-6.49	0.00							0.00	na	na		0.08	
methylpyrene	0.94	0.00	0.00	7	0.33	3.42	-1.85	0.11		0.93	0.00	0.00	7	0.91	67.59	-8.22	0.00							0.00	na	na		0.09	
dimethylpyrene	0.99	0.00	0.00	7	0.51	7.28	-2.70	0.03		0.99	-0.01	0.00	7	0.85	40.41	-6.36	0.00		0.00	-0.38	10	0.71		-0.01	0.00	na		0.12	
trimethylpyrene	0.86	-0.01	0.00	7	0.52	7.64	-2.76	0.03		0.88	-0.01	0.00	7	0.90	61.04	-7.81	0.00		0.00	-0.52	10	0.62		-0.01	-0.01	na		0.15	
tetramethylpyrene	1.25	0.00	0.00	7	0.45	5.78	-2.40	0.05		1.18	0.00	0.00	7	0.30	3.05	-1.75	0.12							na	0.00	0.00			0.11
naphthobenzothiophene	-0.96	0.00	0.00	7	0.31	3.21	-1.79	0.12		-1.02	0.00	0.00	7	0.80	27.19	-5.21	0.00							0.00	na	na		0.09	
methylnaphthobenzothiophene	0.52	0.00	0.00	7	0.15	1.22	-1.11	0.31		0.49	0.00	0.00	7	0.66	13.64	-3.69	0.01							0.00	na	na		0.07	
dimethylNBenzothiophene	0.89	0.00	0.00	7	0.20	1.72	-1.31	0.23		0.85	0.00	0.00	7	0.67	14.42	-3.80	0.01							0.00	na	na		0.07	
trimethylNbenzothiophene	0.67	0.00	0.00	7	0.28	2.70	-1.64	0.14		0.65	0.00	0.00	7	0.75	20.99	-4.58	0.00							0.00	na	na		0.09	
tetramethylNbenzothiophene	0.12	0.00	0.00	7	0.33	3.47	-1.86	0.10		0.11	0.00	0.00	7	0.75	21.12	-4.60	0.00							0.00	na	na		0.09	
benz[a]anthracene	-2.42	-0.01	0.00	7	0.72	18.30	-4.28	0.00		-2.42	-0.01	0.00	7	0.80	28.52	-5.34	0.00		0.00	-0.22	10	0.83		-0.01	-0.01	na		0.22	
chrysene	-0.55	0.00	0.00	7	0.26	2.45	-1.57	0.16		-0.59	0.00	0.00	7	0.79	25.93	-5.09	0.00							0.00	na	na		0.08	
methylchrysene	0.21	-0.01	0.00	7	0.57	9.19	-3.03	0.02		0.22	-0.01	0.00	7	0.87	46.25	-6.80	0.00		0.00	-0.68	10	0.51		-0.01	-0.01	na		0.17	
dimethylchrysene	0.58	-0.01	0.00	7	0.64	12.62	-3.55	0.01		0.60	-0.01	0.00	7	0.89	58.39	-7.64	0.00		0.00	-0.56	10	0.59		-0.01	-0.01	na		0.23	
trimethylchrysene	0.30	-0.01	0.00	7	0.83	35.05	-5.92	0.00		0.28	-0.01	0.00	7	0.86	44.35	-6.66	0.00		0.00	-0.08	10	0.94		-0.01	-0.01	na		0.23	
tetramethylchrysene	0.04	-0.01	0.00	7	0.68	15.04	-3.88	0.01		0.04	-0.01	0.00	7	0.91	67.38	-8.21	0.00		0.00	-0.50	10	0.63		-0.01	-0.01	na		0.22	
benzo[b]fluoranthene	-1.48	0.00	0.00	7	0.20	1.74	-1.32	0.23		-1.48	0.00	0.00	7	0.77	23.67	-4.87	0.00							0.00	na	na		0.09	
benzo[e]pyrene	-1.37	0.00	0.00	7	0.17	1.40	-1.18	0.28		-1.37	0.00	0.00	7	0.66	13.85	-3.72	0.01							0.00	na	na		0.07	
benzo[ghi]perylene	-2.26	-0.01	0.00	6	0.88	44.83	-6.70	0.00		-2.46	0.00	0.00	7	0.67	14.14	-3.76	0.01		0.00	1.62	9	0.14		0.00	-0.01	na		0.08	

Table S4 Linear regression statistics for the live and killed control incubations from Stn20. For each treatment, a t-test was conducted to determine if the slope was significantly different from zero. A second t-test compared the slope from the killed control incubations to those of the live incubations. The estimated rate constant is calculated for abiotic degradation or biodegradation.

Stn20	Linear Regressions							
	Live							
Analyte	Intercept	Slope	SlopeSE	df	R2	Fstat	t-value	p-value
C13	2.33	0.00	0.00	7	0.76	21.63	-4.65	0.00
C14	2.36	0.00	0.00	7	0.71	16.84	-4.10	0.00
C15	2.38	0.00	0.00	7	0.71	17.14	-4.14	0.00
C16	2.40	0.00	0.00	7	0.59	10.15	-3.19	0.02
C17	2.45	0.00	0.00	7	0.72	18.35	-4.28	0.00
C18	2.15	0.00	0.00	7	0.44	5.44	-2.33	0.05
C19	2.01	0.00	0.00	7	0.55	8.65	-2.94	0.02
C20	1.91	0.00	0.00	7	0.51	7.40	-2.72	0.03
C21	1.82	0.00	0.00	7	0.52	7.60	-2.76	0.03
C22	1.77	0.00	0.00	7	0.57	9.28	-3.05	0.02
C23	1.69	0.00	0.00	7	0.47	6.18	-2.49	0.04
C24	1.69	0.00	0.00	7	0.59	10.14	-3.18	0.02
C25	1.58	0.00	0.00	7	0.54	8.17	-2.86	0.02
C26	1.52	0.00	0.00	7	0.54	8.26	-2.87	0.02
C27	1.36	0.00	0.00	7	0.52	7.64	-2.76	0.03
C28	1.33	0.00	0.00	7	0.55	8.52	-2.92	0.02
C29	1.17	0.00	0.00	7	0.55	8.45	-2.91	0.02
C30	0.94	0.00	0.00	7	0.33	3.38	-1.84	0.11
C31	0.60	0.00	0.00	7	0.47	6.26	-2.50	0.04
C32	0.33	0.00	0.00	7	0.28	2.67	-1.63	0.15
C33	0.11	0.00	0.00	7	0.56	8.98	-3.00	0.02
C34	-0.25	0.00	0.00	7	0.00	0.01	0.09	0.93
C35	-0.69	0.00	0.00	7	0.00	0.00	0.01	0.99
naphthalene	2.00	0.00	0.00	7	0.61	10.86	-3.30	0.01
methylnaphthalene	2.79	0.00	0.00	7	0.64	12.50	-3.54	0.01
dimethylnaphthalene	3.36	0.00	0.00	7	0.71	17.08	-4.13	0.00
trimethylnaphthalene	3.19	-0.01	0.00	7	0.79	26.31	-5.13	0.00
tetramethylnaphthalene	2.80	-0.01	0.00	7	0.83	33.71	-5.81	0.00
acenaphthene	-1.10	-0.01	0.00	7	0.82	31.36	-5.60	0.00
fluorene	0.99	0.00	0.00	7	0.77	22.98	-4.79	0.00
methylfluorene	1.61	-0.01	0.00	7	0.79	26.49	-5.15	0.00
dimethylfluorene	1.80	-0.01	0.00	7	0.82	31.94	-5.65	0.00
trimethylfluorene	1.73	-0.01	0.00	7	0.71	16.86	-4.11	0.00
dibenzothiophene	0.63	0.00	0.00	7	0.59	10.20	-3.19	0.02
methyldibenzothiophene	2.23	-0.01	0.00	7	0.56	8.82	-2.97	0.02
dimethyldibenzothiophene	2.00	0.00	0.00	7	0.47	6.15	-2.48	0.04
trimethyldibenzothiophene	1.87	0.00	0.00	7	0.45	5.76	-2.40	0.05
tetramethyldibenzothiophene	1.35	0.00	0.00	7	0.12	0.95	-0.98	0.36
phenanthrene	1.68	0.00	0.00	7	0.45	5.75	-2.40	0.05
methylphenanthrene	2.40	0.00	0.00	7	0.48	6.56	-2.56	0.04
dimethylphenanthrene	2.58	0.00	0.00	7	0.52	7.61	-2.76	0.03
trimethylphenanthrene	2.41	0.00	0.00	7	0.63	12.07	-3.47	0.01
tetramethylphenanthrene	1.98	0.00	0.00	7	0.78	24.17	-4.92	0.00
fluoranthene	-0.84	-0.01	0.00	7	0.40	4.65	-2.16	0.07
pyrene	-0.44	0.00	0.00	7	0.33	3.51	-1.87	0.10
methylpyrene	0.88	0.00	0.00	7	0.62	11.58	-3.40	0.01
dimethylpyrene	0.93	0.00	0.00	7	0.58	9.74	-3.12	0.02
trimethylpyrene	0.78	0.00	0.00	7	0.57	9.12	-3.02	0.02
tetramethylpyrene	1.23	-0.01	0.00	7	0.81	29.68	-5.45	0.00
naphthobenzothiophene	-0.99	0.00	0.00	7	0.64	12.66	-3.56	0.01
methylnaphthobenzothiophene	0.52	0.00	0.00	7	0.53	7.82	-2.80	0.03
dimethylNBenzothiophene	0.87	0.00	0.00	7	0.53	8.00	-2.83	0.03
trimethylNbenzothiophene	0.63	0.00	0.00	7	0.43	5.27	-2.29	0.06
tetramethylNbenzothiophene	0.10	0.00	0.00	7	0.65	13.00	-3.61	0.01
benz[a]anthracene	-2.34	-0.01	0.00	7	0.89	59.25	-7.70	0.00
chrysene	-0.54	0.00	0.00	7	0.64	12.26	-3.50	0.01
methylchrysene	0.25	-0.01	0.00	7	0.78	25.49	-5.05	0.00
dimethylchrysene	0.67	-0.01	0.00	7	0.90	65.22	-8.08	0.00
trimethylchrysene	0.31	-0.01	0.00	7	0.79	26.94	-5.19	0.00
tetramethylchrysene	0.12	-0.01	0.00	7	0.81	30.62	-5.53	0.00
benzo[b]fluoranthene	-1.46	0.00	0.00	7	0.62	11.49	-3.39	0.01
benzo[e]pyrene	-1.42	0.00	0.00	7	0.42	5.07	-2.25	0.06
benzo[ghi]perylene	-2.42	0.00	0.00	7	0.49	6.78	-2.60	0.04

	Killed Control							
Analyte	Intercept	Slope	SlopeSE	df	R2	Fstat	t-value	p-value
C13	2.28	0.00	0.00	7	0.81	30.08	-5.48	0.00
C14	2.31	0.00	0.00	7	0.71	16.78	-4.10	0.00
C15	2.34	0.00	0.00	7	0.71	16.96	-4.12	0.00
C16	2.36	0.00	0.00	7	0.62	11.23	-3.35	0.01
C17	2.38	0.00	0.00	7	0.44	5.58	-2.36	0.05
C18	2.11	0.00	0.00	7	0.50	6.91	-2.63	0.03
C19	1.99	0.00	0.00	7	0.64	12.61	-3.55	0.01
C20	1.86	0.00	0.00	7	0.62	11.42	-3.38	0.01
C21	1.78	0.00	0.00	7	0.55	8.62	-2.94	0.02
C22	1.72	0.00	0.00	7	0.53	7.82	-2.80	0.03
C23	1.66	0.00	0.00	7	0.46	6.04	-2.46	0.04
C24	1.66	0.00	0.00	7	0.68	15.10	-3.89	0.01
C25	1.53	0.00	0.00	7	0.58	9.64	-3.11	0.02
C26	1.49	0.00	0.00	7	0.66	13.46	-3.67	0.01
C27	1.31	0.00	0.00	7	0.55	8.58	-2.93	0.02
C28	1.28	0.00	0.00	7	0.64	12.66	-3.56	0.01
C29	1.12	0.00	0.00	7	0.59	10.26	-3.20	0.02
C30	0.88	0.00	0.00	7	0.29	2.90	-1.70	0.13
C31	0.56	0.00	0.00	7	0.60	10.50	-3.24	0.01
C32	0.29	0.00	0.00	7	0.27	2.59	-1.61	0.15
C33	0.01	0.00	0.00	7	0.29	2.80	-1.67	0.14
C34	-0.27	0.00	0.00	7	0.23	2.06	-1.44	0.19
C35	-0.77	0.00	0.00	7	0.24	1.89	1.37	0.21
naphthalene	1.93	0.00	0.00	7	0.28	2.67	-1.63	0.15
methylnaphthalene	2.74	0.00	0.00	7	0.52	7.71	-2.78	0.03
dimethylnaphthalene	3.31	0.00	0.00	7	0.68	15.01	-3.87	0.01
trimethylnaphthalene	3.16	0.00	0.00	7	0.76	22.55	-4.75	0.00
tetramethylnaphthalene	2.74	-0.01	0.00	7	0.80	27.15	-5.21	0.00
acenaphthene	-1.12	0.00	0.00	7	0.84	36.30	-6.03	0.00
fluorene	0.96	0.00	0.00	7	0.79	27.06	-5.20	0.00
methylfluorene	1.56	-0.01	0.00	7	0.79	26.14	-5.11	0.00
dimethylfluorene	1.74	-0.01	0.00	7	0.87	45.49	-6.74	0.00
trimethylfluorene	1.74	-0.01	0.00	7	0.69	15.72	-3.96	0.01
dibenzothiophene	0.58	0.00	0.00	7	0.54	8.34	-2.89	0.02
methyldibenzothiophene	2.25	-0.01	0.00	7	0.81	29.02	-5.39	0.00
dimethyldibenzothiophene	1.96	0.00	0.00	7	0.40	4.61	-2.15	0.07
trimethyldibenzothiophene	1.83	0.00	0.00	7	0.39	4.54	-2.13	0.07
tetramethyldibenzothiophene	1.29	0.00	0.00	7	0.04	0.30	-0.55	0.60
phenanthrene	1.63	0.00	0.00	7	0.40	4.69	-2.16	0.07
methylphenanthrene	2.35	0.00	0.00	7	0.44	5.52	-2.35	0.05
dimethylphenanthrene	2.53	0.00	0.00	7	0.50	6.94	-2.63	0.03
trimethylphenanthrene	2.34	0.00	0.00	7	0.56	8.81	-2.97	0.02
tetramethylphenanthrene	1.92	0.00	0.00	7	0.47	6.12	-2.47	0.04
fluoranthene	-0.98	0.00	0.00	7	0.62	11.62	-3.41	0.01
pyrene	-0.48	0.00	0.00	7	0.50	6.92	-2.63	0.03
methylpyrene	0.87	0.00	0.00	7	0.67	14.11	-3.76	0.01
dimethylpyrene	0.90	0.00	0.00	7	0.67	14.23	-3.77	0.01
trimethylpyrene	0.72	0.00	0.00	7	0.34	3.64	-1.91	0.10
tetramethylpyrene	1.20	-0.01	0.00	7	0.72	17.61	-4.20	0.00
naphthobenzothiophene	-1.02	0.00	0.00	7	0.65	12.75	-3.57	0.01
methylnaphthobenzothiophene	0.48	0.00	0.00	7	0.42	5.08	-2.25	0.06
dimethylNBenzothiophene	0.82	0.00	0.00	7	0.40	4.59	-2.14	0.07
trimethylNbenzothiophene	0.63	0.00	0.00	7	0.69	15.49	-3.94	0.01
tetramethylNbenzothiophene	0.05	0.00	0.00	7	0.64	12.66	-3.56	0.01
benz[a]anthracene	-2.34	-0.01	0.00	7	0.92	80.49	-8.97	0.00
chrysene	-0.59	0.00	0.00	7	0.57	9.38	-3.06	0.02
methylchrysene	0.17	-0.01	0.00	7	0.78	25.52	-5.05	0.00
dimethylchrysene	0.59	-0.01	0.00	7	0.84	35.49	-5.96	0.00
trimethylchrysene	0.27	-0.01	0.00	7	0.79	26.95	-5.19	0.00
tetramethylchrysene	0.07	-0.01	0.00	7	0.73	18.86	-4.34	0.00
benzo[b]fluoranthene	-1.49	0.00	0.00	7	0.67	14.14	-3.76	0.01
benzo[e]pyrene	-1.42	0.00	0.00	7	0.60	10.39	-3.22	0.01
benzo[ghi]perylene	-2.44	0.00	0.00	7	0.64	12.27	-3.50	0.01

	t-test for Significant difference between slopes				Rate constant (h-1)			Rate constant (d-1)	
Analyte	Error	t statistic	df	p-value	Abiotic	Live	Biodegradation	Abiotic	Biodegrada
C13	0.00	1.16	10	0.27	0.00	0.00	na	0.06	
C14	0.00	0.78	10	0.45	0.00	0.00	na	0.06	
C15	0.00	0.49	10	0.64	0.00	0.00	na	0.07	
C16	0.00	0.56	10	0.59	0.00	0.00	na	0.07	
C17					na	0.00	0.00		0.11
C18					0.00	na	na	0.04	
C19	0.00	0.64	10	0.54	0.00	0.00	na	0.06	
C20	0.00	0.48	10	0.64	0.00	0.00	na	0.06	
C21	0.00	0.55	10	0.60	0.00	0.00	na	0.06	
C22	0.00	0.59	10	0.57	0.00	0.00	na	0.06	
C23	0.00	0.36	10	0.73	0.00	0.00	na	0.06	
C24	0.00	0.16	10	0.88	0.00	0.00	na	0.07	
C25	0.00	0.45	10	0.66	0.00	0.00	na	0.07	
C26	0.00	0.54	10	0.60	0.00	0.00	na	0.06	
C27	0.00	0.46	10	0.66	0.00	0.00	na	0.05	
C28	0.00	0.25	10	0.81	0.00	0.00	na	0.07	
C29	0.00	0.00	10	1.00	0.00	0.00	na	0.08	
C30					na	na	na		
C31	0.00	0.54	10	0.60	0.00	0.00	na	0.05	
C32					na	na	na		
C33					na	0.00	0.00		0.08
C34					na	na	na		
C35					na	na	na		
naphthalene					na	0.00	0.00		0.10
methylnaphthalene	0.00	1.07	10	0.31	0.00	0.00	na	0.06	
dimethylnaphthalene	0.00	0.79	10	0.45	0.00	0.00	na	0.08	
trimethylnaphthalene	0.00	0.33	10	0.75	0.00	-0.01	na	0.12	
tetramethylnaphthalene	0.00	0.41	10	0.69	-0.01	-0.01	na	0.16	
acenaphthene	0.00	0.34	10	0.74	0.00	-0.01	na	0.12	
fluorene	0.00	0.23	10	0.82	0.00	0.00	na	0.11	
methylfluorene	0.00	0.64	10	0.54	-0.01	-0.01	na	0.12	
dimethylfluorene	0.00	0.61	10	0.56	-0.01	-0.01	na	0.15	
trimethylfluorene	0.00	0.60	10	0.56	-0.01	-0.01	na	0.12	
dibenzothiophene	0.00	0.70	10	0.50	0.00	0.00	na	0.07	
methyldibenzothiophene	0.00	-0.28	10	0.78	-0.01	-0.01	na	0.14	
dimethyldibenzothiophene					na	0.00	0.00		0.07
trimethyldibenzothiophene					na	0.00	0.00		0.07
tetramethyldibenzothiophene					na	na	na		
phenanthrene					na	0.00	0.00		0.06
methylphenanthrene					na	0.00	0.00		0.07
dimethylphenanthrene	0.00	0.69	10	0.51	0.00	0.00	na	0.05	
trimethylphenanthrene	0.00	1.00	10	0.34	0.00	0.00	na	0.06	
tetramethylphenanthrene	0.00	0.86	10	0.41	0.00	0.00	na	0.07	
fluoranthene					0.00	na	na	0.07	
pyrene					0.00	na	na	0.07	
methylpyrene	0.00	0.23	10	0.82	0.00	0.00	na	0.07	
dimethylpyrene	0.00	0.31	10	0.77	0.00	0.00	na	0.09	
trimethylpyrene					na	0.00	0.00		0.11
tetramethylpyrene	0.00	0.21	10	0.84	-0.01	-0.01	na	0.14	
naphthobenzothiophene	0.00	0.23	10	0.83	0.00	0.00	na	0.08	
methylnaphthobenzothiophene					na	0.00	0.00		0.08
dimethylNBenzothiophene					na	0.00	0.00		0.08
trimethylNbenzothiophene	0.00	-0.33	10	0.75	0.00	na	na	0.09	
tetramethylNbenzothiophene	0.00	0.28	10	0.79	0.00	0.00	na	0.09	
benz[a]anthracene	0.00	-0.02	10	0.98	-0.01	-0.01	na	0.26	
chrysene	0.00	0.74	10	0.48	0.00	0.00	na	0.07	
methylchrysene	0.00	0.76	10	0.47	-0.01	-0.01	na	0.16	
dimethylchrysene	0.00	0.98	10	0.35	-0.01	-0.01	na	0.21	
trimethylchrysene	0.00	0.07	10	0.95	-0.01	-0.01	na	0.24	
tetramethylchrysene	0.00	0.46	10	0.65	-0.01	-0.01	na	0.23	
benzo[b]fluoranthene	0.00	0.24	10	0.81	0.00	0.00	na	0.08	
benzo[e]pyrene					0.00	na	na	0.06	
benzo[ghi]perylene	0.00	0.05	10	0.96	0.00	0.00	na	0.06	

Table S5 Linear regression statistics for the live and killed control incubations from CGR01. For each treatment, a t-test was conducted to determine if the slope was significantly different from zero. A second t-test compared the slope from the killed control incubations to those of the live incubations. The estimated rate constant is calculated for abiotic degradation or biodegradation.

CGR01	Linear Regressions															
	Live								Killed Control							
Analyte	Intercept	Slope	SlopeSE	df	R2	Fstat	t-value	p-value	Intercept	Slope	SlopeSE	df	R2	Fstat	t-value	p-value
C13	2.07	0.00	0.00	7	0.03	0.25	-0.50	0.63	2.25	0.00	0.00	7	0.35	3.83	-1.96	0.09
C14	2.07	0.00	0.00	7	0.03	0.25	-0.50	0.63	2.29	0.00	0.00	7	0.24	2.21	-1.49	0.18
C15	2.07	0.00	0.00	7	0.04	0.30	-0.55	0.60	2.32	0.00	0.00	7	0.23	2.12	-1.46	0.19
C16	2.11	0.00	0.00	7	0.08	0.65	-0.81	0.45	2.36	0.00	0.00	7	0.40	4.73	-2.17	0.07
C17	2.23	0.00	0.00	7	0.21	1.81	-1.35	0.22	2.51	0.00	0.00	7	0.31	3.11	-1.76	0.12
C18	1.92	0.00	0.00	7	0.09	0.65	-0.81	0.45	2.14	0.00	0.00	7	0.37	4.08	-2.02	0.08
C19	1.92	0.00	0.00	7	0.12	0.97	-0.99	0.36	2.16	0.00	0.00	7	0.38	4.24	-2.06	0.08
C20	1.55	0.00	0.00	7	0.15	1.26	-1.12	0.30	1.78	0.00	0.00	7	0.37	4.05	-2.01	0.08
C21	1.51	0.00	0.00	7	0.18	1.52	-1.23	0.26	1.73	0.00	0.00	7	0.36	3.91	-1.98	0.09
C22	1.52	0.00	0.00	7	0.30	2.94	-1.71	0.13	1.71	0.00	0.00	7	0.38	4.30	-2.07	0.08
C23	1.52	0.00	0.00	7	0.34	3.60	-1.90	0.10	1.72	0.00	0.00	7	0.35	3.83	-1.96	0.09
C24	1.55	0.00	0.00	7	0.30	3.05	-1.75	0.12	1.78	0.00	0.00	7	0.38	4.26	-2.06	0.08
C25	1.74	0.00	0.00	7	0.26	2.47	-1.57	0.16	1.96	0.00	0.00	7	0.38	4.26	-2.07	0.08
C26	2.47	0.00	0.00	7	0.18	1.56	-1.25	0.25	2.73	0.00	0.00	7	0.37	4.06	-2.02	0.08
C27	2.49	0.00	0.00	7	0.23	2.14	-1.46	0.19	2.75	0.00	0.00	7	0.37	4.10	-2.02	0.08
C28	2.16	-0.01	0.00	7	0.30	3.05	-1.75	0.12	2.38	0.00	0.00	7	0.33	3.47	-1.86	0.10
C29	2.17	-0.01	0.00	7	0.42	5.13	-2.27	0.06	2.45	-0.01	0.00	7	0.43	5.22	-2.29	0.06
C30	0.65	0.00	0.00	7	0.12	0.91	-0.96	0.37	0.91	0.00	0.00	7	0.38	4.30	-2.07	0.08
C31	0.63	0.00	0.00	7	0.24	2.26	-1.50	0.18	0.85	0.00	0.00	7	0.35	3.71	-1.93	0.10
C32	0.19	0.00	0.00	7	0.51	7.29	-2.70	0.03	0.38	0.00	0.00	7	0.36	3.88	-1.97	0.09
C33	0.13	-0.01	0.00	7	0.28	2.72	-1.65	0.14	0.35	0.00	0.00	7	0.27	2.55	-1.60	0.15
C34	0.00	-0.01	0.00	7	0.71	17.37	-4.17	0.00	0.15	0.00	0.00	7	0.41	4.85	-2.20	0.06
C35	-0.20	-0.01	0.00	6	0.71	14.44	-3.80	0.01	-0.09	0.00	0.00	7	0.56	7.54	-2.75	0.03
naphthalene	1.74	0.00	0.00	7	0.10	0.76	-0.87	0.41	1.98	-0.01	0.00	7	0.53	7.81	-2.79	0.03
methylnaphthalene	2.52	0.00	0.00	7	0.11	0.84	-0.92	0.39	2.83	-0.01	0.01	7	0.31	3.08	-1.75	0.12
dimethylnaphthalene	3.12	0.00	0.00	7	0.17	1.39	-1.18	0.28	3.35	-0.01	0.00	7	0.49	6.69	-2.59	0.04
trimethylnaphthalene	3.02	-0.01	0.00	7	0.33	3.38	-1.84	0.11	3.27	-0.01	0.00	7	0.61	10.83	-3.29	0.01
tetramethylnaphthalene	2.59	-0.01	0.00	7	0.29	2.85	-1.69	0.14	2.87	-0.01	0.00	7	0.68	14.95	-3.87	0.01
acenaphthene	-1.10	-0.01	0.00	7	0.67	13.95	-3.73	0.01	-0.93	-0.01	0.00	7	0.71	17.46	-4.18	0.00
fluorene	0.79	-0.01	0.00	7	0.28	2.79	-1.67	0.14	1.07	-0.01	0.00	7	0.65	12.91	-3.59	0.01
methylfluorene	1.45	-0.01	0.00	7	0.36	3.87	-1.97	0.09	1.70	-0.01	0.00	7	0.70	16.65	-4.08	0.00
dimethylfluorene	1.57	-0.01	0.00	7	0.38	4.24	-2.06	0.08	1.84	-0.01	0.00	7	0.74	20.16	-4.49	0.00
trimethylfluorene	1.52	0.00	0.00	7	0.06	0.43	-0.65	0.53	1.85	-0.01	0.00	7	0.69	15.29	-3.91	0.01
dibenzothiophene	0.47	0.00	0.00	7	0.13	1.07	-1.04	0.33	0.76	-0.01	0.00	7	0.43	5.23	-2.29	0.06
methyldibenzothiophene	2.24	-0.01	0.00	7	0.24	2.20	-1.48	0.18	2.52	-0.01	0.00	7	0.66	13.33	-3.65	0.01
dimethyldibenzothiophene	1.86	0.00	0.00	7	0.12	0.92	-0.96	0.37	2.13	0.00	0.00	7	0.36	3.95	-1.99	0.09
trimethyldibenzothiophene	1.75	0.00	0.00	7	0.16	1.37	-1.17	0.28	2.02	-0.01	0.00	7	0.44	5.41	-2.33	0.05
tetramethyldibenzothiophene	1.32	0.00	0.00	7	0.18	1.51	-1.23	0.26	1.58	-0.01	0.00	7	0.44	5.53	-2.35	0.05
phenanthrene	1.49	0.00	0.00	7	0.08	0.63	-0.79	0.45	1.75	0.00	0.00	7	0.33	3.46	-1.86	0.11
methylphenanthrene	2.20	0.00	0.00	7	0.10	0.82	-0.91	0.40	2.48	0.00	0.00	7	0.38	4.29	-2.07	0.08
dimethylphenanthrene	2.39	0.00	0.00	7	0.13	1.05	-1.03	0.34	2.66	-0.01	0.00	7	0.41	4.81	-2.19	0.06
trimethylphenanthrene	2.21	0.00	0.00	7	0.19	1.61	-1.27	0.24	2.49	-0.01	0.00	7	0.50	7.05	-2.66	0.03
tetramethylphenanthrene	1.77	0.00	0.00	7	0.14	1.11	-1.05	0.33	2.04	-0.01	0.00	7	0.51	7.35	-2.71	0.03
fluoranthene	-0.90	0.00	0.00	7	0.39	4.56	-2.14	0.07	-0.59	-0.01	0.00	7	0.51	7.38	-2.72	0.03
pyrene	-0.63	0.00	0.00	7	0.26	2.41	-1.55	0.16	-0.12	-0.01	0.00	7	0.65	12.72	-3.57	0.01
methylpyrene	0.79	-0.01	0.00	7	0.30	2.96	-1.72	0.13	1.10	-0.01	0.00	7	0.62	11.24	-3.35	0.01
dimethylpyrene	0.79	-0.01	0.00	7	0.25	2.30	-1.52	0.17	1.07	-0.01	0.00	7	0.65	12.93	-3.60	0.01
trimethylpyrene	0.74	-0.01	0.00	7	0.40	4.57	-2.14	0.07	1.03	-0.01	0.00	7	0.76	22.78	-4.77	0.00
tetramethylpyrene	0.83	-0.01	0.00	7	0.24	2.19	-1.48	0.18	1.17	-0.01	0.00	7	0.58	9.61	-3.10	0.02
naphthobenzothiophene	-0.90	0.00	0.00	7	0.29	2.92	-1.71	0.13	-0.68	0.00	0.00	7	0.40	4.62	-2.15	0.07
methylnaphthobenzothiophene	0.49	0.00	0.00	7	0.17	1.43	-1.20	0.27	0.74	0.00	0.00	7	0.40	4.76	-2.18	0.07
dimethylNBenzothiophene	0.80	0.00	0.00	7	0.22	2.00	-1.41	0.20	1.05	-0.01	0.00	7	0.49	6.83	-2.61	0.03
trimethylNbenzothiophene	0.62	0.00	0.00	7	0.24	2.23	-1.49	0.18	0.83	-0.01	0.00	7	0.40	4.71	-2.17	0.07
tetramethylNbenzothiophene	0.07	0.00	0.00	7	0.24	2.26	-1.50	0.18	0.40	-0.01	0.00	7	0.57	9.30	-3.05	0.02
benz[a]anthracene	-1.67	-0.01	0.00	7	0.68	14.72	-3.84	0.01	-1.65	-0.01	0.00	7	0.91	73.42	-8.57	0.00
chrysene	-0.76	0.00	0.00	7	0.11	0.89	-0.95	0.38	-0.48	0.00	0.00	7	0.38	4.33	-2.08	0.08
methylchrysene	-0.03	-0.01	0.00	7	0.27	2.55	-1.60	0.15	0.26	-0.01	0.00	7	0.62	11.25	-3.35	0.01
dimethylchrysene	0.36	-0.01	0.00	7	0.43	5.38	-2.32	0.05	0.67	-0.01	0.00	7	0.81	30.01	-5.48	0.00
trimethylchrysene	0.24	-0.01	0.00	7	0.48	6.38	-2.53	0.04	0.41	-0.01	0.00	7	0.73	19.23	-4.39	0.00
tetramethylchrysene	-0.17	-0.01	0.00	7	0.60	10.55	-3.25	0.01	0.00	-0.01	0.00	7	0.78	24.45	-4.94	0.00
benzo[b]fluoranthene	-1.36	0.00	0.00	7	0.72	18.26	-4.27	0.00	-1.21	0.00	0.00	7	0.50	6.94	-2.63	0.03
benzo[e]pyrene	-1.47	0.00	0.00	7	0.41	4.93	-2.22	0.06	-1.29	0.00	0.00	7	0.41	4.80	-2.19	0.06
benzo[ghi]perylene	-1.91	0.00	0.00	7	0.12	0.99	-0.99	0.35	-1.86	0.00	0.00	7	0.47	6.32	-2.51	0.04

CGR01	t-test for Significant difference between slopes				Rate constant (h-1)			Rate constant (d-1)	
Analyte	Error	t statistic	df	p-value	Abiotic	Live	Biodegradation	Abiotic	Biodegrada
C13					na	na	na		
C14					na	na	na		
C15					na	na	na		
C16					na	na	na		
C17					na	na	na		
C18					na	na	na		
C19					na	na	na		
C20					na	na	na		
C21					na	na	na		
C22					na	na	na		
C23					na	na	na		
C24					na	na	na		
C25					na	na	na		
C26					na	na	na		
C27					na	na	na		
C28					na	na	na		
C29					na	na	na		
C30					na	na	na		
C31					na	na	na		
C32					na	0.00	0.00		0.10
C33					na	na	na		
C34					na	-0.01	-0.01		0.22
C35	0.00	1.89	9.00	0.09	0.00	-0.01	na	0.10	
naphthalene					-0.01	na	na	0.13	
methylnaphthalene					na	na	na		
dimethylnaphthalene					-0.01	na	na	0.13	
trimethylnaphthalene					-0.01	na	na	0.18	
tetramethylnaphthalene					-0.01	na	na	0.22	
acenaphthene	0.00	-0.57	10.00	0.58	-0.01	-0.01	na	0.16	
fluorene					-0.01	na	na	0.18	
methylfluorene					-0.01	na	na	0.19	
dimethylfluorene					-0.01	na	na	0.23	
trimethylfluorene					-0.01	na	na	0.19	
dibenzothiophene					na	na	na		
methyldibenzothiophene					-0.01	na	na	0.21	
dimethyldibenzothiophene					na	na	na		
trimethyldibenzothiophene					na	na	na		
tetramethyldibenzothiophene					na	na	na		
phenanthrene					na	na	na		
methylphenanthrene					na	na	na		
dimethylphenanthrene					na	na	na		
trimethylphenanthrene					-0.01	na	na	0.15	
tetramethylphenanthrene					-0.01	na	na	0.15	
fluoranthene					-0.01	na	na	0.16	
pyrene					-0.01	na	na	0.26	
methylpyrene					-0.01	na	na	0.19	
dimethylpyrene					-0.01	na	na	0.20	
trimethylpyrene					-0.01	na	na	0.28	
tetramethylpyrene					-0.01	na	na	0.18	
naphthobenzothiophene					na	na	na		
methylnaphthobenzothiophene					na	na	na		
dimethylNBenzothiophene					-0.01	na	na	0.15	
trimethylNbenzothiophene					na	na	na		
tetramethylNbenzothiophene					-0.01	na	na	0.17	
benz[a]anthracene	0.00	-0.09	10.00	0.93	-0.01	-0.01	na	0.24	
chrysene					na	na	na		
methylchrysene					-0.01	na	na	0.20	
dimethylchrysene					-0.01	na	na	0.29	
trimethylchrysene	0.01	-0.02	10.00	0.99	-0.01	-0.01	na	0.30	
tetramethylchrysene	0.00	-0.22	10.00	0.83	-0.01	-0.01	na	0.25	
benzo[b]fluoranthene	0.00	-0.15	10.00	0.88	0.00	0.00	na	0.10	
benzo[e]pyrene					na	na	na		
benzo[ghi]perylene					0.00	na	na	0.07	

Table S6 Linear regression statistics for the live and killed control incubations from CGR11. For each treatment, a t-test was conducted to determine if the slope was significantly different from zero. A second t-test compared the slope from the killed control incubations to those of the live incubations. The estimated rate constant is calculated for abiotic degradation or biodegradation.

CGR11	Linear Regressions																		t-test for Significant difference between slopes					Rate constant (h-1)				Rate constant (d-1)	
	Live									Killed Control																			
Analyte	Intercept	Slope	SlopeSE	df	R2	Fstat	t-value	p-value		Intercept	Slope	SlopeSE	df	R2	Fstat	t-value	p-value		Error	t statistic	df	p-value		Abiotic	Live	Biodegradation		Abiotic	Biodegrada
C13	2.33	0.00	0.00	7	0.25	2.36	-1.54	0.17		2.38	0.00	0.00	6	0.46	5.05	-2.25	0.07							na	na	na			
C14	2.45	0.00	0.00	7	0.42	5.01	-2.24	0.06		2.42	0.00	0.00	6	0.72	15.61	-3.95	0.01							0.00	na	na		0.09	
C15	2.36	0.00	0.00	7	0.50	6.99	-2.64	0.03		2.54	-0.01	0.00	6	0.65	11.39	-3.37	0.01		0.00	-1.04	9	0.33		-0.01	0.00	na		0.13	
C16	2.48	0.00	0.00	7	0.55	8.49	-2.91	0.02		2.41	0.00	0.00	6	0.08	0.51	-0.71	0.50							na	0.00	0.00			0.06
C17	2.62	0.00	0.00	7	0.30	3.02	-1.74	0.13		2.64	0.00	0.00	6	0.20	1.53	-1.24	0.26							na	na	na			
C18	2.28	0.00	0.00	7	0.49	6.86	-2.62	0.03		2.29	0.00	0.00	6	0.43	4.44	-2.11	0.08							na	0.00	0.00			0.05
C19	2.23	0.00	0.00	7	0.48	6.53	-2.56	0.04		2.21	0.00	0.00	6	0.62	9.94	-3.15	0.02		0.00	0.63	9	0.54		0.00	0.00	na		0.04	
C20	2.09	0.00	0.00	7	0.62	11.64	-3.41	0.01		2.09	0.00	0.00	6	0.63	10.17	-3.19	0.02		0.00	0.16	9	0.87		0.00	0.00	na		0.06	
C21	2.00	0.00	0.00	7	0.55	8.50	-2.92	0.02		2.00	0.00	0.00	6	0.64	10.83	-3.29	0.02		0.00	0.14	9	0.89		0.00	0.00	na		0.06	
C22	2.06	0.00	0.00	7	0.51	7.29	-2.70	0.03		2.00	0.00	0.00	6	0.70	14.04	-3.75	0.01		0.00	0.69	9	0.50		0.00	0.00	na		0.08	
C23	2.02	0.00	0.00	7	0.50	7.06	-2.66	0.03		1.99	0.00	0.00	6	0.70	13.79	-3.71	0.01		0.00	0.46	9	0.65		0.00	0.00	na		0.09	
C24	2.07	0.00	0.00	7	0.48	6.43	-2.53	0.04		2.21	-0.01	0.00	6	0.88	42.81	-6.54	0.00		0.00	-1.02	9	0.33		-0.01	0.00	na		0.17	
C25	1.90	0.00	0.00	7	0.50	6.97	-2.64	0.03		2.04	-0.01	0.00	6	0.89	46.97	-6.85	0.00		0.00	-0.94	9	0.37		-0.01	0.00	na		0.16	
C26	1.93	0.00	0.00	7	0.55	8.72	-2.95	0.02		2.04	-0.01	0.00	6	0.79	22.68	-4.76	0.00		0.00	-0.55	9	0.59		-0.01	0.00	na		0.15	
C27	1.71	0.00	0.00	7	0.13	1.05	-1.02	0.34		1.99	-0.01	0.00	6	0.81	26.42	-5.14	0.00							-0.01	na	na		0.17	
C28	1.63	0.00	0.00	7	0.23	2.09	-1.45	0.19		1.86	-0.01	0.00	6	0.89	47.57	-6.90	0.00							-0.01	na	na		0.17	
C29	1.70	-0.01	0.00	7	0.62	11.61	-3.41	0.01		1.81	-0.01	0.00	6	0.85	34.73	-5.89	0.00		0.00	-0.57	9	0.59		-0.01	-0.01	na		0.19	
C30	0.88	0.00	0.00	7	0.31	3.11	-1.76	0.12		1.11	-0.01	0.00	6	0.46	5.07	-2.25	0.07							na	na	na			
C31	0.75	0.00	0.00	7	0.28	2.66	-1.63	0.15		1.02	-0.01	0.00	6	0.85	33.41	-5.78	0.00							-0.01	na	na		0.14	
C32	0.34	0.00	0.00	7	0.35	3.70	-1.92	0.10		0.55	0.00	0.00	6	0.66	11.44	-3.38	0.01							0.00	na	na		0.12	
C33	0.26	0.00	0.00	7	0.35	3.81	-1.95	0.09		0.35	0.00	0.00	6	0.58	8.41	-2.90	0.03							0.00	na	na		0.09	
C34	0.02	0.00	0.00	7	0.17	1.42	-1.19	0.27		0.19	0.00	0.00	5	0.72	12.63	-3.55	0.02							0.00	na	na		0.11	
C35	-0.18	0.00	0.00	7	0.15	1.24	-1.11	0.30		-0.14	0.00	0.00	4	0.08	0.33	-0.57	0.60							na	na	na			
naphthalene	2.02	0.00	0.00	7	0.18	1.57	-1.25	0.25		2.04	0.00	0.00	6	0.47	5.32	-2.31	0.06							na	na	na			
methylnaphthalene	2.89	0.00	0.00	7	0.40	4.63	-2.15	0.07		2.95	0.00	0.00	6	0.68	12.89	-3.59	0.01							0.00	na	na		0.12	
dimethylnaphthalene	3.56	-0.01	0.00	7	0.64	12.41	-3.52	0.01		3.58	-0.01	0.00	6	0.86	36.90	-6.07	0.00		0.00	-0.33	9	0.75		-0.01	-0.01	na		0.14	
trimethylnaphthalene	3.53	-0.01	0.00	7	0.88	52.20	-7.22	0.00		3.56	-0.01	0.00	6	0.95	104.22	-10.21	0.00		0.00	-0.22	9	0.83		-0.01	-0.01	na		0.22	
tetramethylnaphthalene	3.18	-0.01	0.00	7	0.92	84.37	-9.19	0.00		3.19	-0.01	0.00	6	0.97	179.27	-13.39	0.00		0.00	-0.36	9	0.73		-0.01	-0.01	na		0.25	
acenaphthene	-0.85	-0.01	0.00	7	0.77	22.99	-4.80	0.00		-0.80	-0.01	0.00	6	0.90	52.20	-7.22	0.00		0.00	-0.95	9	0.37		-0.01	-0.01	na		0.20	
fluorene	1.30	-0.01	0.00	7	0.85	38.67	-6.22	0.00		1.26	-0.01	0.00	6	0.93	83.36	-9.13	0.00		0.00	-0.26	9	0.80		-0.01	-0.01	na		0.18	
methylfluorene	1.85	-0.01	0.00	7	0.79	27.01	-5.20	0.00		1.83	-0.01	0.00	6	0.90	56.01	-7.48	0.00		0.00	-0.63	9	0.54		-0.01	-0.01	na		0.17	
dimethylfluorene	2.05	-0.01	0.00	7	0.85	38.96	-6.24	0.00		2.01	-0.01	0.00	6	0.91	59.51	-7.71	0.00		0.00	-0.36	9	0.72		-0.01	-0.01	na		0.21	
trimethylfluorene	2.05	-0.01	0.00	7	0.80	27.96	-5.29	0.00		1.99	-0.01	0.00	6	0.93	83.06	-9.11	0.00		0.00	0.54	9	0.60		-0.01	-0.01	na		0.14	
dibenzothiophene	0.93	0.00	0.00	7	0.56	8.87	-2.98	0.02		0.93	0.00	0.00	6	0.75	17.98	-4.24	0.01		0.00	-0.07	9	0.95		0.00	0.00	na		0.08	
methyldibenzothiophene	2.70	-0.01	0.00	7	0.76	21.91	-4.68	0.00		2.74	-0.01	0.00	6	0.95	112.61	-10.61	0.00		0.00	-0.64	9	0.54		-0.01	-0.01	na		0.18	
dimethyldibenzothiophene	2.35	0.00	0.00	7	0.53	8.04	-2.84	0.03		2.36	0.00	0.00	6	0.82	27.78	-5.27	0.00		0.00	0.13	9	0.90		0.00	0.00	na		0.07	
trimethyldibenzothiophene	2.28	0.00	0.00	7	0.62	11.50	-3.39	0.01		2.30	0.00	0.00	6	0.90	54.23	-7.36	0.00		0.00	0.08	9	0.94		0.00	0.00	na		0.09	
tetramethyldibenzothiophene	1.87	0.00	0.00	7	0.60	10.31	-3.21	0.01		1.87	0.00	0.00	6	0.72	15.78	-3.97	0.01		0.00	0.14	9	0.90		0.00	0.00	na		0.09	
phenanthrene	1.92	0.00	0.00	7	0.37	4.06	-2.02	0.08		1.94	0.00	0.00	6	0.58	8.20	-2.86	0.03							0.00	na	na		0.06	
methylphenanthrene	2.68	0.00	0.00	7	0.54	8.07	-2.84	0.03		2.68	0.00	0.00	6	0.79	22.02	-4.69	0.00		0.00	0.02	9	0.98		0.00	0.00	na		0.07	
dimethylphenanthrene	2.89	0.00	0.00	7	0.63	11.96	-3.46	0.01		2.91	0.00	0.00	6	0.88	42.45	-6.52	0.00		0.00	-0.03	9	0.98		0.00	0.00	na		0.09	
trimethylphenanthrene	2.76	0.00	0.00	7	0.71	17.49	-4.18	0.00		2.78	-0.01	0.00	6	0.94	87.84	-9.37	0.00		0.00	-0.29	9	0.78		-0.01	0.00	na		0.12	
tetramethylphenanthrene	2.34	0.00	0.00	7	0.59	10.19	-3.19	0.02		2.32	0.00	0.00	6	0.71	14.75	-3.84	0.01		0.00	0.32	9	0.75		0.00	0.00	na		0.10	
fluoranthene	-0.32	-0.01	0.00	7	0.93	91.83	-9.58	0.00		-0.27	-0.01	0.00	6	0.92	73.40	-8.57	0.00		0.00	-0.29	9	0.78		-0.01	-0.01	na		0.17	
pyrene	0.10	-0.01	0.00	7	0.87	45.85	-6.77	0.00		0.12	-0.01	0.00	6	0.97	216.80	-14.72	0.00		0.00	-0.28	9	0.78		-0.01	-0.01	na		0.20	
methylpyrene	1.39	-0.01	0.00	7	0.74	19.79	-4.45	0.00		1.37	-0.01	0.00	6	0.92	65.76	-8.11	0.00		0.00	0.24	9	0.81		-0.01	-0.01	na		0.14	
dimethylpyrene	1.34	-0.01	0.00	7	0.82	30.90	-5.56	0.00		1.36	-0.01	0.00	6	0.97	177.79	-13.33	0.00		0.00	-0.38	9	0.71		-0.01	-0.01	na		0.18	
trimethylpyrene	1.33	-0.01	0.00	7	0.87	48.34	-6.95	0.00		1.41	-0.01	0.00	6	0.95	108.07	-10.40	0.00		0.00	-0.78	9	0.45		-0.01	-0.01	na		0.27	
tetramethylpyrene	1.46	-0.01	0.00	7	0.70	16.19	-4.02	0.01		1.49	-0.01	0.00	6	0.90	56.26	-7.50	0.00		0.00	-0.49	9	0.64		-0.01	-0.01	na		0.18	
naphthobenzothiophene	-0.63	0.00	0.00	7	0.36	3.90	-1.98	0.09		-0.58	0.00	0.00	6	0.75	18.13	-4.26	0.01							0.00	na	na		0.08	
methylnaphthobenzothiophene	0.89	0.00	0.00	7	0.61	10.77	-3.28	0.01		0.93	0.00	0.00	6	0.89	50.36	-7.10	0.00		0.00	-0.41	9	0.69		0.00	0.00	na		0.09	
dimethylNBenzothiophene	1.19	0.00	0.00	7	0.63	11.80	-3.43	0.01		1.23	0.00	0.00	6	0.95	107.07	-10.35	0.00		0.00	-0.55	9	0.59		0.00	0.00	na		0.10	
trimethylNbenzothiophene	1.01	0.00	0.00	7	0.56	9.08	-3.01	0.02		1.06	0.00	0.00	6	0.94	89.10	-9.44	0.00		0.00	-0.54	9	0.60		0.00	0.00	na		0.11	
tetramethylNbenzothiophene	0.50	0.00	0.00	7	0.58	9.48	-3.08	0.02		0.52	-0.01	0.00	6	0.91	61.06	-7.81	0.00		0.00	-0.63	9	0.55		-0.01	0.00	na		0.12	
benz[a]anthracene	-1.93	-0.01	0.00	7	0.82	31.98	-5.66	0.00		-1.94	-0.01	0.00	6	0.93	82.77	-9.10	0.00		0.00	-1.54	9	0.16		-0.01	-0.01	na		0.35	
chrysene	-0.31	0.00	0.00	7	0.66	13.72	-3.70	0.01		-0.26	0.00	0.00	6	0.90	52.49	-7.24	0.00		0.00	-0.53	9	0.61		0.00	0.00	na		0.10	
methylchrysene	0.45	-0.01	0.00	7	0.84	35.82	-5.99	0.00		0.50	-0.01	0.00	6	0.96	149.96	-12.25	0.00		0.00	-0.94	9	0.37		-0.01	-0.01	na		0.19	
dimethylchrysene	0.83	-0.01	0.00	7	0.92	78.93	-8.88	0.00		0.82	-0.01	0.00	6	0.99	632.41	-25.15	0.00		0.00	-0.63	9	0.54		-0.01	-0.01	na		0.27	
trimethylchrysene	0.63	-0.01	0.00	7	0.85	40.14	-6.34	0.00		0.61	-0.01	0.00	6	0.97	176.13	-13.27	0.00		0.00	-0.30	9	0.77		-0.01	-0.01	na		0.25	
tetramethylchrysene	0.32	-0.01	0.00	7	0.67	13.94	-3.73	0.01		0.35	-0.01	0.00	6	0.95	117.70	-10.85	0.00		0.00	-0.72	9	0.49		-0.01	-0.01	na		0.28	
benzo[b]fluoranthene	-1.26	0.00	0.00	7	0.54	8.07	-2.84	0.02		-1.19	0.00	0.00	6	0.86	38.12	-6.17	0.00		0.00	-0.64	9	0.54		0.00	0.00	na		0.11	
benzo[e]pyrene	-1.22	0.00	0.00	7	0.33	3.47	-1.86	0.10		-1.17	0.00	0.00	6	0.70	13.72	-3.70	0.01							0.00	na	na		0.08	
benzo[ghi]perylene	-2.18	0.00	0.00	7	0.58	9.65	-3.11	0.02		-2.15	0.00	0.00	6	0.81	25.04	-5.00	0.00		0.00	-0.12	9	0.90		0.00	0.00	na		0.10	

Table S7 Linear regression statistics for the live and killed control incubations from CGR22. For each treatment, a t-test was conducted to determine if the slope was significantly different from zero. A second t-test compared the slope from the killed control incubations to those of the live incubations. The estimated rate constant is calculated for abiotic degradation or biodegradation.

CGR22	Linear Regressions															
	Live								Killed Control							
Analyte	Intercept	Slope	SlopeSE	df	R2	Fstat	t-value	p-value	Intercept	Slope	SlopeSE	df	R2	Fstat	t-value	p-value
C13	2.24	0.00	0.00	7	0.08	0.65	-0.80	0.45	2.29	0.00	0.00	7	0.43	5.36	-2.32	0.05
C14	2.29	0.00	0.00	7	0.13	1.01	-1.01	0.35	2.34	0.00	0.00	7	0.32	3.23	-1.80	0.12
C15	2.30	0.00	0.00	7	0.13	1.04	-1.02	0.34	2.38	0.00	0.00	7	0.43	5.31	-2.30	0.05
C16	2.34	0.00	0.00	7	0.11	0.84	-0.92	0.39	2.39	0.00	0.00	7	0.35	3.77	-1.94	0.09
C17	2.49	0.00	0.00	7	0.29	2.81	-1.68	0.14	2.48	0.00	0.00	7	0.22	2.03	-1.42	0.20
C18	2.13	0.00	0.00	7	0.11	0.83	-0.91	0.39	2.20	0.00	0.00	7	0.32	3.30	-1.82	0.11
C19	2.10	0.00	0.00	7	0.22	2.03	-1.42	0.20	2.15	0.00	0.00	7	0.26	2.41	-1.55	0.16
C20	1.94	0.00	0.00	7	0.15	1.22	-1.11	0.30	1.95	0.00	0.00	7	0.42	5.04	-2.25	0.06
C21	1.84	0.00	0.00	7	0.11	0.85	-0.92	0.39	1.89	0.00	0.00	7	0.46	5.90	-2.43	0.05
C22	1.80	0.00	0.00	7	0.12	0.96	-0.98	0.36	1.86	0.00	0.00	7	0.47	6.29	-2.51	0.04
C23	1.76	0.00	0.00	7	0.14	1.10	-1.05	0.33	1.79	0.00	0.00	7	0.41	4.85	-2.20	0.06
C24	1.80	0.00	0.00	7	0.17	1.41	-1.19	0.27	1.84	0.00	0.00	7	0.47	6.23	-2.50	0.04
C25	1.65	0.00	0.00	7	0.14	1.12	-1.06	0.32	1.69	0.00	0.00	7	0.47	6.24	-2.50	0.04
C26	1.63	0.00	0.00	7	0.14	1.17	-1.08	0.32	1.65	0.00	0.00	7	0.38	4.35	-2.09	0.08
C27	1.53	0.00	0.00	7	0.21	1.86	-1.36	0.22	1.54	0.00	0.00	7	0.36	3.96	-1.99	0.09
C28	1.44	0.00	0.00	7	0.18	1.50	-1.22	0.26	1.51	0.00	0.00	7	0.58	9.71	-3.12	0.02
C29	1.41	0.00	0.00	7	0.31	3.18	-1.78	0.12	1.42	0.00	0.00	7	0.43	5.29	-2.30	0.06
C30	0.61	0.00	0.00	7	0.03	0.25	-0.50	0.63	0.65	0.00	0.00	7	0.39	4.54	-2.13	0.07
C31	0.53	0.00	0.00	7	0.12	0.96	-0.98	0.36	0.55	0.00	0.00	7	0.37	4.05	-2.01	0.08
C32	0.22	0.00	0.00	7	0.12	0.93	-0.97	0.37	0.24	0.00	0.00	7	0.30	2.95	-1.72	0.13
C33	0.09	0.00	0.00	7	0.18	1.58	-1.26	0.25	0.08	0.00	0.00	7	0.34	3.68	-1.92	0.10
C34	-0.06	0.00	0.00	7	0.29	2.92	-1.71	0.13	-0.19	0.00	0.00	7	0.00	0.02	0.13	0.90
C35	-0.28	0.00	0.00	7	0.25	2.29	-1.51	0.17	-0.37	0.00	0.00	7	0.08	0.55	-0.74	0.48
naphthalene	1.92	0.00	0.00	7	0.09	0.72	-0.85	0.43	1.95	0.00	0.00	7	0.48	6.38	-2.53	0.04
methylnaphthalene	2.71	0.00	0.00	7	0.14	1.14	-1.07	0.32	2.75	0.00	0.00	7	0.57	9.38	-3.06	0.02
dimethylnaphthalene	3.30	0.00	0.00	7	0.23	2.07	-1.44	0.19	3.35	0.00	0.00	7	0.66	13.66	-3.70	0.01
trimethylnaphthalene	3.19	-0.01	0.00	7	0.45	5.64	-2.37	0.05	3.23	-0.01	0.00	7	0.76	21.68	-4.66	0.00
tetramethylnaphthalene	2.75	-0.01	0.00	7	0.42	5.11	-2.26	0.06	2.80	-0.01	0.00	7	0.79	26.11	-5.11	0.00
acenaphthene	-1.01	-0.01	0.00	7	0.55	8.47	-2.91	0.02	-1.00	-0.01	0.00	7	0.82	31.18	-5.58	0.00
fluorene	1.00	0.00	0.00	7	0.40	4.60	-2.15	0.07	1.01	-0.01	0.00	7	0.73	19.27	-4.39	0.00
methylfluorene	1.62	-0.01	0.00	7	0.41	4.86	-2.20	0.06	1.66	-0.01	0.00	7	0.78	24.94	-4.99	0.00
dimethylfluorene	1.75	-0.01	0.00	7	0.46	5.93	-2.43	0.05	1.79	-0.01	0.00	7	0.84	37.91	-6.16	0.00
trimethylfluorene	1.72	-0.01	0.00	7	0.43	5.31	-2.30	0.05	1.77	-0.01	0.00	7	0.83	34.80	-5.90	0.00
dibenzothiophene	0.69	0.00	0.00	7	0.09	0.73	-0.85	0.42	0.75	0.00	0.00	7	0.47	6.27	-2.50	0.04
methyldibenzothiophene	2.45	-0.01	0.00	7	0.36	4.01	-2.00	0.09	2.49	-0.01	0.00	7	0.73	18.72	-4.33	0.00
dimethyldibenzothiophene	2.04	0.00	0.00	7	0.08	0.61	-0.78	0.46	2.11	0.00	0.00	7	0.46	6.04	-2.46	0.04
trimethyldibenzothiophene	1.94	0.00	0.00	7	0.13	1.04	-1.02	0.34	1.97	0.00	0.00	7	0.47	6.20	-2.49	0.04
tetramethyldibenzothiophene	1.48	0.00	0.00	7	0.10	0.74	-0.86	0.42	1.48	0.00	0.00	7	0.28	2.77	-1.66	0.14
phenanthrene	1.74	0.00	0.00	7	0.05	0.33	-0.58	0.58	1.77	0.00	0.00	7	0.27	2.56	-1.60	0.15
methylphenanthrene	2.44	0.00	0.00	7	0.08	0.57	-0.75	0.47	2.48	0.00	0.00	7	0.39	4.42	-2.10	0.07
dimethylphenanthrene	2.61	0.00	0.00	7	0.10	0.77	-0.88	0.41	2.65	0.00	0.00	7	0.43	5.36	-2.31	0.05
trimethylphenanthrene	2.43	0.00	0.00	7	0.15	1.28	-1.13	0.30	2.47	0.00	0.00	7	0.60	10.45	-3.23	0.01
tetramethylphenanthrene	1.98	0.00	0.00	7	0.12	0.92	-0.96	0.37	1.95	0.00	0.00	7	0.27	2.65	-1.63	0.15
fluoranthene	-0.66	0.00	0.00	7	0.18	1.54	-1.24	0.25	-0.59	-0.01	0.00	7	0.66	13.86	-3.72	0.01
pyrene	-0.34	0.00	0.00	7	0.24	2.24	-1.50	0.18	-0.35	0.00	0.00	7	0.52	7.73	-2.78	0.03
methylpyrene	1.07	0.00	0.00	7	0.29	2.81	-1.68	0.14	1.10	-0.01	0.00	7	0.68	15.01	-3.87	0.01
dimethylpyrene	1.03	0.00	0.00	7	0.36	3.88	-1.97	0.09	1.04	-0.01	0.00	7	0.71	16.80	-4.10	0.00
trimethylpyrene	0.92	-0.01	0.00	7	0.46	5.92	-2.43	0.05	1.01	-0.01	0.00	7	0.82	31.63	-5.62	0.00
tetramethylpyrene	1.08	-0.01	0.00	7	0.30	3.04	-1.74	0.12	1.18	-0.01	0.00	7	0.59	10.27	-3.20	0.01
naphthobenzothiophene	-0.82	0.00	0.00	7	0.13	1.05	-1.03	0.34	-0.80	0.00	0.00	7	0.39	4.40	-2.10	0.07
methylnaphthobenzothiophene	0.64	0.00	0.00	7	0.13	1.08	-1.04	0.33	0.68	0.00	0.00	7	0.52	7.70	-2.78	0.03
dimethylNBenzothiophene	0.96	0.00	0.00	7	0.19	1.67	-1.29	0.24	1.01	0.00	0.00	7	0.60	10.63	-3.26	0.01
trimethylNbenzothiophene	0.73	0.00	0.00	7	0.13	1.00	-1.00	0.35	0.85	-0.01	0.00	7	0.64	12.32	-3.51	0.01
tetramethylNbenzothiophene	0.27	-0.01	0.00	7	0.39	4.44	-2.11	0.07	0.25	-0.01	0.00	7	0.66	13.73	-3.71	0.01
benz[a]anthracene	-1.99	-0.01	0.00	7	0.93	89.32	-9.45	0.00	-1.99	-0.01	0.00	6	0.91	63.46	-7.97	0.00
chrysene	-0.51	0.00	0.00	7	0.15	1.22	-1.10	0.31	-0.47	0.00	0.00	7	0.54	8.27	-2.88	0.02
methylchrysene	0.20	-0.01	0.00	7	0.42	4.99	-2.23	0.06	0.25	-0.01	0.00	7	0.80	27.37	-5.23	0.00
dimethylchrysene	0.51	-0.01	0.00	7	0.64	12.40	-3.52	0.01	0.55	-0.01	0.00	7	0.86	44.77	-6.69	0.00
trimethylchrysene	0.49	-0.01	0.00	7	0.68	14.74	-3.84	0.01	0.47	-0.01	0.00	7	0.87	47.78	-6.91	0.00
tetramethylchrysene	0.06	-0.01	0.00	7	0.67	14.16	-3.76	0.01	0.06	-0.01	0.00	7	0.94	119.62	-10.94	0.00
benzo[b]fluoranthene	-1.49	0.00	0.00	7	0.12	0.96	-0.98	0.36	-1.47	0.00	0.00	7	0.40	4.65	-2.16	0.07
benzo[e]pyrene	-1.40	0.00	0.00	7	0.11	0.85	-0.92	0.39	-1.41	0.00	0.00	7	0.35	3.69	-1.92	0.10
benzo[ghi]perylene	-2.43	0.00	0.00	6	0.62	9.76	-3.12	0.02	-2.56	0.00	0.00	6	0.30	2.57	-1.60	0.16

CGR22	t-test for Significant difference between slopes				Rate constant (h-1)			Rate constant (d-1)	
Analyte	Error	t statistic	df	p-value	Abiotic	Live	Biodegradation	Abiotic	Biodegrada
C13					na	na	na		
C14					na	na	na		
C15					na	na	na		
C16					na	na	na		
C17					na	na	na		
C18					na	na	na		
C19					na	na	na		
C20					na	na	na		
C21					0.00	na	na	0.06	
C22					0.00	na	na	0.07	
C23					na	na	na		
C24					0.00	na	na	0.07	
C25					0.00	na	na	0.07	
C26					na	na	na		
C27					na	na	na		
C28					0.00	na	na	0.09	
C29					na	na	na		
C30					na	na	na		
C31					na	na	na		
C32					na	na	na		
C33					na	na	na		
C34					na	na	na		
C35					na	na	na		
naphthalene					0.00	na	na	0.07	
methylnaphthalene					0.00	na	na	0.09	
dimethylnaphthalene					0.00	na	na	0.11	
trimethylnaphthalene	0.00	-0.61	10	0.55	-0.01	-0.01	na	0.18	
tetramethylnaphthalene					-0.01	na	na	0.20	
acenaphthene	0.00	-0.62	10	0.55	-0.01	-0.01	na	0.16	
fluorene					-0.01	na	na	0.15	
methylfluorene					-0.01	na	na	0.18	
dimethylfluorene	0.00	-1.25	10	0.24	-0.01	-0.01	na	0.25	
trimethylfluorene					-0.01	na	na	0.19	
dibenzothiophene					0.00	na	na	0.09	
methyldibenzothiophene					-0.01	na	na	0.18	
dimethyldibenzothiophene					0.00	na	na	0.08	
trimethyldibenzothiophene					0.00	na	na	0.08	
tetramethyldibenzothiophene					na	na	na		
phenanthrene					na	na	na		
methylphenanthrene					na	na	na		
dimethylphenanthrene					na	na	na		
trimethylphenanthrene					0.00	na	na	0.11	
tetramethylphenanthrene					na	na	na		
fluoranthene					-0.01	na	na	0.12	
pyrene					0.00	na	na	0.11	
methylpyrene					-0.01	na	na	0.13	
dimethylpyrene					-0.01	na	na	0.15	
trimethylpyrene	0.00	-1.28	10	0.23	-0.01	-0.01	na	0.24	
tetramethylpyrene					-0.01	na	na	0.17	
naphthobenzothiophene					na	na	na		
methylnaphthobenzothiophene					0.00	na	na	0.09	
dimethylNBenzothiophene					0.00	na	na	0.11	
trimethylNbenzothiophene					-0.01	na	na	0.14	
tetramethylNbenzothiophene					-0.01	na	na	0.14	
benz[a]anthracene	0.00	-2.18	9	0.06	-0.01	-0.01	na	0.30	
chrysene					0.00	na	na	0.09	
methylchrysene					-0.01	na	na	0.18	
dimethylchrysene	0.00	-0.94	10	0.37	-0.01	-0.01	na	0.26	
trimethylchrysene	0.00	-0.23	10	0.82	-0.01	-0.01	na	0.35	
tetramethylchrysene	0.00	-0.46	10	0.66	-0.01	-0.01	na	0.27	
benzo[b]fluoranthene					na	na	na		
benzo[e]pyrene					na	na	na		
benzo[ghi]perylene					na	0.00	0.00		0.09

Table S8 Initial biodegradation rate constants from the six experiments (d-1) for each compound. For details of the regressions and t-tests, see Tables S2-7. Summary data reports the mean and standard deviation based on the number of analytes for which a rate constant could be estimated.

Analyte	Stn01	Stn11	Stn20	CGR01	CGR11	CGR22
Number of *n*-alkanes	17	0	2	2	2	0
Average (sd) rates - *n*-alkanes	0.10 (0.02)		0.09 (0.02)	0.16 (0.08)	0.06 (0.00)	
Number of PACs	30	1	8	0	0	1
Average (sd) rates - PACs	0.12 (0.04)	0.11	0.08 (0.02)			0.09
C13	0.11					
C14	0.10					
C15	0.09					
C16	0.10				0.06	
C17			0.11			
C18	0.10				0.05	
C19	0.09					
C20	0.08					
C21	0.09					
C22	0.08					
C23	0.08					
C24	0.09					
C25	0.08					
C26	0.10					
C27	0.09					
C28	0.11					
C29						
C30						
C31						
C32				0.10		
C33	0.16		0.08			
C34	0.12			0.22		
C35						
naphthalene	0.08		0.10			
methylnaphthalene						
dimethylnaphthalene	0.11					
trimethylnaphthalene	0.12					
tetramethylnaphthalene	0.12					
acenaphthene	0.13					
fluorene	0.12					
methylfluorene	0.15					
dimethylfluorene	0.14					
trimethylfluorene	0.12					
dibenzothiophene	0.08					

methyldibenzothiophene	0.11					
dimethyldibenzothiophene			0.07			
trimethyldibenzothiophene	0.10		0.07			
tetramethyldibenzothiophene	0.10					
phenanthrene	0.08		0.06			
methylphenanthrene	0.08		0.07			
dimethylphenanthrene						
trimethylphenanthrene	0.10					
tetramethylphenanthrene	0.10					
fluoranthene						
pyrene						
methylpyrene	0.30					
dimethylpyrene	0.16					
trimethylpyrene			0.11			
tetramethylpyrene	0.17	0.11				
naphthobenzothiophene	0.08					
methylnaphthobenzothiophene			0.08			
dimethylNBenzothiophene			0.08			
trimethylNbenzothiophene	0.11					
tetramethylNbenzothiophene	0.10					
benz[a]anthracene						
chrysene	0.09					
methylchrysene	0.11					
dimethylchrysene	0.12					
trimethylchrysene						
tetramethylchrysene	0.17					
benzo[b]fluoranthene	0.08					
benzo[e]pyrene	0.16					
benzo[ghi]perylene	0.08					0.09

Table S9 Oxygen concentration in live seawater with oil initially and after 72 h at 22°C for three incubations.

Incubation	Treatment	O2 mg/mL - 0 h	O2 mg/mL - 72 h
Light	Live	7.84	13.19
Light	Live	6.78	12.83
Light	Live	7.44	12.18

Table S10 Initial abiotic degradation rate constants from the six experiments (d-1) for each compound. For details of the regressions and t-tests, see Tables S2-7. Summary data reports the mean and standard deviation based on the number of analytes for which a rate constant could be estimated.

**Analyte**	**Stn01**	**Stn11**	**Stn20**	**CGR01**	**CGR11**	**CGR22**
Number of *n*-alkanes	20	23	17	1	17	5
Average (sd) rates - *n*-alkanes	0.10 (0.02)	0.09 (0.02)	0.06 (0.01)	0.09	0.12 (0.04)	0.07 (0.01)
Number of PACs	37	39	31	27	39	31
Average (sd) rates - PACs	0.15 (0.05)	0.11 (0.05)	0.11 (0.06)	0.19 (0.06)	0.15 (0.07)	0.16 (0.07)
C13	0.06	0.08	0.06			
C14	0.09	0.08	0.06		0.09	
C15	0.09	0.08	0.07		0.13	
C16	0.09	0.07	0.07			
C17	0.16	0.08				
C18	0.08	0.07	0.04			
C19	0.11	0.08	0.06		0.04	
C20	0.11	0.08	0.06		0.06	
C21	0.09	0.07	0.06		0.06	0.06
C22	0.10	0.08	0.06		0.08	0.07
C23	0.09	0.07	0.06		0.09	
C24	0.10	0.08	0.07		0.17	0.07
C25	0.11	0.08	0.07		0.16	0.07
C26	0.09	0.08	0.06		0.15	
C27	0.10	0.09	0.05		0.17	
C28	0.10	0.10	0.07		0.17	0.09
C29	0.15	0.12	0.08		0.19	
C30	0.10	0.10				
C31	0.07	0.09	0.05		0.14	
C32	0.08	0.06			0.12	
C33		0.12			0.09	
C34		0.14			0.11	
C35		0.10		0.09		
naphthalene	0.09	0.07		0.13		0.07
methylnaphthalene	0.10	0.08	0.06		0.12	0.09
dimethylnaphthalene	0.11	0.09	0.08	0.13	0.14	0.11
trimethylnaphthalene	0.15	0.13	0.12	0.18	0.22	0.18
tetramethylnaphthalene	0.18	0.16	0.16	0.22	0.25	0.20
acenaphthene	0.16	0.13	0.12	0.16	0.20	0.16
fluorene	0.20	0.10	0.11	0.18	0.18	0.15
methylfluorene	0.19	0.11	0.12	0.19	0.17	0.18
dimethylfluorene	0.22	0.16	0.15	0.23	0.21	0.25
trimethylfluorene	0.20	0.12	0.12	0.19	0.14	0.19

dibenzothiophene	0.11	0.08	0.07		0.08	0.09
methyldibenzothiophene	0.17	0.14	0.14	0.21	0.18	0.18
dimethyldibenzothiophene	0.11	0.06			0.07	0.08
trimethyldibenzothiophene	0.10	0.06			0.09	0.08
tetramethyldibenzothiophene	0.11	0.05			0.09	
phenanthrene	0.09	0.05			0.06	
methylphenanthrene	0.11	0.06			0.07	
dimethylphenanthrene	0.14	0.07	0.05		0.09	
trimethylphenanthrene	0.14	0.10	0.06	0.15	0.12	0.11
tetramethylphenanthrene	0.13	0.12	0.07	0.15	0.10	
fluoranthene		0.10	0.07	0.16	0.17	0.12
pyrene	0.22	0.08	0.07	0.26	0.20	0.11
methylpyrene		0.09	0.07	0.19	0.14	0.13
dimethylpyrene	0.16	0.12	0.09	0.20	0.18	0.15
trimethylpyrene	0.23	0.15		0.28	0.27	0.24
tetramethylpyrene	0.21		0.14	0.18	0.18	0.17
naphthobenzothiophene	0.10	0.09	0.08		0.08	
methylnaphthobenzothiophene	0.12	0.07			0.09	0.09
dimethylNBenzothiophene	0.12	0.07		0.15	0.10	0.11
trimethylNbenzothiophene	0.12	0.09	0.09		0.11	0.14
tetramethylNbenzothiophene	0.12	0.09	0.09	0.17	0.12	0.14
benz[a]anthracene	0.16	0.22	0.26	0.24	0.35	0.30
chrysene	0.12	0.08	0.07		0.10	0.09
methylchrysene	0.21	0.17	0.16	0.20	0.19	0.18
dimethylchrysene	0.24	0.23	0.21	0.29	0.27	0.26
trimethylchrysene	0.29	0.23	0.24	0.30	0.25	0.35
tetramethylchrysene	0.24	0.22	0.23	0.25	0.28	0.27
benzo[b]fluoranthene	0.08	0.09	0.08	0.10	0.11	
benzo[e]pyrene		0.07	0.06		0.08	
benzo[ghi]perylene	0.07	0.08	0.06	0.07	0.10	

Table S11 Linear regression statistics and t-tests for significant slopes as well as comparisons between slopes for experiments conducted using water collected from the Bedford Basin.

BB	Linear Regressions																t-test for Significant difference between slopes				Rate constant (h-1)			Rate constant (d-1)	
	Light Incubations								Dark Incubations																
Analyte	Intercept	Slope	SlopeSE	df	R2	Fstat			Intercept	Slope	SlopeSE	df	R2	Fstat			Error	t statistic	df	p-value	Dark	Light	Photodegradation	Other Abiotic	Photodegradation
C13	2.39	-0.01	0.00	3	0.78	10.43	-3.23	0.05	2.39	0.00	0.00	3	0.24	0.92	-0.96	0.41	0.00	1.88	2	0.20		-0.01	-0.01		0.14
C14	2.50	-0.01	0.00	3	0.78	10.46	-3.23	0.05	2.50	0.00	0.00	3	0.36	1.71	-1.31	0.28	0.00	1.67	2	0.24		-0.01	-0.01		0.14
C15	2.52	-0.01	0.00	3	0.76	9.75	-3.12	0.05	2.52	0.00	0.00	3	0.39	1.94	-1.39	0.26	0.00	1.63	2	0.25					
C16	2.59	0.00	0.00	3	0.71	7.22	-2.69	0.07	2.59	0.00	0.00	3	0.23	0.88	-0.94	0.42	0.00	1.30	2	0.32					
C17	2.72	0.00	0.00	3	0.87	20.31	-4.51	0.02	2.72	0.00	0.00	3	0.00	0.01	-0.10	0.92	0.00	1.81	2	0.21		0.00	0.00		0.08
C18	2.36	0.00	0.00	3	0.70	6.89	-2.63	0.08	2.36	0.00	0.00	3	0.09	0.29	-0.54	0.63	0.00	1.39	2	0.30					
C19	2.29	0.00	0.00	3	0.67	5.98	-2.44	0.09	2.29	0.00	0.00	3	0.20	0.74	-0.86	0.45	0.00	1.09	2	0.39					
C20	2.21	0.00	0.00	3	0.69	6.82	-2.61	0.08	2.21	0.00	0.00	3	0.23	0.91	-0.95	0.41	0.00	1.41	2	0.29					
C21	2.14	0.00	0.00	3	0.69	6.67	-2.58	0.08	2.14	0.00	0.00	3	0.31	1.33	-1.15	0.33	0.00	1.32	2	0.32					
C22	2.08	0.00	0.00	3	0.72	7.86	-2.80	0.07	2.08	0.00	0.00	3	0.35	1.59	-1.26	0.30	0.00	1.28	2	0.33					
C23	2.02	0.00	0.00	3	0.73	8.23	-2.87	0.06	2.02	0.00	0.00	3	0.32	1.39	-1.18	0.32	0.00	1.37	2	0.30					
C24	2.07	0.00	0.00	3	0.73	8.00	-2.83	0.07	2.07	0.00	0.00	3	0.24	0.95	-0.97	0.40	0.00	1.30	2	0.32					
C25	1.86	0.00	0.00	3	0.79	11.56	-3.40	0.04	1.86	0.00	0.00	3	0.40	1.97	-1.40	0.26	0.00	1.57	2	0.26		0.00	0.00		0.09
C26	1.83	0.00	0.00	3	0.73	8.06	-2.84	0.07	1.83	0.00	0.00	3	0.23	0.90	-0.95	0.41	0.00	1.45	2	0.29					
C27	1.73	0.00	0.00	3	0.75	8.82	-2.97	0.06	1.73	0.00	0.00	3	0.31	1.33	-1.15	0.33	0.00	1.52	2	0.27					
C28	1.61	0.00	0.00	3	0.74	8.43	-2.90	0.06	1.61	0.00	0.00	3	0.35	1.59	-1.26	0.30	0.00	1.22	2	0.35					
C29	1.43	0.00	0.00	3	0.74	8.63	-2.94	0.06	1.43	0.00	0.00	3	0.31	1.35	-1.16	0.33	0.00	1.36	2	0.31					
C30	1.00	0.00	0.00	3	0.49	2.86	-1.69	0.19	1.00	0.00	0.00	3	0.06	0.20	-0.44	0.69	0.00	0.91	2	0.46					
C31	0.86	0.00	0.00	3	0.74	8.47	-2.91	0.06	0.86	0.00	0.00	3	0.32	1.40	-1.18	0.32	0.00	1.36	2	0.31					
C32	1.04	0.00	0.00	3	0.67	6.00	-2.45	0.09	1.04	0.00	0.00	3	0.41	2.08	-1.44	0.24	0.00	1.13	2	0.38					
C33	0.99	0.00	0.00	3	0.74	8.57	-2.93	0.06	0.99	0.00	0.00	3	0.01	0.02	-0.15	0.89	0.00	1.78	2	0.22					
C34	0.83	-0.01	0.00	3	0.86	18.35	-4.28	0.02	0.83	0.00	0.00	3	0.95	52.32	-7.23	0.01	0.00	2.67	2	0.12	0.00	-0.01		0.06	
C35	0.40	-0.01	0.00	3	0.89	23.09	-4.81	0.02	0.40	0.00	0.00	3	0.35	1.61	-1.27	0.29	0.00	2.71	2	0.11		-0.01	-0.01		0.17
naphthalene	1.97	0.00	0.00	3	0.64	5.30	-2.30	0.10	1.97	0.00	0.00	3	0.00	0.01	-0.07	0.95	0.00	1.55	2	0.26					
methylnaphthalene	2.83	0.00	0.00	3	0.65	5.52	-2.35	0.10	2.83	0.00	0.00	3	0.00	0.01	-0.10	0.93	0.00	1.56	2	0.26					
dimethylnaphthalene	3.51	0.00	0.00	3	0.75	8.82	-2.97	0.06	3.51	0.00	0.00	3	0.06	0.19	-0.44	0.69	0.00	1.88	2	0.20					
trimethylnaphthalene	3.46	-0.01	0.00	3	0.86	18.77	-4.33	0.02	3.46	0.00	0.00	3	0.19	0.71	-0.84	0.46	0.00	2.86	2	0.10		-0.01	-0.01		0.18
tetramethylnaphthalene	3.08	-0.01	0.00	3	0.87	19.96	-4.47	0.02	3.08	0.00	0.00	3	0.37	1.74	-1.32	0.28	0.00	2.69	2	0.12		-0.01	-0.01		0.18
acenaphthene	-0.97	-0.01	0.00	3	0.86	17.88	-4.23	0.02	-0.97	0.00	0.00	3	0.11	0.38	-0.62	0.58	0.00	2.73	2	0.11		-0.01	-0.01		0.16
fluorene	0.64	-0.01	0.00	3	0.82	14.12	-3.76	0.03	0.64	0.00	0.00	3	0.15	0.53	-0.73	0.52	0.00	2.43	2	0.14		-0.01	-0.01		0.15
methylfluorene	1.22	-0.01	0.00	3	0.88	21.50	-4.64	0.02	1.22	0.00	0.00	3	0.14	0.48	-0.69	0.54	0.00	2.76	2	0.11		-0.01	-0.01		0.16
dimethylfluorene	1.42	-0.01	0.00	3	0.85	17.43	-4.18	0.03	1.42	0.00	0.00	3	0.30	1.26	-1.12	0.34	0.00	2.71	2	0.11		-0.01	-0.01		0.19
trimethylfluorene	1.46	-0.01	0.00	3	0.90	26.46	-5.14	0.01	1.46	0.00	0.00	3	0.21	0.77	-0.88	0.44	0.00	3.07	2	0.09		-0.01	-0.01		0.16
dibenzothiophene	0.45	0.00	0.00	3	0.76	9.59	-3.10	0.05	0.45	0.00	0.00	3	0.23	0.88	-0.94	0.42	0.00	1.51	2	0.27					
methyldibenzothiophene	1.29	0.00	0.00	3	0.81	13.15	-3.63	0.04	1.29	0.00	0.00	3	0.22	0.85	-0.92	0.42	0.00	1.79	2	0.22		0.00	0.00		0.11
dimethyldibenzothiophene	1.83	0.00	0.00	3	0.75	8.83	-2.97	0.06	1.83	0.00	0.00	3	0.19	0.68	-0.83	0.47	0.00	1.42	2	0.29					
trimethyldibenzothiophene	1.73	0.00	0.00	3	0.72	7.74	-2.78	0.07	1.73	0.00	0.00	3	0.13	0.44	-0.67	0.55	0.00	1.42	2	0.29					
tetramethyldibenzothiophene	1.31	0.00	0.00	3	0.80	12.17	-3.49	0.04	1.31	0.00	0.00	3	0.21	0.78	-0.88	0.44	0.00	1.82	2	0.21		0.00	0.00		0.09
phenanthrene	1.48	0.00	0.00	3	0.70	6.85	-2.62	0.08	1.48	0.00	0.00	3	0.15	0.52	-0.72	0.52	0.00	1.34	2	0.31					
methylphenanthrene	2.22	0.00	0.00	3	0.72	7.85	-2.80	0.07	2.22	0.00	0.00	3	0.17	0.60	-0.78	0.49	0.00	1.39	2	0.30					
dimethylphenanthrene	2.43	0.00	0.00	3	0.74	8.60	-2.93	0.06	2.43	0.00	0.00	3	0.19	0.69	-0.83	0.47	0.00	1.48	2	0.28					
trimethylphenanthrene	2.29	0.00	0.00	3	0.83	14.72	-3.84	0.03	2.29	0.00	0.00	3	0.26	1.07	-1.04	0.38	0.00	1.85	2	0.21		0.00	0.00		0.11
tetramethylphenanthrene	1.88	-0.01	0.00	3	0.88	21.68	-4.66	0.02	1.88	0.00	0.00	3	0.23	0.87	-0.93	0.42	0.00	2.33	2	0.15		-0.01	-0.01		0.13
fluoranthene	-1.12	0.00	0.00	3	0.64	5.38	-2.32	0.10	-1.12	0.00	0.00	3	0.12	0.39	-0.63	0.58	0.00	1.09	2	0.39					
pyrene	-0.63	0.00	0.00	3	0.82	14.09	-3.75	0.03	-0.63	0.00	0.00	3	0.13	0.43	-0.66	0.56	0.00	1.48	2	0.28		0.00	0.00		0.09
methylpyrene	0.73	-0.01	0.00	3	0.95	57.16	-7.56	0.00	0.73	0.00	0.00	3	0.27	1.10	-1.05	0.37	0.00	2.12	2	0.17		-0.01	-0.01		0.17
dimethylpyrene	0.65	-0.01	0.00	3	0.89	24.33	-4.93	0.02	0.65	0.00	0.00	3	0.29	1.21	-1.10	0.35	0.00	3.07	2	0.09		-0.01	-0.01		0.14
trimethylpyrene	0.68	-0.01	0.00	3	0.86	17.92	-4.23	0.02	0.68	0.00	0.00	3	0.17	0.62	-0.79	0.49	0.00	2.14	2	0.17		-0.01	-0.01		0.16
tetramethylpyrene	0.84	0.00	0.00	3	0.61	4.66	-2.16	0.12	0.84	0.00	0.00	3	0.25	1.01	-1.00	0.39	0.00	0.73	2	0.54					
naphthobenzothiophene	-0.58	0.00	0.00	3	0.80	12.15	-3.49	0.04	-0.58	0.00	0.00	3	0.20	0.75	-0.86	0.45	0.00	1.65	2	0.24		0.00	0.00		0.12
methylnaphthobenzothiophene	0.89	0.00	0.00	3	0.76	9.33	-3.05	0.06	0.89	0.00	0.00	3	0.12	0.40	-0.63	0.57	0.00	1.44	2	0.29					
dimethylNBenzothiophene	1.23	0.00	0.00	3	0.70	7.14	-2.67	0.08	1.23	0.00	0.00	3	0.17	0.62	-0.78	0.49	0.00	1.27	2	0.33					
trimethylNbenzothiophene	1.02	0.00	0.00	3	0.77	9.90	-3.15	0.05	1.02	0.00	0.00	3	0.16	0.59	-0.77	0.50	0.00	1.66	2	0.24					
tetramethylNbenzothiophene	0.51	0.00	0.00	3	0.72	7.54	-2.75	0.07	0.51	0.00	0.00	3	0.23	0.89	-0.94	0.42	0.00	1.63	2	0.24					
benz[a]anthracene	-1.83	-0.01	0.00	3	0.98	152.86	-12.36	0.00	-1.83	0.00	0.00	3	0.11	0.38	-0.62	0.58	0.00	2.97	2	0.10		-0.01	-0.01		0.22
chrysene	-0.34	0.00	0.00	3	0.69	6.83	-2.61	0.08	-0.34	0.00	0.00	3	0.10	0.35	-0.59	0.60	0.00	1.26	2	0.33					
methylchrysene	0.53	-0.01	0.00	3	0.89	25.09	-5.01	0.02	0.53	0.00	0.00	3	0.34	1.53	-1.24	0.30	0.00	2.75	2	0.11		-0.01	-0.01		0.18
dimethylchrysene	0.94	-0.01	0.00	3	0.93	39.41	-6.28	0.01	0.94	0.00	0.00	3	0.44	2.34	-1.53	0.22	0.00	3.09	2	0.09		-0.01	-0.01		0.23
trimethylchrysene	0.67	-0.01	0.00	3	0.98	117.28	-10.83	0.00	0.67	0.00	0.00	3	0.16	0.58	-0.76	0.50	0.00	4.36	2	0.05		-0.01	-0.01		0.27
tetramethylchrysene	0.42	-0.01	0.00	3	0.93	38.41	-6.20	0.01	0.42	0.00	0.00	3	0.01	0.02	-0.16	0.88	0.00	2.59	2	0.12		-0.01	-0.01		0.24
benzo[b]fluoranthene	-1.36	0.00	0.00	3	0.66	5.83	-2.42	0.09	-1.36	0.00	0.00	3	0.24	0.96	-0.98	0.40	0.00	1.29	2	0.33					
benzo[e]pyrene	-1.24	0.00	0.00	3	0.85	16.36	-4.04	0.03	-1.24	0.00	0.00	3	0.15	0.52	-0.72	0.52	0.00	1.78	2	0.22		0.00	0.00		0.10
benzo[a]pyrene	-2.36	-0.01	0.00	3	0.97	85.08	-9.22	0.00	-2.36	0.00	0.01	3	0.12	0.39	0.63	0.57	0.01	1.80	2	0.21		-0.01	-0.01		0.20
indeno[1,2,3-cd]pyrene	-2.47	0.00	0.00	3	0.94	44.84	-6.70	0.01	-2.47	0.00	0.00	3	0.82	13.63	-3.69	0.03	0.00	3.29	2	0.08	0.00	0.00		0.04	
dibenz[a,h]anthracene	-2.74	-0.01	0.00	3	0.97	106.94	-10.34	0.00	-2.74	0.00	0.00	3	0.62	4.89	-2.21	0.11	0.00	5.47	2	0.03		-0.01	-0.01		0.15
benzo[ghi]perylene	-2.05	0.00	0.00	3	0.86	19.13	-4.37	0.02	-2.05	0.00	0.00	3	0.23	0.92	-0.96	0.41	0.00	2.20	2	0.16		0.00	0.00		0.10

Table S12 Linear regression statistics and t-tests for significant slopes as well as comparisons between slopes for experiments conducted using milliQ water with oil added with and without lights.

milliQ	Linear Regressions																t-test for Significant difference between slopes				Rate constant (h-1)			Rate constant (d-1)	
	Light Incubations								Dark Incubations																
Analyte	Intercept	Slope	SlopeSE	df	R2	Fstat	t-value	p-value	Intercept	Slope	SlopeSE	df	R2	Fstat	t-value	p-value	Error	t statistic	df	p-value	Dark	Light	Photodegradation	Other Abiotic	Photodegradation
C13	2.56	-0.01	0.00	4	0.83	19.45	-4.41	0.01	2.56	0.00	0.00	4	0.37	2.36	1.54	0.20	0.00	4.27	4	0.01	na	-0.01	-0.01		0.13
C14	2.75	-0.01	0.00	4	0.91	41.94	-6.48	0.00	2.75	0.00	0.00	4	0.39	2.54	1.59	0.19	0.00	5.67	4	0.00	na	-0.01	-0.01		0.13
C15	2.73	0.00	0.00	4	0.78	13.97	-3.74	0.02	2.73	0.00	0.00	4	0.31	1.82	1.35	0.25	0.00	3.59	4	0.02	na	0.00	0.00		0.10
C16	2.70	0.00	0.00	4	0.63	6.82	-2.61	0.06	2.70	0.00	0.00	4	0.04	0.16	0.40	0.71	0.00	2.37	4	0.08	na	na	na		
C17	2.51	0.00	0.00	4	0.67	7.99	-2.83	0.05	2.51	0.00	0.00	4	0.02	0.09	0.31	0.78	0.00	2.45	4	0.07	na	0.00	0.00		0.09
C18	2.44	0.00	0.00	4	0.68	8.59	-2.93	0.04	2.44	0.00	0.00	4	0.04	0.18	0.42	0.69	0.00	2.58	4	0.06	na	0.00	0.00		0.08
C19	2.41	0.00	0.00	4	0.66	7.60	-2.76	0.05	2.41	0.00	0.00	4	0.01	0.02	0.15	0.89	0.00	2.28	4	0.08	na	na	na		
C20	2.11	0.00	0.00	4	0.76	12.88	-3.59	0.02	2.11	0.00	0.00	4	0.10	0.44	0.66	0.54	0.00	3.25	4	0.03	na	0.00	0.00		0.09
C21	2.08	0.00	0.00	4	0.80	16.17	-4.02	0.02	2.08	0.00	0.00	4	0.61	6.16	2.48	0.07	0.00	4.69	4	0.01	na	0.00	0.00		0.10
C22	2.00	0.00	0.00	4	0.75	11.88	-3.45	0.03	2.00	0.00	0.00	4	0.48	3.67	1.92	0.13	0.00	3.94	4	0.02	na	0.00	0.00		0.09
C23	1.94	0.00	0.00	4	0.73	10.60	-3.26	0.03	1.94	0.00	0.00	4	0.68	8.50	2.92	0.04	0.00	4.06	4	0.02	0.00	0.00	0.00	-0.03	0.12
C24	2.11	0.00	0.00	4	0.84	21.31	-4.62	0.01	2.11	0.00	0.00	4	0.42	2.86	1.69	0.17	0.00	4.92	4	0.01	na	0.00	0.00		0.12
C25	2.13	0.00	0.00	4	0.77	13.65	-3.70	0.02	2.13	0.00	0.00	4	0.85	22.39	4.73	0.01	0.00	4.43	4	0.01	0.00	0.00	0.00	-0.02	0.11
C26	2.29	0.00	0.00	4	0.75	12.06	-3.47	0.03	2.29	0.00	0.00	4	0.62	6.56	2.56	0.06	0.00	4.03	4	0.02	na	0.00	0.00		0.10
C27	2.38	0.00	0.00	4	0.77	13.59	-3.69	0.02	2.38	0.00	0.00	4	0.27	1.51	1.23	0.29	0.00	3.88	4	0.02	na	0.00	0.00		0.09
C28	2.21	0.00	0.00	4	0.72	10.39	-3.22	0.03	2.21	0.00	0.00	4	0.46	3.39	1.84	0.14	0.00	3.71	4	0.02	na	0.00	0.00		0.10
C29	2.29	0.00	0.00	4	0.84	21.76	-4.66	0.01	2.29	0.00	0.00	4	0.28	1.56	1.25	0.28	0.00	4.80	4	0.01	na	0.00	0.00		0.10
C30	1.16	0.00	0.00	4	0.70	9.52	-3.09	0.04	1.16	0.00	0.00	4	0.02	0.10	0.32	0.77	0.00	2.79	4	0.05	na	0.00	0.00		0.09
C31	1.04	-0.01	0.00	4	0.73	11.08	-3.33	0.03	1.04	0.00	0.00	4	0.06	0.26	-0.51	0.64	0.00	2.18	4	0.09	na	-0.01	-0.01		0.13
C32	0.79	-0.01	0.00	4	0.79	15.08	-3.88	0.02	0.79	0.00	0.00	4	0.05	0.21	-0.46	0.67	0.00	2.84	4	0.05	na	-0.01	-0.01		0.14
C33	0.52	-0.01	0.00	4	0.78	14.09	-3.75	0.02	0.52	0.00	0.00	4	0.14	0.66	-0.81	0.46	0.00	0.64	4	0.56	na	-0.01	-0.01		0.13
C34	1.25	-0.01	0.00	4	0.79	14.73	-3.84	0.02	1.25	0.00	0.00	3	0.37	1.78	-1.34	0.27	0.00	2.67	3	0.08	na	-0.01	-0.01		0.13
C35	1.02	0.00	0.00	4	0.67	8.06	-2.84	0.05	1.02	-0.01	0.01	4	0.12	0.56	-0.75	0.50	0.01	-0.08	4	0.94	na	0.00	0.00		0.11
naphthalene	2.04	0.00	0.00	4	0.33	1.99	-1.41	0.23	2.04	0.00	0.00	4	0.06	0.27	0.52	0.63	0.00	1.48	4	0.21	na	na	na		
methylnaphthalene	2.96	0.00	0.00	4	0.56	5.05	-2.25	0.09	2.96	0.00	0.00	4	0.09	0.39	0.63	0.56	0.00	2.30	4	0.08	na	na	na		
dimethylnaphthalene	3.66	0.00	0.00	4	0.75	12.12	-3.48	0.03	3.66	0.00	0.00	4	0.11	0.52	0.72	0.51	0.00	3.46	4	0.03	na	0.00	0.00		0.11
trimethylnaphthalene	3.58	0.00	0.00	4	0.72	10.42	-3.23	0.03	3.58	0.00	0.00	4	0.13	0.60	0.77	0.48	0.00	3.16	4	0.03	na	0.00	0.00		0.11
tetramethylnaphthalene	3.19	-0.01	0.00	4	0.80	16.21	-4.03	0.02	3.19	0.00	0.00	4	0.14	0.64	0.80	0.47	0.00	3.80	4	0.02	na	-0.01	-0.01		0.13
acenaphthene	-0.76	-0.01	0.00	4	0.67	8.21	-2.86	0.05	-0.76	0.00	0.00	4	0.00	0.02	0.13	0.90	0.00	2.73	4	0.05	na	-0.01	-0.01		0.13
fluorene	0.91	-0.01	0.00	4	0.79	14.70	-3.83	0.02	0.91	0.00	0.00	4	0.08	0.34	0.58	0.59	0.00	3.39	4	0.03	na	-0.01	-0.01		0.14
methylfluorene	1.47	-0.01	0.00	4	0.75	12.23	-3.50	0.02	1.47	0.00	0.00	4	0.01	0.06	0.24	0.82	0.00	3.03	4	0.04	na	-0.01	-0.01		0.14
dimethylfluorene	1.73	-0.01	0.00	4	0.84	21.39	-4.62	0.01	1.73	0.00	0.00	4	0.05	0.19	-0.44	0.68	0.00	3.28	4	0.03	na	-0.01	-0.01		0.17
trimethylfluorene	1.74	-0.01	0.00	4	0.81	17.12	-4.14	0.01	1.74	0.00	0.00	4	0.01	0.03	0.18	0.86	0.00	3.55	4	0.02	na	-0.01	-0.01		0.14
dibenzothiophene	0.67	0.00	0.00	4	0.72	10.27	-3.21	0.03	0.67	0.00	0.00	4	0.10	0.46	0.68	0.53	0.00	2.86	4	0.05	na	0.00	0.00		0.11
methyldibenzothiophene	1.65	-0.01	0.00	4	0.81	16.70	-4.09	0.02	1.65	0.00	0.00	4	0.01	0.04	-0.19	0.86	0.00	3.08	4	0.04	na	-0.01	-0.01		0.15
dimethyldibenzothiophene	2.16	0.00	0.00	4	0.75	11.76	-3.43	0.03	2.16	0.00	0.00	4	0.03	0.11	0.34	0.75	0.00	2.87	4	0.05	na	0.00	0.00		0.12
trimethyldibenzothiophene	2.04	0.00	0.00	4	0.76	12.36	-3.52	0.02	2.04	0.00	0.00	4	0.03	0.13	0.36	0.74	0.00	2.97	4	0.04	na	0.00	0.00		0.12
tetramethyldibenzothiophene	1.57	0.00	0.00	4	0.80	15.57	-3.95	0.02	1.57	0.00	0.00	4	0.03	0.11	0.34	0.75	0.00	2.92	4	0.04	na	0.00	0.00		0.11
phenanthrene	1.78	0.00	0.00	4	0.71	9.98	-3.16	0.03	1.78	0.00	0.00	4	0.07	0.29	0.54	0.62	0.00	2.78	4	0.05	na	0.00	0.00		0.10
methylphenanthrene	2.49	0.00	0.00	4	0.75	11.75	-3.43	0.03	2.49	0.00	0.00	4	0.07	0.32	0.57	0.60	0.00	3.01	4	0.04	na	0.00	0.00		0.11
dimethylphenanthrene	2.70	0.00	0.00	4	0.76	12.81	-3.58	0.02	2.70	0.00	0.00	4	0.04	0.19	0.43	0.69	0.00	3.07	4	0.04	na	0.00	0.00		0.12
trimethylphenanthrene	2.57	-0.01	0.00	4	0.80	16.24	-4.03	0.02	2.57	0.00	0.00	4	0.02	0.07	0.27	0.80	0.00	3.10	4	0.04	na	-0.01	-0.01		0.13
tetramethylphenanthrene	2.17	-0.01	0.00	4	0.80	15.69	-3.96	0.02	2.17	0.00	0.00	4	0.00	0.00	0.04	0.97	0.00	3.12	4	0.04	na	-0.01	-0.01		0.13
fluoranthene	-0.49	0.00	0.00	4	0.68	8.59	-2.93	0.04	-0.49	0.00	0.00	4	0.23	1.21	1.10	0.33	0.00	3.04	4	0.04	na	0.00	0.00		0.10
pyrene	-0.44	0.00	0.00	4	0.64	7.07	-2.66	0.06	-0.44	0.00	0.00	4	0.09	0.38	0.62	0.57	0.00	2.55	4	0.06	na	na	na		
methylpyrene	0.99	-0.01	0.00	4	0.79	15.51	-3.94	0.02	0.99	0.00	0.00	3	0.08	0.27	0.52	0.64	0.00	2.91	3	0.06	na	-0.01	-0.01		0.13
dimethylpyrene	0.98	-0.01	0.00	4	0.82	18.18	-4.26	0.01	0.98	0.00	0.00	4	0.00	0.02	-0.14	0.90	0.00	3.09	4	0.04	na	-0.01	-0.01		0.15
trimethylpyrene	0.81	-0.01	0.00	4	0.81	16.72	-4.09	0.01	0.81	0.00	0.00	4	0.08	0.32	0.57	0.60	0.00	3.71	4	0.02	na	-0.01	-0.01		0.14
tetramethylpyrene	1.47	-0.01	0.00	4	0.91	38.36	-6.19	0.00	1.47	0.00	0.00	4	0.13	0.59	-0.77	0.49	0.00	2.94	4	0.04	na	-0.01	-0.01		0.14
naphthobenzothiophene	-0.46	-0.01	0.00	4	0.82	18.49	-4.30	0.01	-0.46	0.00	0.00	4	0.11	0.50	0.71	0.52	0.00	3.77	4	0.02	na	-0.01	-0.01		0.13
methylnaphthobenzothiophene	0.94	0.00	0.00	4	0.68	8.42	-2.90	0.04	0.94	0.00	0.00	4	0.00	0.02	-0.14	0.90	0.00	2.30	4	0.08	na	0.00	0.00		0.10
dimethylNBenzothiophene	1.23	0.00	0.00	4	0.74	11.37	-3.37	0.03	1.23	0.00	0.00	4	0.06	0.25	0.50	0.64	0.00	2.94	4	0.04	na	0.00	0.00		0.11
trimethylNbenzothiophene	1.06	0.00	0.00	4	0.78	14.21	-3.77	0.02	1.06	0.00	0.00	4	0.06	0.25	0.50	0.65	0.00	3.34	4	0.03	na	0.00	0.00		0.11
tetramethylNbenzothiophene	0.42	0.00	0.00	4	0.71	9.73	-3.12	0.04	0.42	0.00	0.00	4	0.09	0.42	0.65	0.55	0.00	2.92	4	0.04	na	0.00	0.00		0.10
benz[a]anthracene	-1.75	0.00	0.00	4	0.85	22.89	-4.78	0.01	-1.75	0.00	0.00	3	0.01	0.05	-0.21	0.85	0.00	2.32	3	0.10	na	0.00	0.00		0.11
chrysene	-0.30	0.00	0.00	4	0.76	12.42	-3.52	0.02	-0.30	0.00	0.00	3	0.33	1.48	1.22	0.31	0.00	3.51	3	0.04	na	0.00	0.00		0.11
methylchrysene	0.60	-0.01	0.00	4	0.82	18.83	-4.34	0.01	0.60	0.00	0.00	4	0.06	0.27	0.52	0.63	0.00	3.79	4	0.02	na	-0.01	-0.01		0.15
dimethylchrysene	0.95	-0.01	0.00	4	0.85	22.89	-4.78	0.01	0.95	0.00	0.00	4	0.11	0.48	0.69	0.53	0.00	4.54	4	0.01	na	-0.01	-0.01		0.15
trimethylchrysene	0.77	-0.01	0.00	4	0.84	21.32	-4.62	0.01	0.77	0.00	0.00	4	0.01	0.02	0.15	0.89	0.00	4.07	4	0.02	na	-0.01	-0.01		0.18
tetramethylchrysene	0.54	-0.01	0.00	4	0.90	36.69	-6.06	0.00	0.54	0.00	0.00	4	0.06	0.24	-0.49	0.65	0.00	4.66	4	0.01	na	-0.01	-0.01		0.19
benzo[b]fluoranthene+benzo[k]fluoranthene	-0.87	0.00	0.00	4	0.69	8.77	-2.96	0.04	-0.87	0.00	0.00	4	0.09	0.41	0.64	0.56	0.00	2.78	4	0.05	na	0.00	0.00		0.08
benzo[e]pyrene	-0.96	0.00	0.00	4	0.73	10.89	-3.30	0.03	-0.96	0.00	0.00	4	0.01	0.04	-0.20	0.85	0.00	2.52	4	0.07	na	0.00	0.00		0.10
benzo[a]pyrene	-2.18	-0.01	0.00	4	0.86	23.75	-4.87	0.01	-2.18	0.00	0.00	4	0.82	17.79	4.22	0.01	0.00	6.35	4	0.00	0.00	-0.01	-0.01	-0.08	0.23
indeno[1,2,3-cd]pyrene	-2.13	0.00	0.00	4	0.79	14.79	-3.85	0.02	-2.13	0.00	0.00	4	0.00	0.00	-0.05	0.96	0.00	2.41	4	0.07	na	0.00	0.00		0.03
dibenz[a,h]anthracene	-1.92	0.00	0.00	4	0.73	11.04	-3.32	0.03	-1.92	0.00	0.00	4	0.01	0.03	-0.16	0.88	0.00	2.42	4	0.07	na	0.00			
benzo[ghi]perylene	-1.70	0.00	0.00	4	0.70	9.19	-3.03	0.04	-1.70	0.00	0.00	4	0.00	0.01	0.09	0.93	0.00	2.43	4	0.07	na	0.00	0.00		0.07

Table S13 Linear regression statistics and t-tests for significant slopes as well as comparisons between slopes for experiments conducted using milliQ water with the addition of mercuric chloride with oil added with and without lights.

milliQ+HgCl2	Linear Regressions																t-test for Significant difference between slopes				Rate constant (h-1)			Rate constant (d-1)	
	Light incubations								Dark Incubations																
Analyte	Intercept	Slope	SlopeSE	df	R2	Fstat	t-value	p-value	Intercept	Slope	SlopeSE	df	R2	Fstat	t-value	p-value	Error	t statistic	df	p-value	Dark	Light	Photodegradation	Other Abiotic	Photodegradation
C13	2.53	0.00	0.00	4	0.67	8.15	-2.86	0.05	2.53	0.00	0.00	4	0.06	0.24	0.49	0.65	0.00	2.20	4	0.09	na	0.00	0.00		0.08
C14	2.67	0.00	0.00	4	0.53	4.57	-2.14	0.10	2.67	0.00	0.00	4	0.33	1.96	1.40	0.23	0.00	2.49	4	0.07	na	na	na		
C15	2.72	0.00	0.00	4	0.71	9.67	-3.11	0.04	2.72	0.00	0.00	4	0.55	4.88	2.21	0.09	0.00	3.74	4	0.02	na	0.00	0.00		0.07
C16	2.64	0.00	0.00	4	0.70	9.32	-3.05	0.04	2.64	0.00	0.00	4	0.05	0.21	0.46	0.67	0.00	1.91	4	0.13	na	0.00	0.00		0.05
C17	2.42	0.00	0.00	4	0.64	6.98	-2.64	0.06	2.42	0.00	0.00	4	0.01	0.03	-0.16	0.88	0.00	0.90	4	0.42	na	na	na		
C18	2.38	0.00	0.00	4	0.60	5.93	-2.44	0.07	2.38	0.00	0.00	4	0.01	0.03	0.18	0.86	0.00	1.35	4	0.25	na	na	na		
C19	2.36	0.00	0.00	4	0.68	8.41	-2.90	0.04	2.36	0.00	0.00	4	0.00	0.01	-0.11	0.92	0.00	1.34	4	0.25	na	0.00	0.00		0.05
C20	2.10	0.00	0.00	4	0.82	18.18	-4.26	0.01	2.10	0.00	0.00	4	0.06	0.25	0.50	0.65	0.00	2.84	4	0.05	na	0.00	0.00		0.07
C21	2.05	0.00	0.00	4	0.75	11.91	-3.45	0.03	2.05	0.00	0.00	4	0.20	1.03	1.02	0.37	0.00	2.96	4	0.04	na	0.00	0.00		0.06
C22	1.99	0.00	0.00	4	0.73	10.61	-3.26	0.03	1.99	0.00	0.00	4	0.15	0.70	0.84	0.45	0.00	2.85	4	0.05	na	0.00	0.00		0.06
C23	1.93	0.00	0.00	4	0.92	45.73	-6.76	0.00	1.93	0.00	0.00	4	0.37	2.37	1.54	0.20	0.00	5.55	4	0.01	na	0.00	0.00		0.08
C24	2.07	0.00	0.00	4	0.81	17.33	-4.16	0.01	2.07	0.00	0.00	4	0.18	0.90	0.95	0.40	0.00	3.69	4	0.02	na	0.00	0.00		0.08
C25	2.12	0.00	0.00	4	0.92	47.28	-6.88	0.00	2.12	0.00	0.00	4	0.07	0.32	0.57	0.60	0.00	4.61	4	0.01	na	0.00	0.00		0.08
C26	2.26	0.00	0.00	4	0.88	29.58	-5.44	0.01	2.26	0.00	0.00	4	0.04	0.15	0.38	0.72	0.00	3.59	4	0.02	na	0.00	0.00		0.08
C27	2.37	0.00	0.00	4	0.91	41.41	-6.43	0.00	2.37	0.00	0.00	4	0.02	0.10	-0.31	0.77	0.00	3.99	4	0.02	na	0.00	0.00		0.10
C28	2.21	-0.01	0.00	4	0.78	14.53	-3.81	0.02	2.21	0.00	0.00	4	0.11	0.51	0.71	0.52	0.00	3.39	4	0.03	na	-0.01	-0.01		0.13
C29	2.26	0.00	0.00	4	0.79	15.10	-3.89	0.02	2.26	0.00	0.00	4	0.00	0.01	0.12	0.91	0.00	2.66	4	0.06	na	0.00	0.00		0.11
C30	1.05	0.00	0.00	4	0.12	0.53	-0.73	0.51	1.05	0.00	0.00	4	0.05	0.20	0.45	0.68	0.00	0.85	4	0.44	na	na	na		
C31	0.90	0.00	0.00	4	0.76	12.65	-3.56	0.02	0.90	0.00	0.00	4	0.04	0.17	0.41	0.70	0.00	2.76	4	0.05	na	0.00	0.00		0.08
C32	0.64	-0.01	0.00	4	0.94	64.00	-8.00	0.00	0.64	0.00	0.00	4	0.01	0.05	-0.23	0.83	0.00	2.92	4	0.04	na	-0.01	-0.01		0.14
C33	0.46	0.00	0.00	4	0.94	63.21	-7.95	0.00	0.46	0.00	0.00	4	0.05	0.22	-0.47	0.66	0.00	3.20	4	0.03	na	0.00	0.00		0.11
C34	1.06	-0.01	0.00	4	0.74	11.16	-3.34	0.03	1.06	0.00	0.00	4	0.05	0.19	0.44	0.69	0.00	2.62	4	0.06	na	-0.01	-0.01		0.12
C35	0.92	0.00	0.00	4	0.72	10.49	-3.24	0.03	0.92	0.00	0.00	4	0.05	0.21	0.46	0.67	0.00	2.12	4	0.10	na	0.00	0.00		0.08
naphthalene	1.95	0.00	0.00	4	0.69	8.98	-3.00	0.04	1.95	0.00	0.00	4	0.29	1.61	-1.27	0.27	0.00	1.09	4	0.34	na	0.00	0.00		0.07
methylnaphthalene	2.87	0.00	0.00	4	0.67	8.00	-2.83	0.05	2.87	0.00	0.00	4	0.12	0.53	-0.73	0.51	0.00	1.09	4	0.34	na	0.00	0.00		0.06
dimethylnaphthalene	3.55	0.00	0.00	4	0.63	6.81	-2.61	0.06	3.55	0.00	0.00	4	0.02	0.08	-0.29	0.79	0.00	1.24	4	0.28	na	na	na		
trimethylnaphthalene	3.50	0.00	0.00	4	0.81	16.76	-4.09	0.01	3.50	0.00	0.00	4	0.00	0.02	-0.13	0.90	0.00	2.27	4	0.09	na	0.00	0.00		0.09
tetramethylnaphthalene	3.11	0.00	0.00	4	0.76	12.92	-3.59	0.02	3.11	0.00	0.00	4	0.08	0.33	0.57	0.60	0.00	3.06	4	0.04	na	0.00	0.00		0.12
acenaphthene	-0.85	0.00	0.00	4	0.84	20.85	-4.57	0.01	-0.85	0.00	0.00	4	0.12	0.54	-0.73	0.50	0.00	2.25	4	0.09	na	0.00	0.00		0.12
fluorene	0.83	-0.01	0.00	4	0.86	25.23	-5.02	0.01	0.83	0.00	0.00	4	0.03	0.13	0.36	0.74	0.00	3.54	4	0.02	na	-0.01	-0.01		0.12
methylfluorene	1.40	-0.01	0.00	4	0.90	37.48	-6.12	0.00	1.40	0.00	0.00	4	0.03	0.11	-0.33	0.76	0.00	3.20	4	0.03	na	-0.01	-0.01		0.14
dimethylfluorene	1.62	-0.01	0.00	4	0.91	40.78	-6.39	0.00	1.62	0.00	0.00	4	0.05	0.21	-0.45	0.67	0.00	3.81	4	0.02	na	-0.01	-0.01		0.13
trimethylfluorene	1.71	0.00	0.00	4	0.81	17.22	-4.15	0.01	1.71	0.00	0.00	4	0.08	0.37	-0.60	0.58	0.00	2.51	4	0.07	na	0.00	0.00		0.10
dibenzothiophene	0.63	0.00	0.00	4	0.78	14.17	-3.76	0.02	0.63	0.00	0.00	4	0.00	0.00	-0.05	0.96	0.00	2.29	4	0.08	na	0.00	0.00		0.08
methyldibenzothiophene	1.49	0.00	0.00	4	0.76	12.53	-3.54	0.02	1.49	0.00	0.00	4	0.03	0.14	0.38	0.73	0.00	2.50	4	0.07	na	0.00	0.00		0.08
dimethyldibenzothiophene	2.11	0.00	0.00	4	0.79	15.01	-3.87	0.02	2.11	0.00	0.00	4	0.02	0.06	-0.25	0.81	0.00	2.17	4	0.10	na	0.00	0.00		0.08
trimethyldibenzothiophene	1.98	0.00	0.00	4	0.84	20.71	-4.55	0.01	1.98	0.00	0.00	4	0.02	0.07	-0.27	0.80	0.00	2.40	4	0.07	na	0.00	0.00		0.08
tetramethyldibenzothiophene	1.52	0.00	0.00	4	0.73	10.82	-3.29	0.03	1.52	0.00	0.00	4	0.06	0.27	-0.52	0.63	0.00	1.44	4	0.22	na	0.00	0.00		0.07
phenanthrene	1.74	0.00	0.00	4	0.76	12.55	-3.54	0.02	1.74	0.00	0.00	4	0.00	0.02	-0.13	0.90	0.00	2.09	4	0.10	na	0.00	0.00		0.07
methylphenanthrene	2.45	0.00	0.00	4	0.80	16.50	-4.06	0.02	2.45	0.00	0.00	4	0.01	0.04	-0.21	0.85	0.00	2.31	4	0.08	na	0.00	0.00		0.08
dimethylphenanthrene	2.65	0.00	0.00	4	0.82	17.83	-4.22	0.01	2.65	0.00	0.00	4	0.01	0.05	-0.23	0.83	0.00	2.41	4	0.07	na	0.00	0.00		0.08
trimethylphenanthrene	2.50	0.00	0.00	4	0.80	15.88	-3.98	0.02	2.50	0.00	0.00	4	0.00	0.00	0.01	0.99	0.00	2.77	4	0.05	na	0.00	0.00		0.09
tetramethylphenanthrene	2.10	0.00	0.00	4	0.81	17.57	-4.19	0.01	2.10	0.00	0.00	4	0.03	0.14	-0.37	0.73	0.00	2.53	4	0.06	na	0.00	0.00		0.09
fluoranthene	-0.51	0.00	0.00	4	0.84	21.51	-4.64	0.01	-0.51	0.00	0.00	4	0.03	0.11	-0.33	0.76	0.00	2.42	4	0.07	na	0.00	0.00		0.08
pyrene	-0.46	0.00	0.00	4	0.43	3.00	-1.73	0.16	-0.46	0.00	0.00	4	0.01	0.04	-0.20	0.85	0.00	1.03	4	0.36	na	na	na		
methylpyrene	0.93	0.00	0.00	4	0.89	31.18	-5.58	0.01	0.93	0.00	0.00	4	0.01	0.02	0.15	0.89	0.00	3.47	4	0.03	na	0.00	0.00		0.09
dimethylpyrene	0.86	0.00	0.00	4	0.88	29.17	-5.40	0.01	0.86	0.00	0.00	4	0.10	0.45	-0.67	0.54	0.00	3.03	4	0.04	na	0.00	0.00		0.11
trimethylpyrene	0.75	-0.01	0.00	4	0.88	29.76	-5.46	0.01	0.75	0.00	0.00	4	0.01	0.04	0.21	0.85	0.00	3.92	4	0.02	na	-0.01	-0.01		0.13
tetramethylpyrene	1.41	0.00	0.00	4	0.91	39.15	-6.26	0.00	1.41	0.00	0.00	4	0.23	1.21	-1.10	0.33	0.00	2.71	4	0.05	na	0.00	0.00		0.12
naphthobenzothiophene	-0.57	0.00	0.00	4	0.70	9.46	-3.08	0.04	-0.57	0.00	0.00	4	0.12	0.55	0.74	0.50	0.00	2.36	4	0.08	na	0.00	0.00		0.08
methylnaphthobenzothiophene	0.81	0.00	0.00	4	0.55	4.99	-2.23	0.09	0.81	0.00	0.00	4	0.01	0.03	0.17	0.88	0.00	1.06	4	0.35	na	na	na		
dimethylNBenzothiophene	1.14	0.00	0.00	4	0.69	9.10	-3.02	0.04	1.14	0.00	0.00	4	0.05	0.21	0.45	0.67	0.00	2.07	4	0.11	na	0.00	0.00		0.06
trimethylNbenzothiophene	0.98	0.00	0.00	4	0.73	11.05	-3.32	0.03	0.98	0.00	0.00	4	0.00	0.00	0.04	0.97	0.00	1.89	4	0.13	na	0.00	0.00		0.08
tetramethylNbenzothiophene	0.33	0.00	0.00	4	0.61	6.22	-2.49	0.07	0.33	0.00	0.00	4	0.05	0.20	0.44	0.68	0.00	1.87	4	0.14	na	na	na		
benz[a]anthracene	-1.88	0.00	0.00	4	0.91	38.59	-6.21	0.00	-1.88	0.00	0.00	4	0.63	6.95	-2.64	0.06	0.00	2.47	4	0.07	na	0.00	0.00		0.11
chrysene	-0.35	0.00	0.00	4	0.69	8.79	-2.97	0.04	-0.35	0.00	0.00	4	0.18	0.87	0.93	0.40	0.00	2.43	4	0.07	na	0.00	0.00		0.06
methylchrysene	0.50	0.00	0.00	4	0.86	24.31	-4.93	0.01	0.50	0.00	0.00	4	0.19	0.94	0.97	0.39	0.00	3.64	4	0.02	na	0.00	0.00		0.11
dimethylchrysene	0.86	0.00	0.00	4	0.87	26.01	-5.10	0.01	0.86	0.00	0.00	4	0.05	0.21	0.45	0.67	0.00	2.93	4	0.04	na	0.00	0.00		0.12
trimethylchrysene	0.63	-0.01	0.00	4	0.83	19.96	-4.47	0.01	0.63	0.00	0.00	4	0.09	0.38	0.62	0.57	0.00	3.52	4	0.02	na	-0.01	-0.01		0.17
tetramethylchrysene	0.42	-0.01	0.00	4	0.93	52.39	-7.24	0.00	0.42	0.00	0.00	4	0.01	0.05	0.21	0.84	0.00	4.30	4	0.01	na	-0.01	-0.01		0.17
benzo[b]fluoranthene+benzo[k]fluoranthene	-0.93	0.00	0.00	4	0.72	10.49	-3.24	0.03	-0.93	0.00	0.00	4	0.00	0.00	-0.04	0.97	0.00	1.60	4	0.19	na	0.00	0.00		0.05
benzo[e]pyrene	-1.07	0.00	0.00	4	0.42	2.92	-1.71	0.16	-1.07	0.00	0.00	4	0.47	3.58	-1.89	0.13	0.00	-0.24	4	0.82	na	na	na		
benzo[a]pyrene	-2.09	-0.01	0.00	4	0.99	291.10	-17.06	0.00	-2.09	0.00	0.00	4	0.88	29.83	5.46	0.01	0.00	13.06	4	0.00	0.00	-0.01	-0.01	-0.12	0.33
indeno[1,2,3-cd]pyrene	-2.09	0.00	0.00	4	0.86	25.54	-5.05	0.01	-2.09	0.00	0.00	4	0.04	0.15	-0.38	0.72	0.00	3.41	4	0.03	na	0.00	0.00		0.07
dibenz[a,h]anthracene	-1.89	0.00	0.00	4	0.90	36.28	-6.02	0.00	-1.89	0.00	0.00	4	0.74	11.52	-3.39	0.03	0.00	3.77	4	0.02	0.00	0.00	0.00	0.03	0.07
benzo[ghi]perylene	-1.71	0.00	0.00	4	0.86	23.80	-4.88	0.01	-1.71	0.00	0.00	4	0.21	1.06	-1.03	0.36	0.00	1.58	4	0.19	na	0.00	0.00		0.08

Table S14 Estimated inital degradation rate constants in the due to other abiotic factors (dark incubations) and photodegradation (light-dark incubations) for killed samples from the Bedford Basin, with milliQ and with milliQ+HgCl2 (d-1). For details on the regressions and t-tests see Tables S1-13. Summary data reports the mean and standard deviation based on the number of analytes for which a rate constant could be estimated.

	**Bedford Basin**		**milliQ Water**		**milliQ Water + $HgCl_2$**	
**Analyte**	**Other Abiotic Degradation**	**Photodegradation**	**Other Abiotic Degradation**	**Photodegradation**	**Other Abiotic Degradation**	**Photodegradation**
Number of *n*-alkanes	1	5	2	21	0	19
Average (sd) rates - *n*-alkanes	0.06	0.12 (0.03)	-0.02 (0.00)	0.11 (0.02)	-	0.08 (0.02)
Number of PACs	1	25	1	40	2	40
Average (sd) rates - PACs	0.04	0.16 (0.05)	-0.08	0.12 (0.03)	-0.04 (0.11)	0.10 (0.05)
C13		0.14		0.13		0.08
C14		0.14		0.13		
C15				0.10		0.07
C16						0.05
C17		0.08		0.09		
C18				0.08		
C19						0.05
C20				0.09		0.07
C21				0.10		0.06
C22				0.09		0.06
C23			-0.03	0.12		0.08
C24				0.12		0.08
C25		0.09	-0.02	0.11		0.08
C26				0.10		0.08
C27				0.09		0.10
C28				0.10		0.13
C29				0.10		0.11
C30				0.09		
C31				0.13		0.08
C32				0.14		0.14

C33			0.13	0.11
C34	0.06		0.13	0.12
C35		0.17	0.11	0.08
naphthalene				0.07
methylnaphthalene				0.06
dimethylnaphthalene			0.11	
trimethylnaphthalene		0.18	0.11	0.09
tetramethylnaphthalene		0.18	0.13	0.12
acenaphthene		0.16	0.13	0.12
fluorene		0.15	0.14	0.12
methylfluorene		0.16	0.14	0.14
dimethylfluorene		0.19	0.17	0.13
trimethylfluorene		0.16	0.14	0.10
dibenzothiophene			0.11	0.08
methyldibenzothiophene		0.11	0.15	0.08
dimethyldibenzothiophene			0.12	0.08
trimethyldibenzothiophene			0.12	0.08
tetramethyldibenzothiophene		0.09	0.11	0.07
phenanthrene			0.10	0.07
methylphenanthrene			0.11	0.08
dimethylphenanthrene			0.12	0.08
trimethylphenanthrene		0.11	0.13	0.09
tetramethylphenanthrene		0.13	0.13	0.09
fluoranthene			0.10	0.08
pyrene		0.09		
methylpyrene		0.17	0.13	0.09
dimethylpyrene		0.14	0.15	0.11
trimethylpyrene		0.16	0.14	0.13
tetramethylpyrene			0.14	0.12
naphthobenzothiophene		0.12	0.13	0.08
methylnaphthobenzothiophene			0.10	

dimethylNBenzothiophene				0.11		0.06
trimethylNbenzothiophene				0.11		0.08
tetramethylNbenzothiophene				0.10		
benz[a]anthracene		0.22		0.11		0.11
chrysene				0.11		0.06
methylchrysene		0.18		0.15		0.11
dimethylchrysene		0.23		0.15		0.12
trimethylchrysene		0.27		0.18		0.17
tetramethylchrysene		0.24		0.19		0.17
benzo[b]fluoranthene + benzo[k]fluoranthene				0.08		0.05
benzo[e]pyrene		0.10		0.10		
benzo[a]pyrene		0.20	-0.08	0.23	-0.12	0.33
indeno[1,2,3-cd]pyrene	0.04			0.03		0.07
dibenz[a,h]anthracene		0.15			0.03	0.07
benzo[ghi]perylene		0.10		0.07		0.08

Table S15 Estimate of the number of CFU/mL in the seawater after incubation for 72 h at 22°C.

Incubation	Treatment	Number of Colonies	Volume Plated (mL)	CFU/mL
Dark	HgCl2	0	0.05	0
Dark	HgCl2	0	0.05	0
Dark	HgCl2	0	0.05	0
Light	HgCl2	0	0.05	0
Light	HgCl2	0	0.05	0
Light	HgCl2	0	0.05	0
Dark	Live	68	0.05	1360
Dark	Live	85	0.05	1700
Dark	Live	89	0.05	1780
Light	Live	55	0.05	1100
Light	Live	75	0.05	1500
Light	Live	60	0.05	1200
